# Targeting RuvBL1 disrupts mitochondrial metabolism and structure in hepatocellular carcinoma

**DOI:** 10.1016/j.jhepr.2026.101858

**Published:** 2026-04-17

**Authors:** Tommaso Mello, Irene Simeone, Alice Guida, Dimitri Papini, Francesca Begnozzi, Alice Santi, Daniele Guasti, Patrizia Nardini, Simone Polvani, Matteo Lulli, Oxana Bereshchenko, Elisabetta Ceni, Armando Curto, Paolo Pinton, Massimo Bonora, Andrea Galli

**Affiliations:** 1 Department of Clinical and Experimental Biomedical Sciences “Mario Serio”, University of Florence, Florence, Italy; 2 Department of Medical Sciences, Section of Experimental Medicine, and Laboratory for Technologies of Advanced Therapies (LTTA), University of Ferrara, Ferrara, Italy; 3 Department of Experimental and Clinical Medicine, Imaging Platform, University of Florence, Florence, Italy; 4 Department of Philosophy, Social Sciences, Humanities and Education, University of Perugia, Perugia, Italy; 5 Maria Cecilia Hospital, GVM Care & Research, Cotignola, Italy

**Keywords:** OXPHOS, ATP synthase, TCA cycle, Amino acids metabolism, Ketogenesis, Liver cancer, Metabolic reprogramming

## Abstract

**Background & Aims:**

The AAA+ ATPase RuvBL1 takes part in several biological processes, including chromatin remodelling and DNA repair, ribosome biogenesis, mTOR signalling, and oncogenic transformation. RUVBL1 overexpression correlates with poor survival in patients with hepatocellular carcinoma (HCC). We previously found that RuvBL1 is a key regulator of liver glucose metabolism in mice. Here, we aimed at disentangling the metabolic function of RuvBL1 in HCC cells.

**Methods:**

Non-transformed AML-12, primary mouse hepatocytes, HCC cell lines, and RuvBL1^hep-/-^ mice were used (n = 3). RuvBL1 was targeted by RNAi and by inhibition with CB-6644. Metabolomic profiling and mitochondrial functions were assessed by targeted GC/MS, Seahorse analysis, and ATP synthase activity. Mitochondrial morphology and membrane potential were investigated by fluorescence microscopy, high-content imaging, and transmission electron microscopy. Mitochondrial RuvBL1 was detected by WB, super-resolution microscopy, transmission electron microscopy, and proximity ligation assay. Human HCC and normal liver samples from The Cancer Genome Atlas and GTEx databases were used for *in-silico* analysis (T = 369, N = 160).

**Results:**

Targeting RuvBL1 impairs mitochondria-centred metabolic processes, including amino acid metabolism, TCA cycle, and oxidative phosphorylation. Inhibition of RuvBL1/2 activity induces loss of cristae integrity, mitochondrial hyperpolarisation and fragmentation, a phenotype paralleled by the hepatocytes of RuvBL1^hep-/-^ mice. We detected RuvBL1 in proximity to mitochondrial ATP synthase, a previously unreported localisation for this protein. Mechanistically, CB-6644 reduces ATP synthase-RuvBL1 interaction and impairs complex V activity even under a fuelled TCA cycle. In human HCC, higher RUVBL1 expression correlates with gene signatures associated with mitochondrial oxidative phosphorylation (FDR = 5.64e^-03^), ATP synthase complex (FDR = 6.03e^-03^), and poorer outcome (*p* = 2e^-07^).

**Conclusions:**

Targeting RuvBL1 impairs complex V activity, disrupting mitochondrial metabolic functions and structural integrity. The mitochondrial functions of RuvBL1 may inform novel therapeutic strategies in the fight against hepatocellular carcinoma.

**Impact and implications:**

Metabolic reprogramming is a key feature driving HCC onset, progression, and plasticity, contributing to treatment resistance and poor prognosis. RUVBL1 overexpression correlates with reduced survival of patients with HCC and has emerged as a potential metabolic modulator. In this study, we found that targeting RuvBL1 impairs its interaction with mitochondrial ATP synthase, disrupting mitochondrial metabolism and cristae structure. In human HCC samples, RUVBL1 expression correlates with hallmark mitochondrial metabolic processes. These findings may inform the development of targeted therapeutic approaches aimed at impairing the metabolic rewiring and plasticity of HCC.

## Highlights

• The AAA+ ATPase RuvBL1 localises to mitochondria and interacts with ATP synthase.
• Targeting RuvBL1 impairs mitochondrial structure and metabolism.
• In human HCC, RUVBL1 expression correlates with mitochondrial oxidative phosphorylation, ATP synthase complex and adverse clinical prognosis.

## Graphical abstract

**Figure:**
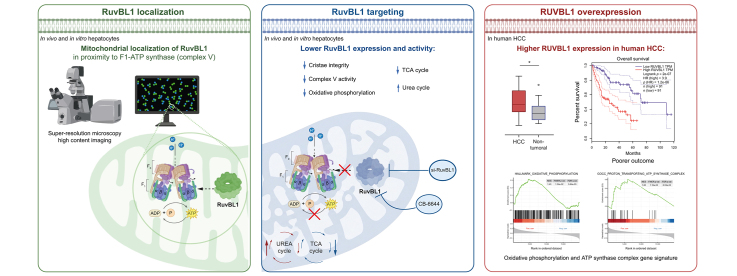

## Introduction

RuvBL1 (Pontin52) is an AAA+ ATPase involved in multiple cellular activities, including chromatin remodelling, telomere maintenance,^1^ ribosome biogenesis, and oncogenic transformation.[2], [3], [4] Overexpression of RUVBL1 and of the closely related RUVBL2 genes occurs frequently in human cancers,[5], [6], [7], [8], [9], [10] including hepatocellular carcinoma,^11^^,^^12^ and their higher expression correlates with reduced survival.

RuvBLs are increasingly drawing attention as potential targets for cancer treatment,[13], [14], [15] and the recently developed small-molecule inhibitor CB-6644, which targets RuvBL1/2 ATPase activity, showed promising results in a large panel of cancer cell lines.^16^

A notable function of RuvBL1/2 is to promote the expression of mTOR^17^ as well as the assembly of mTORC1 and mTORC2 complexes.^18^ Mechanistically, RuvBL1/2 are key components of the HSP90 co-chaperone complex R2TP, which, together with the TTT-chaperone, assists in the co-translational folding of mTOR and maturation of the mTORC1 complex.[18], [19], [20] In yeast, the R2TP complex participates in nutrient sensing, coordinating cell growth with nutrient availability.^21^ We have previously shown that the haploinsufficiency of RUVBL1 in mouse liver impairs mTOR expression and results in a metabolic phenotype characterised by hepatic insulin resistance, increased glucose output, hyperglycaemia, and increased blood triglycerides and cholesterol levels.^12^ Recently, Chen *et al.*^22^ uncovered HPCAL1 as a negative regulator of hepatocellular carcinoma (HCC) growth *in vivo,* which acts by binding to RuvBL1 and disrupting the TTT-RuvBL-mTORC1 interaction. Collectively, RuvBL1 and RuvBL2 are emerging as potent metabolic modulators and potential therapeutic targets for anticancer strategies.^12^^,^^16^^,^^20^

Hepatocellular carcinoma (HCC) is a multifaceted entity arising on top of different underlying liver conditions, aetiologies, and molecular drivers. Despite these diversities, metabolic reprogramming emerges as a shared characteristic that drives virtually any phase of HCC development, from onset to progression, resistance, and immune escape, offering potential leverage for precision medicine approaches, from diagnosis to treatment.[23], [24], [25], [26]

In this work, we aimed to deepen the understanding of RuvBL1’s metabolic role in HCC. We found that RuvBL1 is required for proper mitochondrial respiration and that targeting RuvBL1/2 activity results in a peculiar mitochondrial damage characterised by membrane hyperpolarisation, swelling, and loss of cristae integrity. We detected RuvBL1 into the mitochondria, a previously unreported localisation for this protein. Mechanistically, RuvBL1 and ATP synthase (complex V) are found in proximity, and treatment with CB-6644 reduces their interaction, impairing mitochondrial ATP production.

RUVBL1 expression in human HCC samples correlates with the expression of (F_1_)-ATP synthase subunits and metabolic genes involved in both glycolysis and oxidative phosphorylation (OXPHOS), with mTOR pathway activation, with a more advanced tumour stage and with reduced survival.

These results suggest that RuvBL1 overexpression in HCC is required to meet the increased metabolic demands of cancer cells, supporting mitochondrial activity for ATP generation and biosynthesis.

## Materials and methods

Please refer to the supplementary material and supplementary CTAT table for a complete materials and methods description.

### Animal model

All procedures involving laboratory animals were conducted in accordance with institutional ethical norms and national laws, after approval by the Italian Ministry of Health (decrees 30/2013 and 665/2018). The conditional knockout RuvBL1^hep-/-^ mice were created by crossing RuvBL1^floxed/floxed^ (RuvBL1^f/f^) mice^27^ with Albumin-Cre^tg/tg^ mice acquired from Jackson Laboratories through the local distributor (Charles River Italia). The first round of breeding generated RuvBL1^hep+/-12^ offspring, which were crossed again to produce RuvBL1^hep-/-^ mice. Three male mice per genotype were used for the transmission electron microscopy analysis in this study.

### Cell lines and primary hepatocytes

Authenticated Hepa1-6, Huh7, HepG2, Hep3B, and AML-12 cell lines were obtained from suppliers reported in the supplementary CTAT table. Upon arrival and at regular intervals thereafter, all cell lines were tested for *Mycoplasma* by PCR. All cell lines were maintained in culture without antibiotics. Primary hepatocytes were isolated from 3-month-old C57BL/6 mice by collagenase-dispase perfusion and Percoll gradient centrifugation. Primary hepatocytes were maintained on collagen-coated plates and layered with collagen I for sandwich culture.

RUVBL1 gene silencing was performed with 20 nM IBONI siRNA (RiboxX GmbH, Dresden, Germany) or with 5 nM Silencer Select validated siRNAs (Life Technologies Italia, Monza, Italy) using negative-control siRNA and GAPDH siRNA to evaluate the silencing efficiency and the transfection efficiency, respectively. INTERFERin (Polyplus-Sartorius, Göttingen, Germany) or RNAiMAX (Life Technologies Italia, Monza, Italy) were used as transfection reagents. RUVBL1/2 ATPase activity was targeted with the specific inhibitor CB-6644 (ChemScene, USA).

### Metabolomic analysis

Targeted gas chromatography-mass spectroscopy (GC-MS) metabolomics was performed on Huh7 cells cultured in complete medium and treated for 48 h with 0.5 μM of CB-6644 or vehicle alone. Metabolites were extracted with 80% cold methanol supplemented with 1% norvaline as internal standard. Dried extracts were derivatised in 10 μl of 40 mg/ml methoxamine hydrochloride in pyridine at 37 °C for 90 min, followed by 50 μl of MTBSTFA (Sigma-Adrich, MERCK, Italy) at 60 °C for 30 min. Data acquisition was performed using an Intuvo 9000 GC/5977B MS System (Agilent Technologies Italy) equipped with an HP-5MS capillary column (30 m × 0.25 mmx0.25 μm). For relative metabolite abundances, the peak area of each metabolite was normalised to norvaline and to protein concentration.

### Complex V activity

Complex V activity was assessed in Huh7 cells transiently transfected with a mitochondrially targeted luciferase chimera (mtLuc). After treatment with CB-6644, cells were incubated in a thermostated perfusion chamber, and real-time luminescence emission was recorded using a custom-built luminometer. Recordings were initiated with a 30-s baseline measurement in intracellular buffer (IB) designed to mimic the cytosolic ionic composition. Cells were then perfused with IB containing 25 μM luciferin (IBluc) to stabilise ATP-driven luciferase light production. After plasma membrane permeabilisation with 25 μM digitonin (Sigma-Aldrich, MERCK, Italy), cells were sequentially exposed to IBluc supplemented with 1 mM malic acid and 1 mM glutamic acid (Sigma-Aldrich, MERCK, Italy), followed by 5 mM ADP (Sigma-Aldrich, MERCK, Italy). The resulting increase in luminescence, measured in counts per second, reflected ATP synthesis driven by complex V activity in response to exogenous ADP.

### *In-silico* analysis

RUVBL1 expression in normal liver (The Cancer Genome Atlas [TCGA] and GTEx) and HCC samples of the TCGA_liver hepatocellular carcinoma (LIHC) cohort was evaluated through the GEPIA2 web tool, last accessed on 18 January 2026. Overall survival and most differential survival genes analyses in the LIHC cohort were performed in GEPIA2 using RUVBL1 expression quartiles (75–25%) as cut-off values for group definition. RUVBL1 expression levels across HCC stages were also graphed in GEPIA2.

Gene Set Enrichment Analysis (GSEA) on the LIHC cohort was performed through the web app GENI, using Spearman’s correlation method and default settings. The TCGA database was accessed via cBioPortal for Cancer Genomics to retrieve RuvBL1 mRNA expression data in the LIHC cohort. Based on RUVBL1 expression, patients were assigned to the HI_RUVBL1 (Z-score >2) or LOW_RUVBL1 (Z-score <2) group. The mRNA expression data of genes differentially expressed between the two groups was used to run a Gene Ontology (GO) analysis with ClueGO (Cytoscape app).

### Statistical analysis

Statistical analyses were performed with GraphPad Prism 10 (GraphPad Software, San Diego, CA, USA) on data from three or more replicates. The statistical significance levels achieved, and the types of tests used are reported in figure legends using standard notations: ∗*p* <0.05, ∗∗*p* <0.01, ∗∗∗*p* <0.001, and ∗∗∗∗*p* <0.0001. Exact *p* values for each analysis are reported in the Supplementary materials.

## Results

### RuvBL1/2 ATPase activity is required for mitochondrial metabolism

**Fig. 1:**
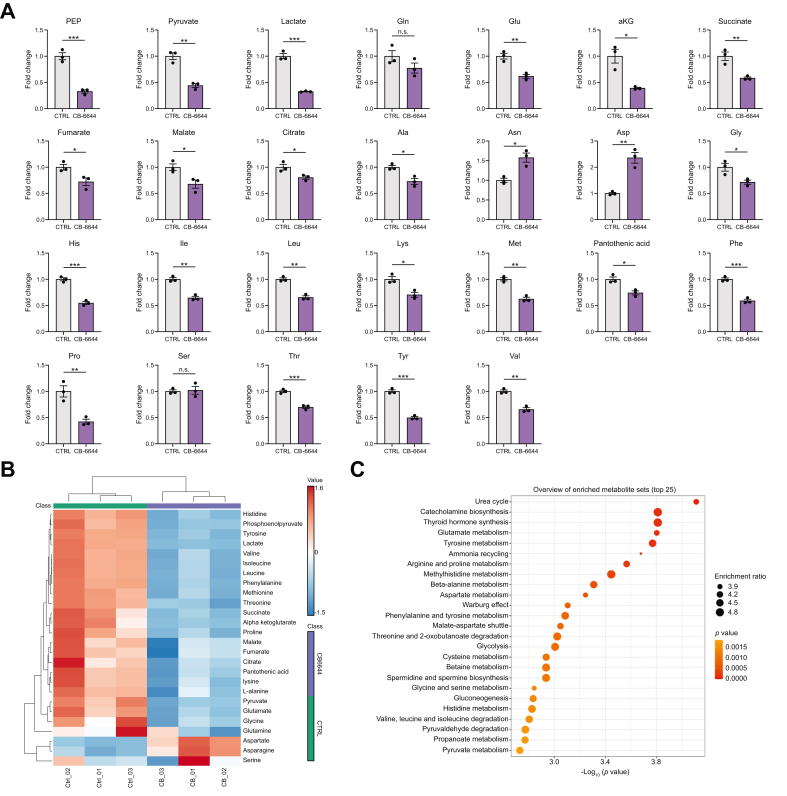
Targeting RuvBL1/2 ATPase activity impairs cellular metabolism. (A) Targeted metabolomic analysis of Huh7 cells treated with CB-6644 0.5 μM for 48 h. Normalised AUC values (mean ± SEM) from three independent experiments are reported. Unpaired Student’s *t* test was used for statistical significance. ∗*p* <0.05, ∗∗*p* <0.01, ∗∗∗*p* <0.001. (B) Hierarchical clustering of the analysed metabolites in CB-6644-treated Huh7 cells. (C) Enrichment analysis performed with Metaboanalyst in CB-6644-treated Huh7 cells.

To determine the extent of RuvBLs-regulated metabolic processes in human HCC cells, we conducted targeted metabolomic analysis by GC-MS in Huh7 cells treated with CB-6644 (0.5 μM for 48 h), a selective inhibitor of the RuvBL1/2 ATPase complex.^16^ The doses and duration of CB-6644 treatment used in this work were chosen based on published ranges^16^ and after preliminary verification that no significant cell death was occurring. Several key metabolites along the glycolytic and TCA cycle pathways, such as PEP, pyruvate, lactate, and virtually all TCA cycle intermediates, were significantly reduced by CB-6644 as shown in Fig. 1A, B. The levels of most of the analysed amino acids were also significantly decreased, with the noticeable exception of aspartate and asparagine, whose levels were clearly increased by CB-6644 administration (Fig. 1A, B).

Quantitative enrichment analysis performed with Metaboanalyst allowed a clear separation of CB-6644 treated cells *vs* controls by PCA (Fig. S1A), and identified several significantly enriched pathways, including the urea cycle, malate–aspartate shuttle, Warburg effect, and amino acids metabolism (Fig. 1C; Fig. S1B). Functional annotation of the CB-6644-modulated metabolite set using the Consensus Path Database^28^ (within KEGG, Reactome, and WikiPathways), confirmed significant enrichment in pathways involved in the regulation of energy metabolism and cancer metabolic reprogramming, as well as cytosolic and mitochondrial translation-related processes (Table S1). Interestingly, several of the most enriched pathways, such as the urea cycle, amino acids metabolism, and TCA cycle, are centred in or converge on mitochondria, suggesting that this organelle could be pivotal in the metabolic action mediated by RuvBL1/2 ATPase activity.

### RuvBL1 is required for OXPHOS

**Fig. 2:**
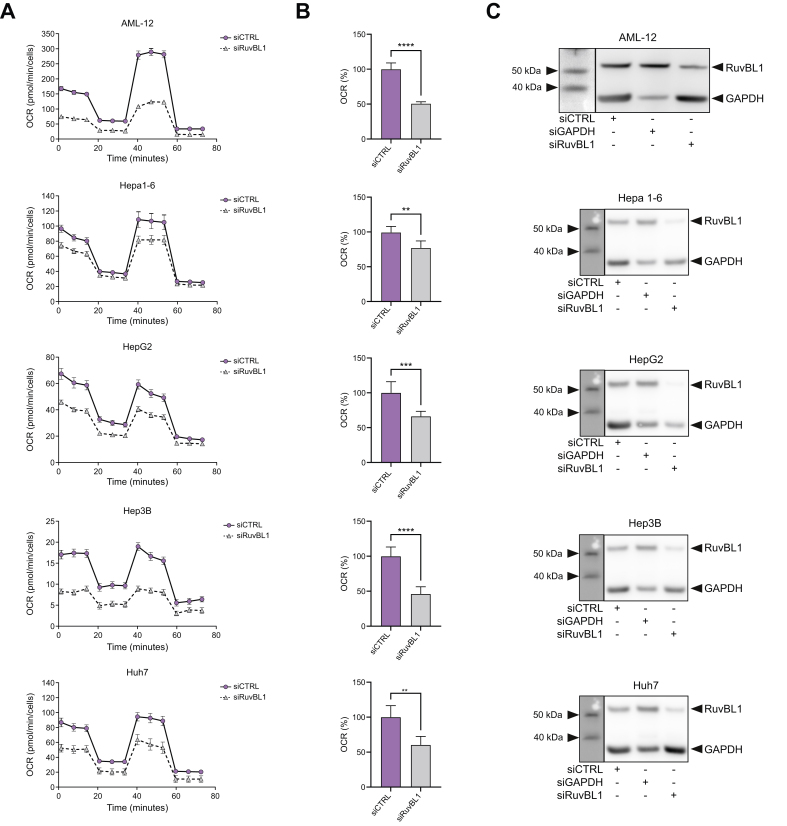
RuvBL1 knockdown impairs mitochondrial respiration. (A) Seahorse MitoStress test in RuvBL1-silenced cells. Representative normalised Oxygen Comsumption Rate (OCR) profiles (mean ± SEM). (B) Quantification of the basal respiratory capacity in RuvBL1-silenced cells. Values are scaled to the siCTRL average and presented as mean ± SD of at least three independent experiments. Unpaired Student’s *t* test was used for statistical significance. ∗*p* <0.05, ∗∗*p* <0.01, ∗∗∗*p* <0.001, ∗∗∗∗*p* <0.0001. (C) RuvBL1 expression in control and RuvBL1-silenced cells. Representative WB for each cell line, transfected with scramble siRNA, siGAPDH (to monitor transfection efficiency), and siRUVBL1.

**Fig. 3:**
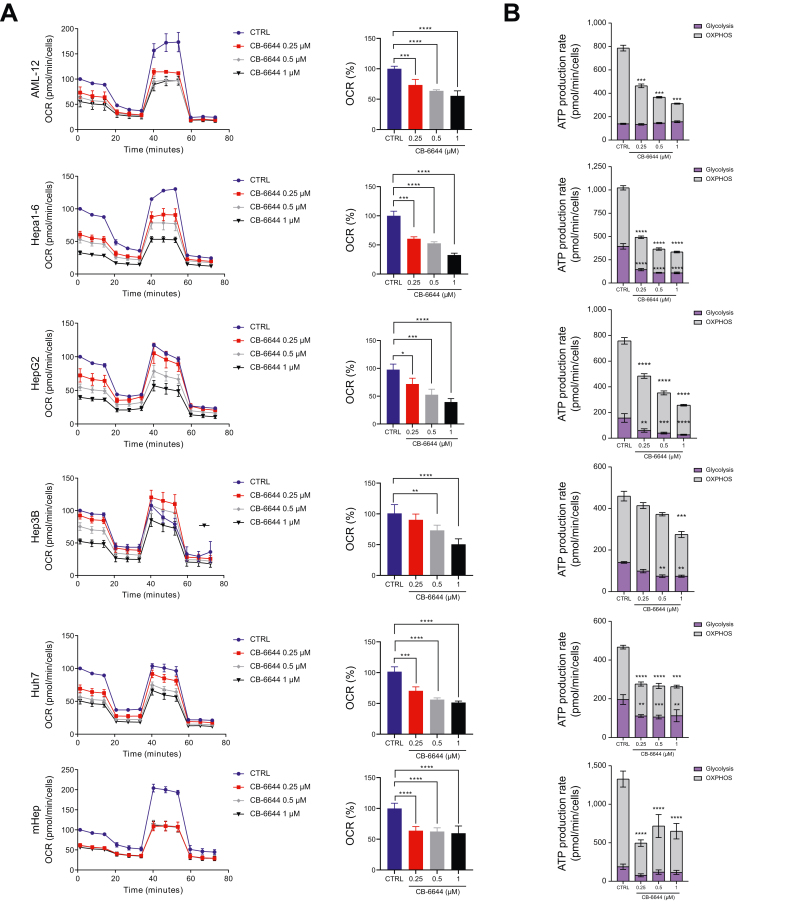
Inhibition of RuvBL1/2 ATPase activity impairs OXPHOS and ATP production. (A) Line graphs: Seahorse MitoStress test profiles of cell lines and primary mouse hepatocytes treated with CB-6644 for 72 h (mean ± SEM, n = 3–5 independent experiments). OCR values are normalised by cell number and scaled relative to the basal OCR of non-treated cells. Bar-graph: Quantification of the basal respiratory capacity shown in panel A (mean ± SD). Statistical significance was calculated by one-way ANOVA with Dunnett’s correction for multiple comparisons. ∗*p* <0.05, ∗∗*p* <0.01, ∗∗∗*p* <0.001, ∗∗∗∗*p* <0.0001. (B) ATP-rate assay showing the relative contribution of glycolysis and OXPHOS to the total ATP production in cells treated with CB-6644 for 72 h (mean ± SEM, *n* = 3). Statistical significance (*vs*. control) was calculated by two-way ANOVA with Dunnett’s correction for multiple comparison. ∗*p* <0.05, ∗∗*p* <0.01, ∗∗∗*p* <0.001, ∗∗∗∗*p* <0.0001. OXPHOS, oxidative phosphorylation.

RuvBL1 was knocked down in murine and human HCC cells (Hepa1-6, HepG2, Hep3B, and Huh7), in a non-tumoural hepatocytic cell line (murine AML-12) and the impact on cellular respiration was measured by the Seahorse MitoStress test (Fig. 2A, B; Fig. S2). Reducing RuvBL1 protein level affected oxidative phosphorylation (OXPHOS) in all tested cell lines, clearly hampering their basal respiratory capacity (Fig. 2; Fig. S2). We next investigated whether the ATPase activity of the RuvBL1/2 complex is required for mitochondrial respiration. The abovementioned cell lines and freshly isolated mouse hepatocytes were treated for 24–72 h with increasing doses of CB-6644 (0.25–1 μM). The basal respiratory capacity was affected in a time- and dose-dependent manner, with higher doses inhibiting OXPHOS after 24 h and lower doses becoming effective after 48 h of treatment (Fig. 3A; Fig. S3A and C).

The reduced OXPHOS in CB-6644-treated cells was mirrored by a significant reduction of the mitochondrial ATP production, which was not compensated for by a concomitant increase in glycolysis, as assessed by the Seahorse ATP-rate assay (Fig. 3B and Fig. S3B and D). Intriguingly, inhibition of RuvBL1/2 activity also reduced ATP production through glycolysis in the cancer cell lines but not in non-transformed AML-12 cells and in primary hepatocytes (Fig. 3B and Fig. S3B and D, purple bars).

We then hypothesised that the reduced respiratory capacity of CB-6644-treated and RuvBL1-silenced cells could stem from a reduction in mitochondrial content or activity, the latter being commonly associated with a reduced mitochondrial membrane potential (ΔΨm).

Treatment with CB-6644 mildly decreases Mitotracker staining intensity, suggesting a reduction in the total mitochondrial mass (Fig. S4A). Although statistically significant, this reduction was relatively modest (5–10% depending on dose and cell line) hardly accounting for the robust loss of the cell respiratory capacity (Fig. 3A). Contrary to our expectation, the ΔΨm indicator JC-1 surprisingly revealed that treatment with CB-6644 induces the hyperpolarisation of mitochondria (increased negative charge across the membrane), a type of disfunction known to precede mitochondrial fragmentation^29^ (Fig. S4B). These data were further corroborated by high-content imaging analysis of AML-12 and Huh7 cells stained with the mitochondrial dye tetramethyl rhodamine methyl ester (TMRM), which accumulates in active mitochondria in a voltage-dependent manner. Consistent with the JC-1 results, CB-6644 dose- and time-dependently increased the average fluorescence intensity of TMRM-stained AML-12 and Huh7 cells, strongly suggesting an increase of the ΔΨm (Fig. S4C and D). Oligomycin inhibits the ATP synthase activity, preventing proton re-entry through complex V and typically inducing a transient hyperpolarisation in coupled mitochondria. As expected, oligomycin treatment caused a marked increase in ΔΨm in vehicle-treated AML-12 cells (Fig. S4E). In contrast, CB-6644–treated cells exhibited an elevated basal ΔΨm and showed little to no further increase upon oligomycin addition, indicating a reduced oligomycin-sensitive proton flux through ATP synthase. Together, these results indicate that targeting RuvBL1 reduces mitochondrial oxidative phosphorylation, which is accompanied by mitochondrial hyperpolarisation and a decrease in mitochondrial mass.

### RuvBL1 is localised to mitochondria, and it is required for their integrity

**Fig. 4:**
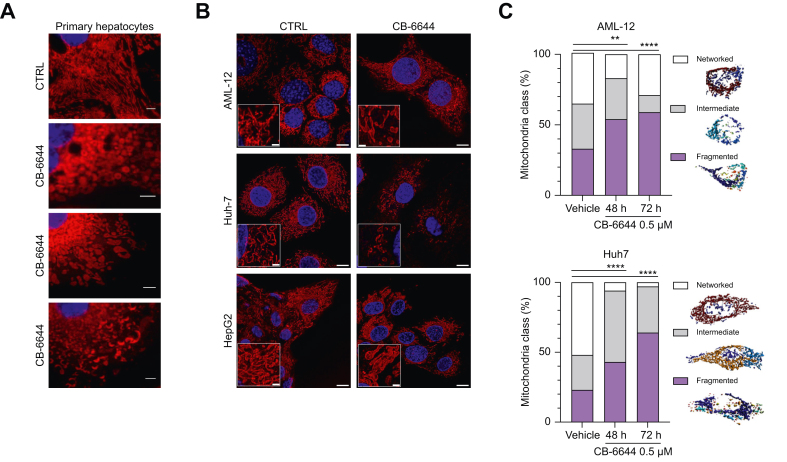
CB-6644 induces mitochondrial swelling and fragmentation. (A) Primary mouse hepatocytes treated with 0.5 μM CB-6644 for 48 h. Widefield images, scale bar = 5 μm. (B) Cell lines treated with 0.5 μM CB-6644 for 48 h. Confocal images, scale bar = 10 μm. Inset scale bar = 2 μm. (C) Classification of the mitochondrial network structure in AML12 and Huh7 exposed to 0.5 μM CB-6644 for 48 h. Cells were immunostained for the mitochondrial marker TOMM20 and imaged by high-content confocal imaging. Representative isosurface rendering are also shown (right panel). Isosurface rendering was false-coloured to represent the volume of each mitochondrial particle. Statistical significance (*vs*. CTRL) was calculated by Fisher’s exact test: ∗*p* <0.05, ∗∗*p* <0.01, ∗∗∗*p* <0.001, and ∗∗∗∗*p* <0.0001.

Staining with Mitotracker and TMRM clearly revealed that CB-6644 treatment alters mitochondrial morphology. Fig. 4 shows the heterogeneity of mitochondrial shapes observed in primary mouse hepatocytes and cell lines treated with 0.5 μM CB-6644 for 48 h. Although mitochondria are elongated and uniformly shaped in vehicle-treated cells, they become swollen or fragmented in CB-6644-treated cells. Quantitative assessment of the mitochondrial network integrity, performed by high-content imaging in TOMM20-stained AML-12 and Huh7 cell lines, confirmed that inhibition of RuvBL1/2 ATPase activity results in the progressive fragmentation of the mitochondrial network (Fig. 4C).

**Fig. 5:**
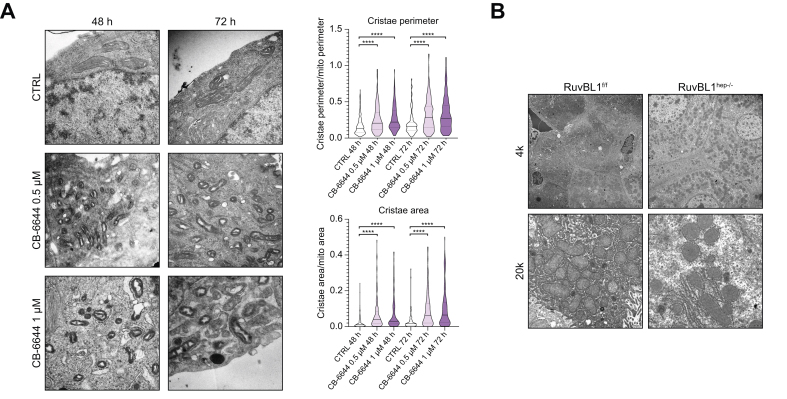
CB-6644 disrupts mitochondrial cristae structure. (A) TEM morphometric analysis of mitochondrial cristae in Huh7 cells treated with 0.5–1 μM CB-6644 for 48–72 h. Statistical significance (*vs*. time-matched CTRL) was calculated by Kruskal–Wallis with Dunn’s correction for multiple comparison. ∗*p* <0.05, ∗∗*p* <0.01, ∗∗∗*p* <0.001, and ∗∗∗∗*p* <0.0001. (B) Liver samples of RuvBL1^f/f^ and RuvBL1^hep-/-^ mice imaged by TEM at 4,000 × and 20,000 × magnification.

Assessment of the mitochondrial structure by transmission electron microscopy (TEM) revealed that even the lower dose of CB-6644 (0.25 μM) impacts mitochondrial morphology after 48 h in all the tested cell lines, causing the loss of matrix electron density and the disruption of mitochondrial cristae (Fig. S5). We further investigated this intriguing aspect by a detailed morphometric analysis of Huh7 mitochondria by TEM. As shown in Fig. 5, treatment with CB-6644 induces the swelling of mitochondrial cristae in a dose- and time-dependent manner.

Notably, a similar phenotype was observed in liver samples from RuvBL1^hep-/-^ mice, whose hepatocytes show reduced mitochondrial density and strikingly fewer mitochondrial cristae, compared to RuvBL1^f/f^ control mice (Fig. 5B). Notably, RuvBL1^hep-/-^ mice, but not RuvBL1^f/f^ or RuvBL1^hep+/-^ ones,^12^ present hepatocellular damage, high apoptosis and proliferation indexes, suggestive of chronic liver damage and regeneration (manuscript in preparation).

**Fig. 6:**
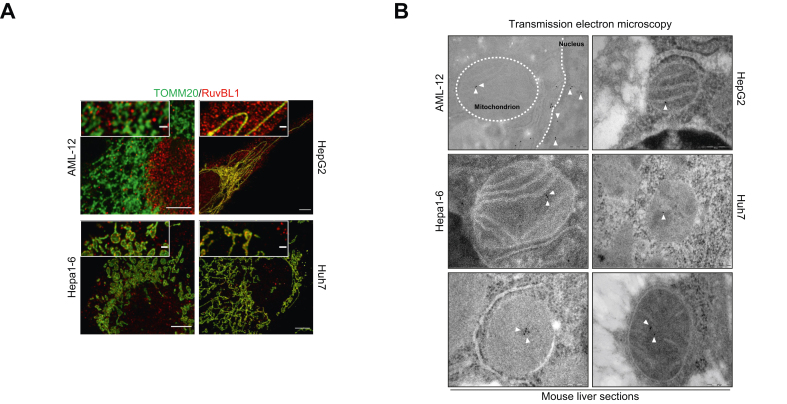
RuvBL1 is partially localised to mitochondria. (A) Deconvoluted super-resolution STED images of RuvBL1 (red) and TOMM20 (green). Images are presented as maximum intensity projection of z-stacks. Scale bar = 5 μm. Insets: enlarged details with scale bar = 1 um. (B) Immunogold TEM showing RuvBL1 localisation within mitochondria (arrowheads). TEM, transmission electron microscopy.

The evident *in vitro* and *in vivo* mitochondrial phenotype induced by RuvBL1 targeting prompted us to investigate a yet unreported mitochondrial localisation of RuvBL1. Indeed, super-resolution STED microscopy imaging strongly suggested that RuvBL1 localises in proximity and within mitochondria in the tested cell lines (Fig. 6A). Immunogold labelling and TEM consolidated the mitochondrial localisation of RuvBL1 in all tested cell lines as well as in murine liver sections (Fig. 6B). Furthermore, cellular fractionation experiments confirmed the enrichment of RuvBL1 in mitochondria purified by ultracentrifugation (Fig. S6A).

### Inhibition of RuvBL1/2 activity affects the mitochondrial proteome and impairs ATP synthase activity

**Fig. 7:**
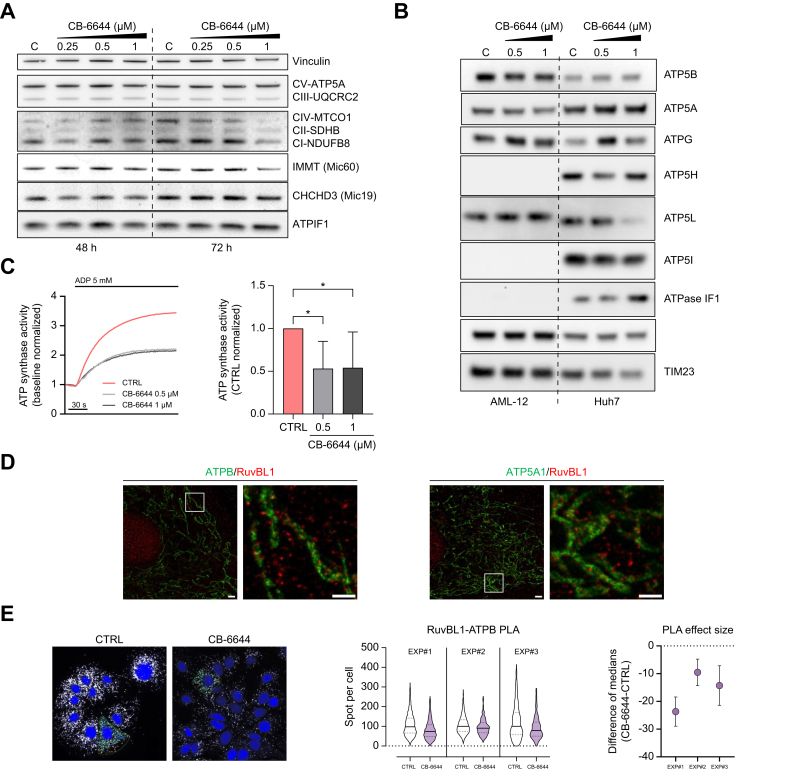
CB-6644 impairs complex V activity without affecting its assembly. WB of ETC complex subunits, MICOS components, and APTIF1 in CB-6644-treated Huh7 cells. (B) WB of complex V subunits in AML-12 and Huh7 cells treated with CB-6644 for 48 h. (C) ATP synthase activity in Huh7 exposed to 0.5–1 μM CB-6644 for 72 h Huh7 cells expressing mitochondrially targeted firefly luciferase were permeabilised with digitonin, then mitochondria were energised with glutamate and malate. ATP synthesis was stimulated by the administration of 5 mM ADP. Statistical significance was calculated by one-way ANOVA with Dunnett’s correction for multiple comparisons (mean ± SD, n = 3). ∗*p* <0.05. (D) STED microscopy of RuvBL1 and (F_1_)-ATP synthase subunits in Huh7 cells. Scale bar = 2 μm for the larger field and = 1 μm for the enlarged detail. (E) Representative ATPB-RuvBL1 PLA images in Huh7 cells, showing cell segmentation with ScanR. The graphs show the distribution of PLA spot (count per cell) in CTRL and CB-6644-treated cell across three replicate experiments and the relative effect size with 95% CI (EXP#1: n = 1,500; EXP#2 n = 2,445; EXP#3 n = 901).

We next focused on the potential mechanisms underlying the disruption of mitochondrial respiration and cristae structure after CB-6644 treatment. We first assessed the integrity of the electron transport chain (ETC) by analysing the expression of key subunits that are degraded upon disassembly of the ETC complexes. Treatment with CB-6644 for 72 h caused a dose–response reduction of MTCO1 (complex IV) and NDUFB8 (complex I) subunits in Huh7 cells, suggesting a detrimental effect on the stability of these ETC complexes (Fig. 7A).

The mitochondrial cristae structure is shaped and stabilised by several factors, including the ATP synthase complex at the cristae tips and the MICOS complex and OPA1 tethering at the cristae junctions.^30^^,^^31^

The expression of MICOS complex subunits Mic60 (IMMT) and Mic19 (CHCHD3) was reduced after 72 h of treatment with CB-6644 in Huh7 cells (Fig. 7A). Full-length OPA1 protein is processed by cellular proteases Oma1 and Yme1l1 to produce several long and short forms, which act as key regulators of the inner mitochondrial membrane (IMM) dynamics during fission/fusion events and cristae remodelling.^32^ Consistent with the change in mitochondria morphology, treatment of Huh7 cells with CB-6644 resulted in a shift in the abundance of OPA1 fragments with a relative increase of the short forms, a pattern partially overlapping with that generated by oligomycin, which has been shown to induce OPA1 cleavage and mitochondrial fragmentation^33^ (Fig. S6B).

We reasoned that the disruption of the mitochondrial structure caused by CB-6644 would be mirrored by informative modifications of the mitochondrial proteome. To rapidly isolate mitochondria from control and CB-6644-treated cells for subsequent MS analysis, we generated stable Huh7 clones expressing HA-OMP25-EGFP for IP, and FLAG-OMP25-EGFP as negative controls.^34^

Quantitative IP/MS analysis of the mitochondrial proteome identified eight proteins consistently present in control samples only (*bona fide* downregulated by CB-6644), 13 proteins consistently identified only in treated samples (*bona fide* upregulated by CB-6644), and five proteins which were identified in all samples, but whose expression was significantly upregulated in CB-6644-treated samples (Fig. S7).

Interestingly, among these modulated proteins, several are related to pyruvate metabolism and TCA (PDP2, D2HGDH, OGDHL, HAGH), lipid metabolism (MECR, AMACR, MLYCD), mitochondrial translation (MRPL52, ALKBH1), iron-sulphur cluster assembly and electron transport (ISCA2, IBA57, CISD3, COQ8B, PPTC7), leucine degradation and ketogenesis (IVD, HMGCL), and to membrane transporters involved in the maintenance of the mitochondrial membrane potential (SLC35F6, MCUR1, ATP synthase mitochondrial F1 complex Assembly Factor 2 [ATPAF2]). All these processes and functions appear to be consistent with the metabolic data and mitochondrial phenotype described so far.

ATPAF2 drew our attention as its upregulation by CB-6644 is suggestive of a RuvBL1/2-mediated mechanism that converges on complex V. We first validated the proteomic data by western blotting (WB), confirming that CB-6644 treatment upregulates ATPAF2 protein level in immunoprecipitated mitochondria of Huh7 cells (Fig. S6C). Next, we evaluated the expression of specific ATP synthase subunits essential for the function and stability of the complex, particularly α and β subunits (F1, catalytic portion), γ subunit (central stalk), and d, g, and e subunits (peripheral stalk) (Fig. 7B). Intriguingly, several ATP synthase subunits were upregulated by CB-6644, paralleling ATPAF2 expression and in line with ATP5A data obtained from the total cell lysates (Fig. 7A). Collectively, these data suggest that complex V assembly is not impaired by CB-6644. Nevertheless, the mitochondrial ATP production, measured in digitonin-permeabilised Huh7 under fuelled TCA cycle and in the presence of exogenous ADP, was clearly reduced by CB-6644 (Fig. 7C; Fig. S6D). Interestingly, the expression of ATPIF1, the main inhibitor of complex V activity, was found upregulated by CB-6644 in a dose-dependent manner (Fig. 7A, B).

Wondering how RuvBL1/2 inhibition could result in reduced ATP synthase activity, we looked for potential interaction between these two complexes. Super-resolution STED and STORM microscopy, performed with different antibody pairs, clearly localises RuvBL1 in proximity with (F_1_)-ATP synthase (alpha and beta subunits, Fig. 7D; Fig. S6E). Proximity ligation assay (PLA), which detects proteins interacting within a 40 nm range, confirmed the association of RuvBL1 with the (F_1_)-ATP synthase complex (Fig. 7E). Quantification of the PLA signal by high-content imaging microscopy revealed that CB-6644 induces a moderate but consistent reduction of the RuvBL1-ATPB interaction (Fig. 7E). Taken together, these data demonstrate that inhibition of RuvBL1/2 affects complex V ATP synthase activity by possibly disrupting the interaction between these two complexes, leading to mitochondrial membrane hyperpolarisation and, eventually, to mitochondrial damage.

### *In-silico* analysis highlights a significant correlation between mitochondrial-related processes and RUVBL1 expression in human HCC

**Fig. 8:**
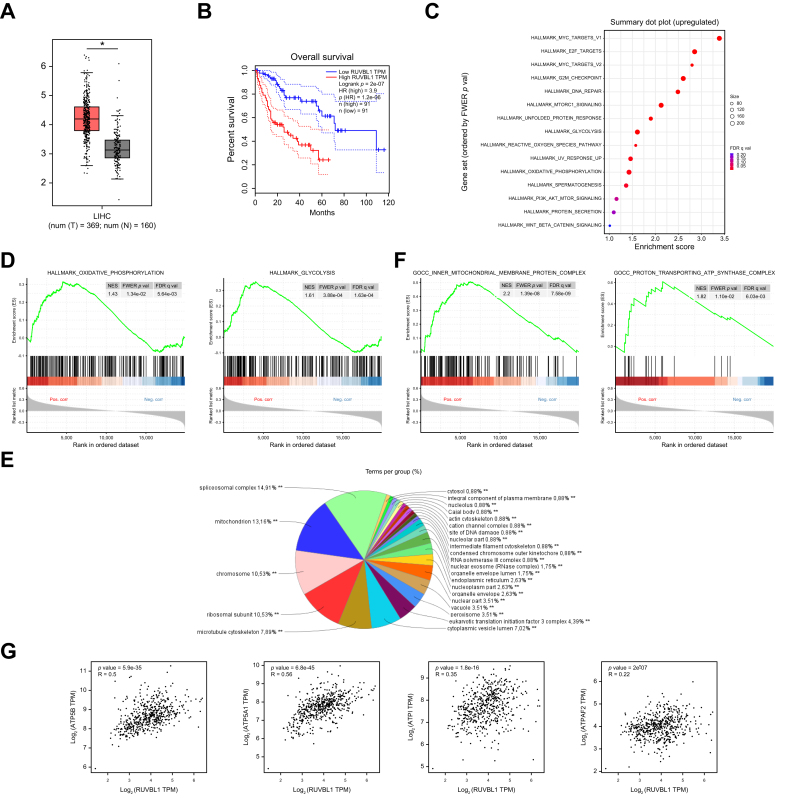
RuvBL1 expression correlates with mitochondrial functions in human HCC. (A) RuvBL1 expression in HCC and non-tumoural liver samples (TCGA-LIHC and GTEX cohorts, performed with GEPIA2). (B) Overall survival of patients with HCC with high-*vs*. low-RuvBL1 expression (quartile cut-off, TCGA-LIHC cohort, performed with GEPIA2). (C) Enrichment analysis of high-RuvBL1 expressing HCC in the LIHC cohort *vs*. HALLMARK gene sets (performed with GENI). (D) GSEA profile of selected HALLMARK gene sets (performed with GENI). (E) Gene Ontology analysis of DEGs in high-*vs*. low-LIHC (cut-off z-score ± 2, performed with ClueGO). (F) GSEA of cellular components in high-RuvBL1 expressing LIHC (performed with GENI). (G) Spearman’s correlation analysis of RUVBL1 and (F_1_)-ATP synthase subunits, ATPIF1 and ATPAF2 in liver samples (TCGA-LIHC + GTEx, performed with GEPIA2). DEGs, differentially expressed genes; GSEA, Gene Set Enrichment Analysis; HCC, hepatocellular carcinoma; LIHC, liver hepatocellular carcinoma; TCGA, The Cancer Genome Atlas.

Finally, we explored the relevance of our findings to human HCC, leveraging the publicly available TCGA and GTEx databases. RUVBL1 is overexpressed in the TCGA-LIHC cohort compared with matched TCGA and GTEx normal liver samples, confirming a previous report,^11^ and its higher expression correlates with a more advanced stage and a worse prognosis^12^ (Fig. 8A, B; Fig. S8A). Indeed, in the TCGA-LIHC cohort, RUVBL1 scores within the top 10 genes predicting overall survival (Fig. S8B). Gene Set Enrichment Analysis (GSEA) of RUVBL1-correlated genes in the TCGA-LIHC cohort was performed through the GENI webapp.^35^ The top-scoring GSEA signatures positively correlated with RUVBL1 expression are shown in Fig. 8C, among which MYC, E2F, and mTORC1 signalling are well-known functions regulated by RUVBL1.^12^^,^^14^^,^^19^^,^^36^ Indeed, the inhibitory phosphorylation of mTOR at serine 2448^37^ is significantly associated with reduced RUVBL1 expression in the LIHC cohort (Fig. S8C), supporting a key metabolic role of this ATPase in human HCC. Strikingly, glycolysis and oxidative phosphorylation are also significantly enriched with RUVBL1 expression in the LIHC dataset (Fig. 8D). These data are further corroborated by the GO analysis of differentially expressed genes (DEGs), which identifies the mitochondrial compartment as a significantly enriched cellular component in HCC with high RUVBL1 expression (Fig. 8E). Finally, in human liver samples from the TCGA and GTEx databases, RUVBL1 shows a very strong correlation with (F1)-ATP synthase subunits and, to a lesser extent, with ATPIF1 and ATPAF2 (Fig. 8G).

Taken together, these findings strongly suggest that RUVBL1 expression in human HCC is associated with mitochondrial metabolic processes, including oxidative phosphorylation, and that these functions likely contribute to the increased aggressiveness of tumours with higher RUVBL1 expression.

## Discussion

RuvBLs proteins are drawing increasing attention for their pleiotropic role in many key cellular processes, including DNA repair, chromatin remodelling, ribosome biogenesis, and mTOR pathway regulation. Despite a growing body of reports highlighting the connections of RuvBLs with metabolic functions,^12^^,^^14^^,^^19^^,^^20^ the impact of RuvBL1 targeting on cell metabolism is still poorly understood.

Here, we present clear evidence for the role of RuvBL1 in maintaining mitochondrial integrity and metabolic functions.

We found that inhibiting RuvBL1/2 ATPase activity in Huh7 HCC cells strongly affects amino acid metabolism and ATP production from glycolysis and the TCA cycle (Fig. 1, Fig. 3; Figs. S1 and S3, Table S1). It is interesting to note that aspartate and asparagine are the only amino acids whose levels are clearly increased by CB-6644. Aspartate and asparagine largely share the same metabolic pathways at the crossroad of TCA and urea cycles. Aspartate aminotransferase (AST) catalyses the reversible conversion of glutamate to aspartate concomitantly generating alpha-ketoglutarate from oxalacetate.^38^^,^^39^ Therefore, aspartate levels are key both for the malate–aspartate shuttle (which imports glycolysis-produced NADH into mitochondria) and for the anaplerotic reactions that, through amino acid catabolism in the urea cycle, replenish the TCA cycle intermediates. Indeed, the urea cycle is among the top-scoring pathways emerging from the metabolomic analysis of CB-6644-treated cells (Fig. 1C; Fig. S1B, Table S1). We previously observed that RuvBL1^hep+/-^ mice have reduced levels of AST (but not ALT/GPT) compared with wild-type mice^12^ and, interestingly, RUVBL1 and AST (but not ALT/GPT) are found positively correlated in human liver samples from the GTEx and TCGA cohorts (Fig. S9A, B). In amino acid-deprived cells, asparagine and aspartate serve as key exchange factors for other amino acids, in particular arginine and serine, which act as mTORC1 activators.^40^ RUVBL1 and asparagine synthetase show a very strong positive correlation in human liver samples from the TCGA and GTEX databases (Fig. S9C), in line with the role of RuvBL1 in supporting mTORC1 activity. along this line of thought, asparagine and aspartate levels may increase in RuvBL1-targeted cells as a compensatory mechanism for reduced mTORC1 activity.

Among the amino acids whose levels decrease upon RuvBL1/2 inhibition, leucine, isoleucine, and valine are of particular interest. Under low glucose conditions, these branched-chain amino acids are degraded to produce ketone bodies, which supply the carbon backbone for TCA cycling. In the context of cells maintained in complete medium, the activation of ketogenesis may appear counterintuitive. However, several clues support this interpretation; first, CB-6644 induces a significant upregulation of mitochondrial IVD and HMGHCL, two key enzymes of leucine catabolism and ketogenesis (Fig. S7B).[41], [42], [43] Second, CB-6644 increases the expression of mitochondrial D2HGDH (Fig. S7A), which catalyses the conversion of D-2-hydroxyglutarate (2-DHG) to alpha-ketoglutarate to support the TCA cycle. 2-DHG can be produced by the 4-hydroxybutyrate catabolism,^44^ oncogenic isocitrate dehydrogenase (IDH), and propionyl-CoA shunting into the TCA cycle.^45^^,^^46^ Propionyl-CoA is a central intermediate metabolite of isoleucine and valine degradation^47^ and can also be formed by odd-chain fatty acids, cholesterol, C-5 ketone bodies, threonine, and methionine.^48^ Within the LIHC and GTEx databases, RUVBL1 expression shows a very strong negative correlation with the gene signature of propionate shunting (Fig. S9D).^49^ Intriguingly, while ketone bodies produced by the liver are normally utilised by extrahepatic tissues, under nutrient deprivation stress, HCC cells have been recently shown to reactivate OXCT1 expression and ketolysis as a metabolic adaptation for energy supply.^50^^,^^51^

Taken together, these data show that the inhibition of RuvBL1/2 ATPase activity has a noticeable impact on glycolysis and on the TCA cycle, and suggests that mitochondrial metabolism is rewired towards anaplerosis, likely through amino acid degradation and ketogenesis.

Direct measurement of mitochondrial activity by Seahorse analysis confirmed that targeting RuvBL1 by RNAi (Fig. 2; Fig. S2), or CB-6644 treatment (Fig. 3; Fig. S3), reduces mitochondrial respiration and ATP production from OXPHOS. Remarkably, glycolytic ATP production was reduced by CB-6644 in all the tested cancer cell lines but not in non-transformed AML-12 cells and in primary mouse hepatocytes. Besides the altered metabolic flux described above, we collected several pieces of evidence supporting an impairment of ATP synthase activity, which may contribute to the reduced mitochondrial ATP production. In fact, CB-6644 induces an unexpected hyperpolarisation of the ΔΨm, as shown by JC-1 and TMRM staining (Fig. 4B–D). These observations are confirmed by direct measurement of the ΔΨm in CB-6644-treated AML-12 cells, which is higher than in control cells and refractory to further increase by the complex V inhibitor oligomycin (Fig. 4E). Interestingly, ATPAF2, a key assembly factor for complex V,^52^ was among the few proteins identified by MS as significantly upregulated by CB-6644 (Figs. S6C and S7B), which prompted us to investigate ATP synthase in more detail. Analysis of ETC complexes in whole cells and in immunoprecipitated mitochondria of Huh7 cells failed to reveal a consistent downregulation of ATP synthase subunits (Fig. 7A, B), suggesting that the assembly of complex V is not impaired. Nevertheless, CB-6644 dose-dependently increases ATPIF1, the main inhibitory factor of complex V (Fig. 7A, B),^53^ and reduces ATP synthase activity even when measured in energised mitochondria (Fig. 7C).

It is conceivable that the increased expression of ATPAF2 and ATPIF1 may occur as an adaptive feedback mechanism to the impaired ATP synthase activity aimed at restoring its function and at preventing the reverse mode of action of complex V, thereby avoiding further ATP disposal, stabilising complex V dimerisation despite loss of cristae, and promoting cell survival.^53^ These molecular clues are paralleled by evident alteration of the mitochondrial morphology, reduced network integrity and disarrangement of the cristae in CB-6644 treated cells (Fig. 4, Fig. 5; Fig. S5). Of notice, the genetic deletion of RuvBL1 in mature hepatocytes (RuvBL1^hep-/-^ mice) recapitulates this phenotype *in vivo*, made evident by the reduced mitochondrial density and scarcity of cristae (Fig. 5B).

The altered cristae morphology is accompanied by an increase in short OPA1 isoforms (Fig. S6B) and a reduction in MICOS complex components (Fig. 7A). OPA1 and MICOS complex, together with complex V dimerisation, play a key role in the shaping of the IMM at the junctional and tip sides of the mitochondrial cristae^31^^,^^54^ and are crucial for isolating the cristae lumen from the intermembrane space, thus contributing to the generation of the proton gradient driving the ΔΨm. The unambiguous localisation of RuvBL1 within the mitochondrion (Fig. 6; Figs S6A and S7D, E), to our knowledge previously unreported, strongly suggests that this protein may directly participate in processes essential for the structural and functional integrity of this organelle. This line of thought is supported by super-resolution imaging and PLA localising RuvBL1 within 40 nm of (F1)-ATP synthase in Huh7 cells (Fig. 7D, E; Fig. S6E). The small but consistent reduction of RuvBL1-ATPB proximity caused by CB-6644 (Fig. 7E) suggests that this interaction may be required for proper complex V function and warrants further mechanistic investigation. Nevertheless, the emerging picture clearly depicts RuvBL1 as a key element supporting mitochondrial metabolism, structure, and function. In human HCC samples from the TCGA-LIHC cohort, higher RuvBL1 expression correlates with more advanced stage (Fig. S8A) and RUVBL1 scores within the 10 top genes correlating with reduced survival (Fig. 8A; Fig. S8B). RUVBL1 correlates with glycolysis and oxidative phosphorylation in GSEA (Fig. 8C, D), which agrees with its role in supporting mTORC1 signalling (Fig. 8C; Fig. S8C).^37^ Consistent with our metabolomic results, RUVBL1 also negatively correlates with branched-chain amino acids degradation and propanoate metabolism (Fig. S8D). Interestingly, a prominent proportion of genes differentially expressed between LIHC samples with high-*vs* low-RuvBL1 expression are annotated with the term ‘mitochondrion’ in GO cellular component analysis (Fig. 8E). Indeed, the IMM and the ATP synthase complex are among the cellular components terms significantly enriched for RUVBL1 in the LIHC cohort (Fig. 8F), and RUVBL1 gene expression shows a strong positive correlation with (F_1_)-ATP synthase components and regulators in human liver samples from the TCGA and GTEx database (Fig. 8G). It is therefore tempting to speculate that, among the several potential tumour-promoting functions of RuvBL1,^3^ supporting the mitochondrial metabolic processes and complex V activity are key to human HCC. As RuvBL1 overexpression and mitochondrial metabolic rewiring are shared features across several cancer types, future studies will need to assess the relevance of mitochondrial RuvBL1 in context beyond HCC.

In conclusion, we uncovered a novel metabolic function and cellular localisation of the AAA+ ATPase RuvBL1, which interacts with ATP synthase and emerges as a central regulator of mitochondrial activity. As RuvBL1 plays multiple roles in human pathophysiology, including cancer, further research is warranted to exploit its mitochondrial localisation and related functions as potential therapeutic targets.

## Abbreviations

2-DHG, D-2-hydroxyglutarate; AST, aspartate aminotransferase; ATPAF2, ATP synthase mitochondrial F1 complex Assembly Factor 2; DEGs, differentially expressed genes; ETC, electron transport chain; GC-MS, gas chromatography-mass spectroscopy; GO, Gene Ontology; GSEA, Gene Set Enrichment Analysis; HCC, hepatocellular carcinoma; IB, intracellular buffer; IBluc, IB containing 25 μM luciferin; IDH, isocitrate dehydrogenase; IMM, inner mitochondrial membrane; LIHC, liver hepatocellular carcinoma; OXPHOS, oxidative phosphorylation; PLA, proximity ligation assay; TCGA, The Cancer Genome Atlas; TMRM, tetramethyl rhodamine methyl ester, OPA1, Optic Atrophy 1, OCR, oxygen comsumption rate.

## Authors’ contributions

Conceptualization: TM. Project administration: TM, AG. Investigation: TM, IS, AG, DP, FB, AS, DG, PN, SP, EC, AC, MB. Formal analysis: TM, IS, AG, DP, FB, AS, DG, SP, MB. Resources: ML, OB, PP, MB. Methodology: PP, MB. Validation: PP, MB. Supervision: AG. Funding acquisition: AG. Writing – original draft, review & editing: TM. Writing – review & editing: AG, DP, FB, PP, MB, AG.

## Data availability

The data that support the findings of this study are available from the corresponding author upon reasonable request. The mass spectrometry proteomics data have been deposited to the ProteomeXchange Consortium via the PRIDE^55^ partner repository with the dataset identifier PXD075574 and 10.6019/PXD075574.

## Financial support

This work was supported by the 10.13039/501100005010Italian Association for Cancer Research (AIRC) with grants IG-20590 (to AG) and IG-23670 (to PP). The study was also supported by Progetti di Rilevante Interesse Nazionale (PRIN20227Z2XRB to MB; 2020RRJP5L, 202259LHXM, P2022WY85 K_001, and PNRR-CN00000041 to PP), by the 10.13039/501100003196Italian Ministry of Health - 10.13039/501100009888Tuscany Region with grant GR-1600315 awarded to TM, and by the Multi-User Equipment Program by AIRC and 10.13039/501100015694Fondazione Cassa di Risparmio di Firenze for the Molecular Medicine Facility of the Department of Clinical and Experimental Biomedical Sciences “Mario Serio”, 10.13039/501100004434University of Florence.

## Conflicts of interest

The authors declare no conflicts of interest.

Please refer to the accompanying ICMJE disclosure forms for further details.
