# Supplementary material for: Targeting RuvBL1 disrupts mitochondrial metabolism and structure in hepatocellular carcinoma
Source: JHEP Rep. 2026 Apr 17;8(7):101858. doi: 10.1016/j.jhepr.2026.101858 (PMC13310627; doi:10.1016/j.jhepr.2026.101858)
Supplement: Multimedia component 1 [file mmc1.pdf]

Fig. S8

Targeting RuvBL1 disrupts mitochondrial metabolism and structure in hepatocellular carcinoma

Tommaso Mello, Irene Simeone, Alice Guida, Dimitri Papini, Francesca Begnozzi, Alice Santi, Daniele Guasti, Patrizia Nardini, Simone Polvani, Matteo Lulli, Oxana Bereshchenko, Elisabetta Ceni, Armando Curto, Paolo Pinton, Massimo Bonora, Andrea Galli

Table of contents

Materials and methods……………………………………………………………………….2

Supplementary figure legends…………………………………………………………..……14

Supplementary figures………………………………………………………….............……18

Table S1…………………………………………………………………………………...27

Supplementary statistics…………………………………………………………separate excel file

Supplementary references………………………………………………………………...….29

Materials and methods

Primary hepatocytes isolation

All procedures involving laboratory animals were conducted in accordance with institutional ethical norms and national laws, following approval from the Italian Ministry of Health (D.No. 30/2013 and D.No. 665/2018). Hepatocytes were isolated from 3 months old C57/BL6 mice by collagenase-dispase perfusion through the portal vein. Livers were perfused in situ with 45 ml of Gibco Liver Perfusion Media (Invitrogen. Carlsbad, CA) followed by 45 ml of Gibco Liver Digestion Media (ThermoFisher Scientific, Italy). The liver was excised, minced, and strained through 100µM and 70µM EASYstrainers (Greiner BIO-ONE). The dispersed hepatocytes were collected by centrifugation at 50g for 2 minutes at 4°C. Hepatocytes were separated by gradient centrifugation in 40% Percoll (Sigma-Aldrich) at 200g for 10 minutes at 4°C and washed twice with pre-cooled plating media (MEM containing 5% FBS and supplemented with GlutaMAX (Gibco), penicillin and streptomycin (Sigma Aldrich). The hepatocyte pellet was then resuspended with plating media, counted and viability was assessed by Trypan Blue exclusion. Typical yield was around $6*10^6$ hepatocytes per liver with >95% viability. Primary hepatocytes were plated in collagen coated plates (Sigma-Aldrich C8919, 10ug/cm^2). After 2.5h, the medium was changed to HepatoZYME-SFM (Gibco) supplemented with Pen/Strep, GlutaMAX and collagen I (1.25ug/cm^2) for sandwich culture.

Cell culture

Authenticated cell lines were obtained from suppliers reported in the supplementary CTAT table. Upon arrival, cell lines were amplified for 3 to 4 passages, tested for mycoplasma (Sigma-Aldrich), aliquoted and stored in liquid nitrogen. Thawed cells were passed twice to ensure full recovery and used within passage 20. All cell lines were cultured in media supplemented with 10% FBS, stable glutamine (GlutaMAX, Gibco) and without antibiotics. Mycoplasma was tested regularly by PCR. Hepa1-6 and Huh7 were maintained in DMEM, HepG2 and Hep3B in MEM, AML-12 in DMEM/F12 supplemented with Selenium, Transferrin, Insulin, Hepes and Dexamethasone.

Silencing was performed using siRNA (iBONI siRNA, Riboxx GmbH) to a final concentration of 20nM in all cell lines. Sequence details are reported in the Supplementary CTAT Table.

Generation of cells with epitope-tagged mitochondria

pMXs-3XHA-EGFP-OMP25 and pMXs-3XMyc-EGFP-OMP25 constructs were purified using the PureYield™ Plasmid Miniprep kit. 1.5 µg of DNA was transfected into Huh7 cells using FuGENE HD at a 4.5:1 lipid:DNA ratio. 48 hours post-transfection, cells were selected with 150 µg/mL Blasticidin and then FACS-sorted for EGFP signal. pMXs-3XHA-EGFP-OMP25 (Addgene plasmid #83355) and pMXs-3XMyc-EGFP-OMP25 (Addgene plasmid #83356) were a gift from David Sabatini[1].

Gene expression analysis

RNA was extracted from Huh7, Hep3B and HepG2 cell lines using the RNeasy mini kit (Qiagen) and complementary DNA was synthesized by the PrimeScript RT Reagent Kit (Promega) according to the manufacturer's protocol. qPCR was performed using 20ng of retrotranscribed RNA per reaction and qPCR validated primers (Qiagen). Gene expression was quantified by qPCR using the ΔΔCt method and β-2 microglobulin as a reference gene. Master mix was Luna Universal qPCR Master Mix (New England Biolabs), thermal cycler was Rotor- Gene Q (Qiagen), and analysis was run on Q-Rex Software (Qiagen). P values were calculated from the ΔCt distributions using Student's t-test.

Seahorse Analysis

The day before transfection, cells were plated in 24 well plates to reach 40-50% confluence in 24h (AML-12, Hepa1-6, Huh-7 and Hep3B: 25.000cells/well; HepG2: 40.000cells/well). Gene silencing was performed with 20nM IBONI siRNA (Riboxx GmBH) or 5nM Silencer Select validated siRNA (Thermo Fisher), using negative-control siRNA and GAPDH siRNA to evaluate silencing efficiency and transfection efficiency, respectively. INTERFERin (Polyplus) or RNAiMAX (Invitrogen) were used as transfection reagents. After 48h, silenced cells were trypsinized and plated in the Seahorse XFe 96 well plate (HepG2, Huh7: 10.000 cells/well; Hep3B, AML-12, Hepa1-6: 8000cells/well). The

following day (72h after siRNA transfection) the Seahorse MitoStress test was performed following the manufacturer protocol. Immediately after the assay, cells were fixed with 4% PFA, then stained with 1uM TO-PRO-3 Iodide (Life technologies) for 30' to label the nuclei and imaged with a Typhoon Scanner equipped with a 633nm laser line a Cy5 filter set (670nm, bandpass) (GE Healthcare). The integrated density of TO-PRO-3 in each well was quantified with Fiji/ImageJ[2] and used to normalize OCR values.

For OCR evaluation after CB-6644 treatment, cells were seeded directly in the Seahorse XFe 96well plate (HepG2, Hep3B, Huh-7: 5000cells/well; AML-12, Hepa1-6: 3000cells/well). 6 hours after seeding, cells were treated with CB-6644 diluted in 20ul of complete culture media for 24h to 72h. MitoStress test (Agilent) and Real Time ATP Rate Assay (Agilent) analysis were performed following the manufacturer protocol, and OCR values were normalized by TO-PRO-3 iodide signal intensity as described above.

Mouse primary hepatocytes were plated in Seahorse XFe 96-well plates, 4000cells/well in 80uL/well of Hepatocyte Plating Medium (Gibco) pre-coated with 10ug/cm^2 of collagen (Sigma-Aldrich). After 2.5h, the plating medium was carefully removed, and primary hepatocytes were sandwiched with collagen (1,25ug/cm^2) diluted in 80ul/well of HepatoZYME-Serum Free Medium (Gibco) containing Pen/Strep (Sigma-Aldrich) and Glutamax (Gibco). The following day the cells were treated with CB-6644 diluted in 20uL/well of HepatoZYME-SFM containing antibiotics and glutamine.

Metabolomic analysis

Huh7 cells were plated in 6-well plates (300.000cells/well) in 3 ml of complete medium (DMEM high glucose, 10% FBS, glutamine) and treated with 0.5 µM of CB6644 or vehicle alone. After 48h, each well was washed twice with cold saline solution (0.9% NaCl) and cells were scraped in 400 µl of 80% cold methanol supplemented with 1 µg/ml of norvaline (Merck, 53721), used as an internal standard. The cell suspension was sonicated 3 times for 5 seconds on ice using a pulse sonicator (Bandelin Sonopuls HD2070, 40% pulse time, 50% power output) and then centrifuged for 15 min at 14.000 x g. The supernatant was stored at -80°C until processed, while the pellet was dissolved with

100 µl of 200 mM NaOH for 15 min at 95°C and used to determine protein concentration by the BCA method. The supernatant was dried using a vacuum concentrator (Labconco). Dried extracts were derivatized in 10 µl of 40 mg/mL methoxamine hydrochloride (Merck, 226904) in pyridine (Merck, 270970) at 37 ◦C for 90 min, followed by 50 µl of MTBSTFA (Merck, 375934) at 60 °C for 30 min. Data acquisition was performed by using an Intuvo 9000 GC/5977B MS System (Agilent Technologies) equipped with an HP-5MS capillary column (30 m × 0.25 mm x 0.25 µm). 1 µL of each sample was injected in splitless mode using an inlet liner temperature of 240 °C. GC runs were performed with helium as carrier gas at 1 mL/min. The GC oven temperature ramp was from 70 °C to 280 °C. The temperature of 70 °C was held for 2min. Then, the first temperature ramp was from 70 °C to 140 °C at 3 °C/min. The second ramp was from 140 °C to 150 °C at 1 °C/min. The third temperature ramp was from 150 °C to 280 °C at 3 °C/min. Metabolite measurements were performed under electron impact ionization at 70 eV using SIM mode. The ion source and transfer line temperatures were set to 230 °C and 290 °C, respectively. For data analysis, the MS Quantitative Analysis software (version 10.2 Agilent) and an in-house library were used. For relative metabolite abundances, the peak area of each metabolite was normalized to norvaline and to protein concentration.

Statistical analysis was performed on triplicate experiments using Graphpad Prism 10 or Metaboanalyst (MetaboAn:https://www.metaboanalyst.ca/MetaboAnalyst/home.xhtmlalyst). Statistical significance was assessed using an unpaired t-test with unequal variances and FDR correction. Metabolite enrichment analysis, hierarchical clustering and PCA were performed in Metaboanalyst after autoscaling. Over-representation analysis (ORA) was performed using the Consensus Path Database[3] web tool (http://cpdb.molgen.mpg.de/) with the list of significantly modulated metabolites identified by Metaboanalyst as input.

Super-resolution STED microscopy

Cells were grown on highly corrected coverslips (170um ± 5um, Menzel GmbH) and fixed with 4% paraformaldehyde (EM grade, EMS cat.15710) for 20 minutes at room temperature.

Cells were washed three times with PBS and then permeabilized with PBS containing 0.1% Triton X-100 for 15 minutes at room temperature. After three washes with PBS, cells were incubated in blocking buffer (5% normal goat serum in PBS) for 30 minutes at room temperature. Primary antibodies (anti-Pontin 1:50, Sigma Aldrich; anti-TOMM20 1:500 Abcam; anti-ATPB dilution Proteintech, anti-ATP5A1 dilution Proteintech) were incubated in blocking buffer overnight at 4°C and secondary antibodies (Invitrogen's goat anti-mouse AlexaFluor568, goat anti-mouse Alexa Fluor 532, goat anti-rabbit AlexaFluor532 and goat-anti rabbit AlexaFluor555 plus, all diluted 1:100) were incubated for 1 hour at room temperature. Coverslips were mounted in Prolong Glass antifade medium (ThermoFisher Scientific). STimulated Emission Depletion (STED) images were collected through an HCPLAPO100X 1.4NA oil objective with a Leica TCS SP8 STED microscope equipped with a supercontinuum white light laser and a 660nm STED laser and deconvolved using Huygen Professional Software.

Electron microscopy

Samples were fixed with 2% formaldehyde and 2.5% glutaraldehyde in 0.1M cacodylate buffer (pH 7.4) and then embedded in epoxy resin. Ultra-thin sections (~70 nm thick) were mounted on gold grids and immunostained overnight at 4° with a monoclonal mouse primary antibody (anti-pontin, SAB4200194) diluted in filtered 1% BSA (1:100). After washes with filtered PBS-BSA 1% solution, a goat anti-mouse secondary antibody conjugated with 10 nm colloidal gold particles was diluted in filtered 1% BSA (1:20) and incubated in a humid chamber for 2 hours at 37°. Samples were rinsed in two steps, first with filtered PBS-BSA 1% solution and then with pure water. After counterstaining with UranyLess (Electron Microscopy Sciences) and alkaline bismuth subnitrate, samples were observed using a JEM 1010 electron microscope (Jeol, Tokyo, Japan) at 80 kV. Photomicrographs were captured with a digital camera, MegaView III (Soft Imaging System, Muenster, Germany), connected to a computer (Dell, Round Rock, Texas) with dedicated software (AnalySIS, Soft Imaging System, Muenster, Germany).

Single Molecule Localization Microscopy

Cells were fixed in 4% PFA for 10 min, washed in PBS, permeabilized with 0.1% Triton X-100 for 10 min, and incubated in 6 M urea (pH 9.0) for 5 min at 80 °C to improve epitope–antibody binding. Therefore, nonspecific binding sites were blocked by incubation in 0.1% Triton X-100 supplemented with 2% BSA for 45 min at room temperature. Cells were then incubated overnight at 4°C with primary antibodies against RuvBL1 (Rabbit, antibody dilution 1:200, 10210-2-AP, Proteintech) and ATP5A (Mouse, antibody dilution 1:100, ab14748, Abcam). Primary antibodies were detected using CF660 (Donkey Rabbit IgG (H+L) 20816, Biotium) - and CF680 (Donkey Mouse IgG (H+L) 20817, Biotium) -conjugated secondary antibodies. dSTORM imaging was performed in an imaging buffer that included Buffer A (10 mM Tris-HCl pH 8.0 + 50 mM NaCl + 10% Glucose), 0,56 mg/mL Glucose Oxidase (Cohesion Biosciences), 0,34 mg/mL Catalase (Serva) and 50 mM cysteamine (Sigma-Aldrich).

Single-molecule imaging was performed using a SAFe MN360 microscope (Abbelight), equipped with two ORCA-Fusion digital cameras (Hamamatsu) and controlled by Abbelight NEO acquisition software. 2D dual-color SMLM images were acquired using an UPlanApo 100×/NA 1.5 TIRF oil-immersion objective (Olympus) with HiLo illumination. Excitation was performed using 640 nm laser. Dual-color imaging was achieved by spectral demixing using a dual-camera detection scheme. For each acquisition, 45000 frames were recorded with an exposure time of 44 ms.

Image analysis was performed using Abbelight NEO analysis software. Single-molecule localization was carried out prior to image reconstruction. In the reconstructed super-resolution images, each detected molecule was represented as a Gaussian spot centered at its centroid position, with localization precision determined from the single-molecule fitting procedure.

Colocalization analysis was performed using Coloc-Tesseler [DOI: 10.1038/s41467-019-10007-4], which computes Voronoi diagrams from localization data. Colocalization was quantified using the Spearman rank correlation coefficient .

Proximity Ligation Assay (PLA)

For PLA, Huh7 cells were seeded on glass coverslips and incubated for 24h in DMEM/10%FBS. After 24h cells were treated with 1µM of CB-6644 for 72h and then fixed with 4% of paraformaldehyde (20min at room temperature). Cells were permeabilized (0.1% Triton X-100) and incubated with the Blocking buffer included in the PLA kit Naveni™TriFlex Cell. Incubation with primary antibody (overnight at 4°C) and PLA assay were performed following the manufacturer's protocol. Cell nuclei were labelled with DAPI. Images were taken with the high content screening system ScanR (Evident Scientific) with a 40X/0.95 UPlanXApo (Evident Scientific). Quantification of the PLA signal was obtained with the ScanR software.

Western blotting

Proteins were extracted using RIPA assay buffer supplemented with protease and phosphatase inhibitors. Extracts were sonicated and quantified using the BCA assay. Protein samples were resolved on NuPAGE 4-12% Bis-Tris precast SDS-polyacrylamide gels and transferred to polyvinylidene difluoride membranes. Membranes were blocked with 5% skim milk and incubated overnight with primary antibodies in 5% bovine serum albumin (BSA). Subsequently, membranes were incubated for 1 hour at room temperature with horseradish peroxidase-conjugated anti-rabbit IgG (LICOR), diluted 1:50.000 in BSA solution. Protein detection was performed using Enhanced Chemiluminescence Select (Cytiva) and visualized with the ImageQuant350 system (GE Healhcare).

Mitochondrial immunoprecipitation

Huh7 cells endogenously tagged with $OMP25^{HA}$ and $OMP25^{MYC}$ were seeded in 15 cm plates. All buffers were supplemented with protease inhibitors. At 80% confluency, the cells were washed twice with cold PBS containing protease inhibitors (Sigma), then once with cold KPBS buffer (136 mM KCl, 10 mM KH_2PO_4, 50 mM sucrose, pH 7.2) supplemented with protease inhibitors. The cells were harvested on ice by scraping and pelleted at 1000g for 5 min at 4°C. The cell pellet was resuspended in 1 mL KPBS with protease inhibitors and lysed using 30 strokes in a 2 mL homogenizer. The lysate

was spun down at 1000g for 5 min at 4°C. The pellet was discarded, and the input sample was incubated with 50 µL of anti-HA magnetic beads (Pierce). The beads were washed with KPBS three times before incubation with samples. The mixture was placed on gentle rotation for 20 min at 4°C. After incubation, the beads were washed twice with KPBS containing 300 mM NaCl and once with KPBS buffer. The samples were then eluted with 100 µL of KPBS containing 0.5% NP-40 in a thermomixer at 30°C for 20 min. Eluates for mass spectrometry were snap-frozen in liquid nitrogen and stored at -80°C until further processing. The same experiment was repeated two times to obtain a biological triplicate.

Proteomics analysis

Samples were first reduced using dithiothreitol (DTT) and alkylated with iodoacetamide at room temperature. Protein precipitation was achieved by adding 100 % methanol. Precipitates were pelleted for 2 min at 4 °C. The pellets were dried and resuspended in 6 M urea. Peptide digestion was carried out with trypsin (Promega) overnight at 37°C. Tryptic peptides were desalted and dried in a vacuum centrifuge prior to mass spectrometry analysis. The samples were then resuspended in water and 0.1% trifluoracetic acid (TFA) and analysed by mass spectrometry. LC-MS/MS analyses were performed on a Q-Exactive HF-X Orbitrap mass spectrometer (Thermo Fisher Scientific). Peptide separation was carried out using a PepMap RSLC C18 column (75 um × 15 cm, 2 µm, 100 Å, Thermo Fisher) at a flow rate of 300 nl/min. The mobile phases A and B used for the analysis were 0.1% formic acid in water and 0.1% formic acid in acetonitrile, respectively. The gradient started with 5% B and increased to 90% over 120 min. The experiment was performed using a data dependent analysis (DDA) setting to select the "top twenty" most-abundant ions for MS/MS analysis. Proteome Discoverer 2.5 (Thermo Scientific) performed protein identification. The peptide spectra were matched against *Homo sapiens* database downloaded from Uniprot (TaxId: 9606). The analysis was based on at least one unique peptide with a minimum length of seven amino acids and a false discovery rate (FDR) of 0.01. The default peak-picking settings were used to process the raw MS files in MaxQuant [4] (version 1.6.1.0) and its integrated search engine Andromeda [5]. Protein

relative quantification and calculation of statistical significance were carried out using a two-tailed Student's t-test with error correction (p-value < 0.05) and the Benjamini–Hochberg method. Moreover, a volcano plot, summarizing the distribution of differentially expressed proteins was generated with Perseus software (version 1.6.1.1) [6]. Data are available via ProteomeXchange with identifier PXD075574.

Cell fractionation and mitochondria purification

Aml-12, Huh7, Hep3B and HepG2 cells were seeded in 15 cm cell plates. For every cell line, 30 plates were used for mitochondria isolation. Cells were collected and immediately transferred on ice. Samples were washed twice with cold PBS and subsequently homogenized in isolation buffer (250 mM sucrose, 10 mM HEPES, pH 7.4, 1 mM EDTA) supplemented with a protease inhibitor cocktail. The samples were homogenized, and crude mitochondria were isolated following the protocol from Wieckowski et al.[7]. The protein concentration of the isolated fraction was determined using the BCA assay. The fractions were aliquoted and stored at -80°C for subsequent analysis.

Complex V activity

Complex V activity was assessed by a bioluminescent luciferin-luciferase assay [8]. Huh7 cells were seeded onto 13 mm glass coverslips and transiently transfected with a mitochondrially targeted luciferase chimera (MT Luc). After 48 hours of treatment with CB-6644 (0.5 µM or 1 µM), coverslips were mounted in a thermostated perfusion chamber, and real-time luminescence was recorded with a custom-built luminometer. Recordings began with a 30-second baseline in intracellular buffer (IB; 130 mM KCl, 10 mM NaCl, 0.5 mM KH_2PO_4, 1 mM $MgSO_4$, 5 mM sodium succinate, and 20 mM HEPES, pH 7.4), designed to mimic the cytosolic ionic composition. Cells were then perfused with IB containing 25 µM luciferin (IBluc). Within 120-180 seconds, luciferase catalysed light production, reaching a plateau as it reacted with intracellular ATP. Plasma membrane permeabilization was achieved by perfusing cells with 25 µM digitonin (Sigma-Aldrich) in IBluc. After permeabilization, cells were sequentially exposed to IBluc supplemented with 1 mM malic acid and 1 mM glutamic

acid (Sigma-Aldrich), followed by 5 mM ADP (Sigma-Aldrich). The resulting increase in luminescence, measured in counts per second (cps), reflected ATP synthesis driven by Complex V activity in response to exogenous ADP.

Mitochondrial mass and membrane potential analysis

Cells were plated in 96wells in the appropriate culture media and incubated with Mitotracker Deep Red FM (final concentration 200nM) and Calcein AM (final concentration 2uM) in FluorBrite DMEM for 20 minutes at 37°C. After labelling, the plate was imaged with a dual channel Typhoon confocal scanner (GE Healthcare) at a resolution of 25um/line. The integrated intensity of the Mitotracker signal in each well was normalized to the Calcein AM signal and measured with Fiji software[2]. JC-1 labelling (1uM in FluorBrite DMEM, 30' at 37°C) was performed on cells plated on optically clear 4-sectors 35mm dishes (Greiner Bio-One, cat. 627975) to ensure consistent labelling and imaging of control and CB-6644 treated cells within the same experimental session. Images were acquired with a Leica AM6000 microscope equipped with a stage incubator (Pecon), a Leica DFC350FXR2 camera and an HCX PL Fluotar 20x0.4NA objective. The ratio of red to green JC-1 signal was measured with Fiji software.

Cells were incubated for 30 min at 37 °C in a solution of modified Krebs-Ringer buffer (mKRB: 135 mM NaCl, 5 mM KCl, 0.4 mM KH2PO4, 1 mM MgSO4, 20 mM HEPES, 5.5 mM glucose and 1 mM CaCl2 (pH 7.4)) containing 2 nM tetramethyl rhodamine methyl ester (TMRM; Life Technologies, T-668), Verapamil hydrochloride 20µM (Merck KGaA, V4629), Hoechst 33342 1.6 µM (Thermo Fisher, H3570) and SYTOX™ Green Nucleic Acid Stain 170 nM (Thermo Fisher, S7020). Acquisitions were maintained at 37 °C and captured using a UPLXAPO 20X/0.8 air objective on a Olympus IX83 inverted microscope. Excitation was performed at 561 nm and emission was collected at 590-650 nm. Images were analysed using the scanR High-Content Screening Station (Life sciences, Evident). Briefly, Hoechst 33342 signal was used to define the area of cell nuclei and a mask was generated. The intensity of the SYTOX™ Green was measured within the nuclei of all cells

identified in the first step, and all cells positive for the signal were marked as dead. Then, TMRM average fluorescent units (AFU) were quantified in SYTOX™ Green-negative cells, and each condition was compared with the untreated control.

Mitochondrial network morphometry

Huh7 and AML12 cells were grown on coated glass coverslips and then fixed with paraformaldehyde 4% for 10 minutes at room temperature. The cells were permeabilized with PBS/0.05% Triton X-100 for 10 minutes and blocked for 1 hr in PBS/0.05% Triton X-100 containing 3% BSA before incubation with theTOMM20 primary antibody. Secondary antibodies were Alexa Fluor 594-conjugated. DAPI was used to counterstain DNA. Slides were mounted with ProLong Gold Antifade Mountant (Life Technologies). Z-stack acquisitions (51 planes, one each 0,3 μM) were captured from at least six different fields using a confocal laser scanning microscopy (Olympus FV3000) equipped with a 60X oil immersion objective (PLAPON60XOSC2, N.A.1.4). Pixel size was set at 80 nm. Alexa Fluor 594 was excited at 561 nm, and emission was collected at 590-650 nm.

The Z-stacks were deconvolved using Huygens Essential software (Scientific Volume Imaging B.V.) and a theoretical PSF. Following image reconstruction, single cells were isolated from each acquisition and processed in ImageJ (Fiji software) to calculate the number of objects (count), total (sum), mitochondrial density (total mitochondrial volume/cell volume), and per cell average values of: object volume, object sphericity, object compactness, object surface area, object elongation, and object flatness. The threshold for each cell was automatically calculated using the Ridler-Calvard algorithm. Morphological indexes were computed using the 3D suite plugin [9] All data were then grouped in Excel the imported into an R project to calculate principal component analysis. The first two PCA components were then used to cluster using K-means algorithm. The number of clusters was set to 3, corresponding to 3 classes of mitochondrial network: fragmented, intermediate and connected. The percentage of each mitochondrial network morphology class per condition was then calculated.

In silico analysis

RUVBL1 expression in normal liver (TCGA and GTEx) and HCC samples of the TCGA_LIHC cohort was evaluated through the GEPIA2 web tool [10], last accessed on the 18th of January 2026. Overall Survival analysis and most differential survival gene analysis in the LIHC cohort were performed in GEPIA2 using RUVBL1 expression quartiles (75%-25%) as cut-off values for group definition. RUVBL1 expression level in HCC stages was also graphed within GEPIA2.

Gene Set Enrichment Analysis (GSEA) of the LIHC cohort (Firehose Legacy, 373 samples) was performed using the web app GENI [11] with Spearman correlation and default settings (last accessed on the18th of January 2026). The TCGA database was accessed through the cBioPortal for cancer genomics[12] to retrieve RuvBL1 mRNA expression data in the Liver Hepatocellular Carcinoma cohort (LIHC). Patients were assigned to the HI_RUVBL1 or LOW_RUVBL1 groups based on a Z-score of >2. Twenty-nine (8%) of 371 fully sequenced patients were assigned to the HI_RUVBL1 group. The mRNA expression data of genes significantly enriched in the two groups were used to run a Gene Ontology analysis with ClueGo (Cytoscape app).

Statistics

Statistics analysis was performed with GraphPad Prism 10, from three or more replicates. Achieved statistical significance levels and type of test used are reported in figure legends with standard notations: * $p<0.05$, ** $p<0.01$, *** $p<0.001$, **** $p<0.0001$. Exact p-values for each analysis are reported in the supplementary materials.

Supplementary figure legends

Fig. S1. Metabolomics analysis.

A) Principal Components Analysis of metabolomics data in CTRL and CB-6644 treated Huh7 cells. **B)** Enrichment analysis of metabolic pathways in CB-6644 treated Huh7 cells. Both analyses were performed with MetaboAnalyst web tools.

Fig. S2. RuvBL1 knockdown impairs mitochondrial respiration.

A) Line graph: Seahorse MitoStress Test profiles of RuvBL1-silenced human cell lines. OCR values are normalized by cell number and scaled relative to the basal OCR of non-silenced cells (mean ± SEM). Bar-graph: Quantification of the basal respiratory capacity shown in panel **A (**mean ± SD). Statistical significance was calculated by one-way ANOVA with Dunnett's correction for multiple comparisons. * $p<0.05$, ** $p<0.01$, *** $p<0.001$, **** $p<0.0001$. **B)**. RuvBL1 mRNA expression in control and RuvBL1-silenced cells. Representative qPCR analysis of RuvBL1 inhibition in the three cell lines. Statistical significance (vs Neg Ctrl siN1, mean ± SD) was calculated by 1-way ANOVA with Dunnett's correction for multiple comparisons. * $p<0.05$, ** $p<0.01$, *** $p<0.001$, **** $p<0.0001$.

Fig. S3. Inhibition of RuvBL1/2 ATPase activity impairs OXPHOS and ATP production.

A) Line graph: Seahorse MitoStress profile of cell lines and primary mouse hepatocytes treated with CB-6644 for 24h (mean ± SEM, n=3 to 5 independent experiments). OCR values are normalized by cell number and scaled relative to the basal OCR of non-treated cells. Bar-graph: Quantification of the basal respiratory capacity shown in panel **A (**mean ± SD). Statistical significance was calculated by one-way ANOVA with Dunnett's correction for multiple comparisons. * $p<0.05$, ** $p<0.01$, *** $p<0.001$, **** $p<0.0001$. **B)** ATP-rate assay showing the relative contribution of glycolysis and OXPHOS to the total ATP production in cells treated with CB-6644 for 24h. Statistical significance

(vs CTRL, mean ± SEM) was calculated by 2-way ANOVA with Dunnett's correction for multiple comparisons. * p<0.05, ** p<0.01, *** p<0.001, **** p<0.0001.

C) Line graph: Seahorse MitoStress profile of cell lines and primary mouse hepatocytes treated with CB-6644 for 48h (mean ± SEM, n=3 to 5 independent experiments). OCR values are normalized by cell number and scaled relative to the basal OCR of non-treated cells. Bar-graph: Quantification of the basal respiratory capacity shown in panel **A (**mean ± SD). Statistical significance was calculated by one-way ANOVA with Dunnett's correction for multiple comparisons. * p<0.05, ** p<0.01, *** p<0.001, **** p<0.0001. **D)** ATP-rate assay showing the relative contribution of glycolysis and OXPHOS to the total ATP production in cells treated with CB-6644 for 48h. Statistical significance (vs CTRL, mean ± SEM) was calculated by 2-way ANOVA with Dunnett's correction for multiple comparisons. * p<0.05, ** p<0.01, *** p<0.001, **** p<0.0001.

Fig. S4. Inhibition of RuvBL1/2 ATPase activity increases mitochondrial polarization.

A) Relative Mitotracker intensity measured in cell lines and primary mouse hepatocytes exposed to the indicated doses of CB-6644 for 72h. Statistical significance was calculated by one-way ANOVA with Dunnett's correction for multiple comparisons (median, min to max, n=3). * p<0.05, ** p<0.01.

B) Quantification of oxidized to reduced JC-1 ratio in cells exposed to CB-6644 for 72h. Statistical significance was calculated by one-way ANOVA with Dunnett's correction for multiple comparisons (median, min to max, n=3). * p<0.05, ** p<0.01, *** p<0.001, ****. p<0.0001.

C, D) Analysis of steady state mitochondrial membrane potential through high content confocal imaging of the potentiometric dye TMRM in AML-12 (**C**) and Huh7 (**D**). Statistical significance was calculated by one-way ANOVA with Dunnett's correction for multiple comparisons (median, min to max, n=3). * p<0.05, ** p<0.01**E)** Analysis of mitochondrial hyperpolarization in AML-12 cells exposed to CB-6644 (0.5 μM, 72 hours) or vehicle. Mitochondrial membrane potential was assessed by confocal imaging using the potentiometric dye TMRM. Cells were then challenged with ATP synthase inhibition via oligomycin (1 μM). Hyperpolarization was quantified as the change in TMRM

fluorescence intensity after 30 minutes of oligomycin treatment, relative to baseline values (median, min to max, n=3). Statistical significance was calculated by non-parametric Mann- Whitney test. * $p<0.05$.

Fig. S5. CB-6644 alters mitochondrial morphology.

TEM images depicting the morphology of mitochondria in CTRL and CB-6644-treated cell lines (0.25 uM for 48h). Loss of mitochondrial matrix electron density, cristae swelling and disruption are visible in CB-6644 treated cells. Original magnifications: 50k for Huh7, Hep3B, Hepa1-6, 80k for AML-12 and HepG2.

Fig. S6. CB-6644 promotes OPA-1 cleavage and ATPAF2 expression.

A) WB analysis of RuvBL1 in mitochondrial/cytosol fractionation of human cell lines. TOMM20 and Vinculin were used as positive control of mitochondria fraction and cytosol fraction respectively. **B)** WB of OPA-1 fragments in Huh7 cells treated for 48h with CB-6644 or for 24h with oligomycin 2.5 uM. **C)** WB analysis of ATPAF2 in immunoprecipitated mitochondria of CTRL and CB-6644 (0.5uM, 48h) -treated Huh7. **D)** Representative kinetic of light emitted by mitochondrial-targeted firefly luciferase expressed in Huh7 during the determination of ATP synthase activity. Huh7 cells were exposed to intracellular buffer supplemented with luciferin 25 μM (IBluc), then permeabilized with digitonin 20 μM. After successful permeabilization of the plasma membrane (represented by drop in light emission) mitochondria were energized by supplementation with malate 1 mM and glutamate 1mM. ATP synthesis was then stimulated by the administration of 5 mM ADP. **E)** STORM (Single Molecule Localization Microscopy) microscopy of RuvBL1 (red) and ATP5A (green) in Huh7 cell lines. Representative colocalization map and images of analysed cells. Scale bar = 2um for the larger field and =1um for the enlarged detail. Correlation analysis calculated by Spearman coefficient (mean ± SD).

Fig. S7. Mitoproteome analysis of CB-6644 treated Huh7 cells.

A) Mitochondrial proteins selectively detected in immunoprecipitated mitochondria of CTRL or CB-6644-treated cells (1 uM for 48h). **B)** Volcano plot and table of mitochondrial proteins differentially expressed in CTRL or CB-6644 treated cells.

Fig. S8. RuvBL1 expression correlates with disease progression and metabolism in human HCC.

A) RUVBL1 positively correlates with stage in the TCGA_LIHC cohort. Analysis performed with GEPIA2 **B)** RUVBL1 scores within the 10 top enriched genes affecting OS in the TCGA_LIHC cohort. Analysis performed with GEPIA2 **C)** Phosphorylation of mTOR S2448 is significantly correlated with RUVBL1 expression in the TGCA_LIHC cohort (RUVBL1 cut-off value Z±0.67, performed with cBioportal). **D)** GSEA analysis showing inverse correlation between RUVBL1 expression and ketogenic pathways in TCGA_LIHC cohort (performed with GENI).

Fig. S9. RuvBL1 correlation in human normal liver and HCC samples.

The analyses were performed through the GEPIA2 web portal. **A)** RUVBL1-AST correlation, **B)** RUVBL1-ALT correlation, **C)** RUVBL1-ASNS correlation, **D)** Correlation of RUVBL1 with conserved genes of involved in the propionate shunt (ACADSB, ECHS1, HIBCH, ADHFE1, ALDH6A1)[13].

Supplementary figures

Fig. S1

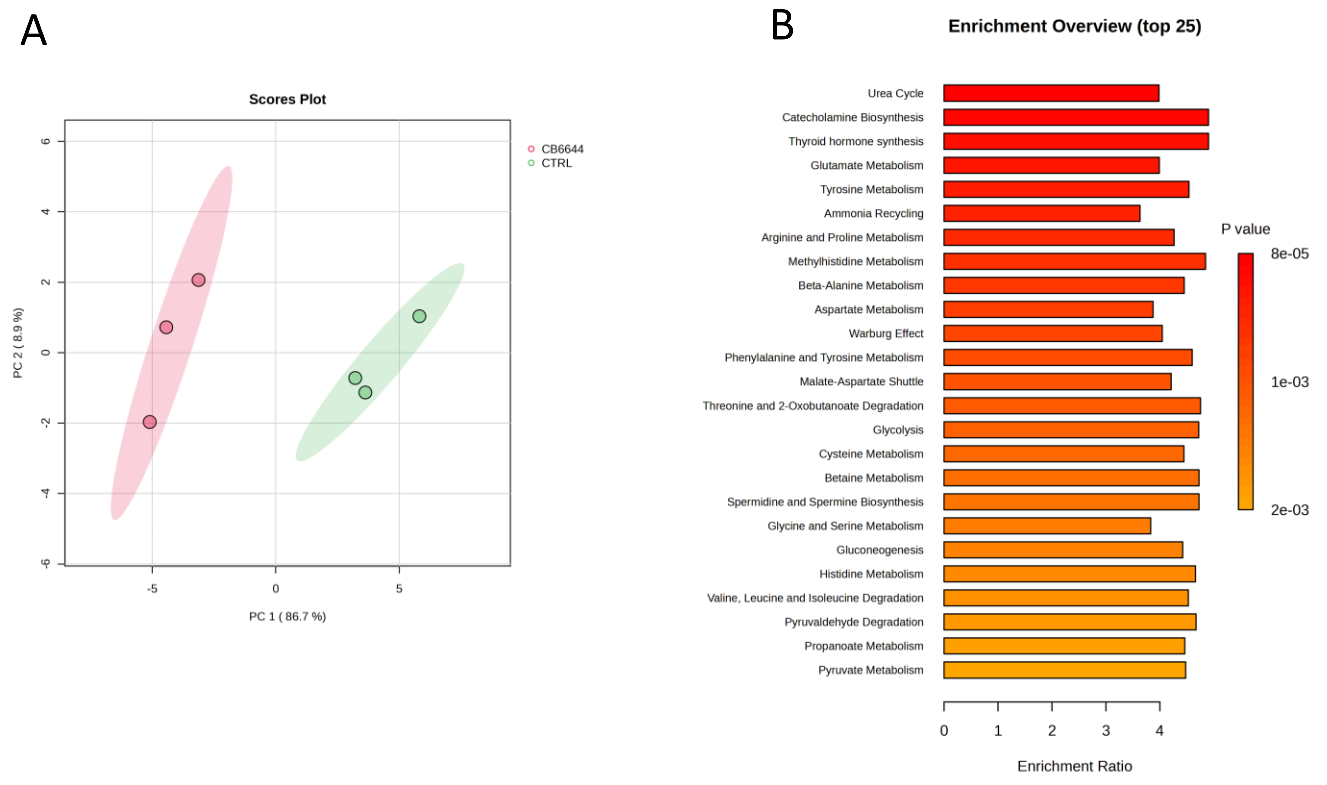

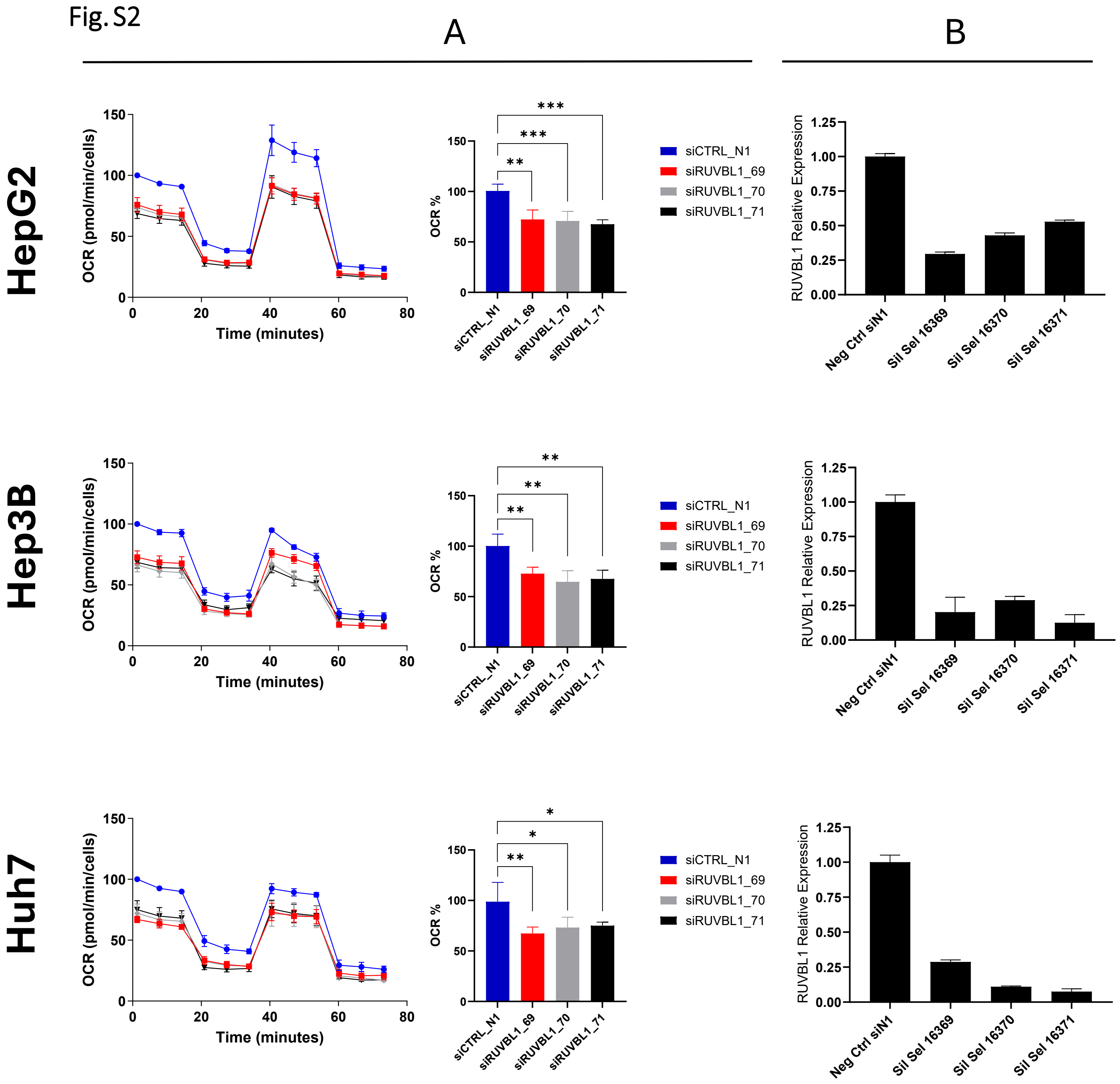

Fig. S2
A
B
HepG2
Hep3B
Huh7
OCR (pmol/min/cells)
Time (minutes)
OCR %
siCTRL_N1
siRUVBL1_69
siRUVBL1_70
siRUVBL1_71
RUVBL1 Relative Expression
Neg Ctrl siN1
Sil Sel 16369
Sil Sel 16370
Sil Sel 16371

Fig. S3

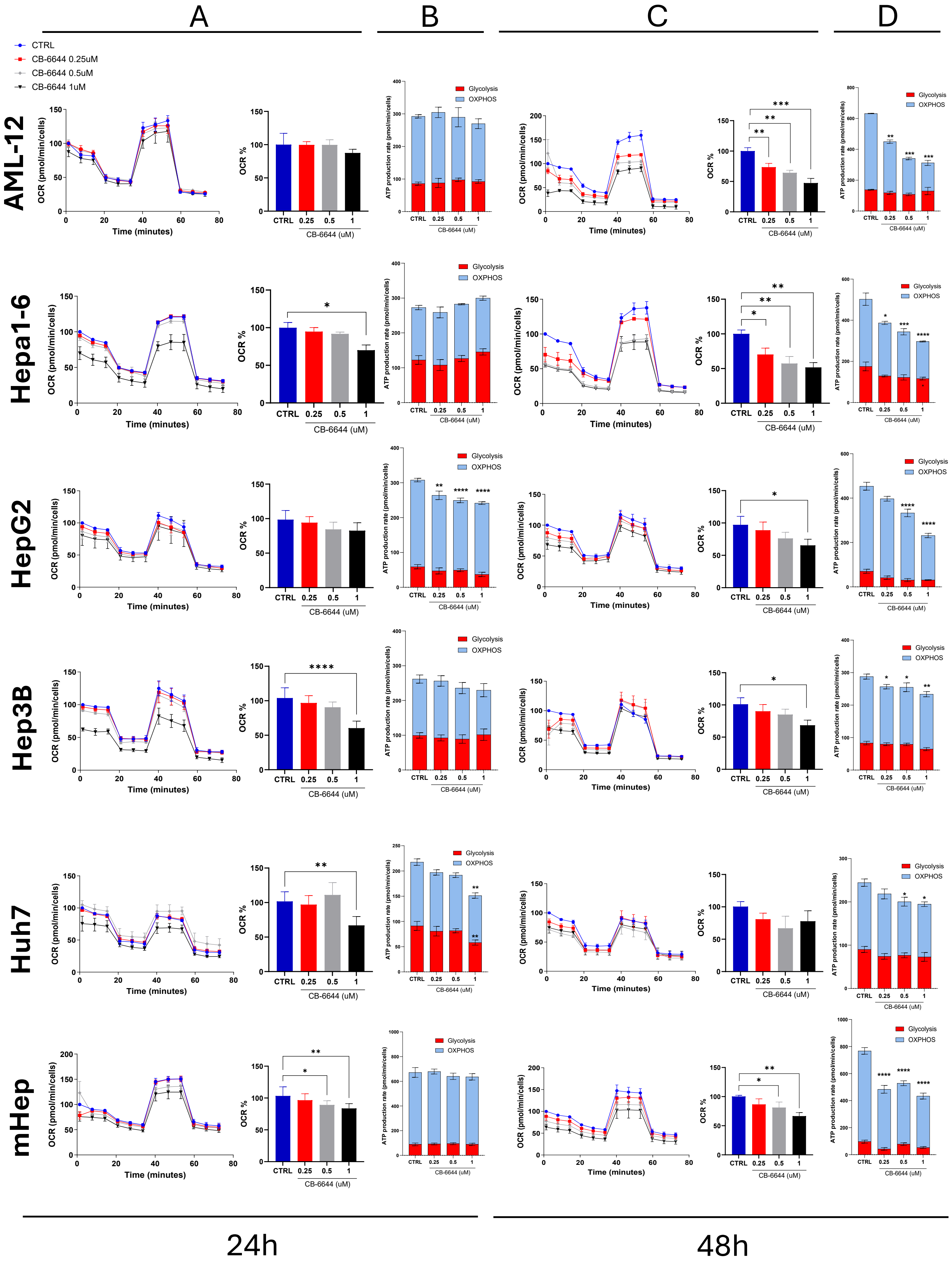
A
B
C
D
CTRL
CB-6644 0.25uM
CB-6644 0.5uM
CB-6644 1uM
AML-12
Hepa1-6
HepG2
Hep3B
Huh7
mHep
OCR (pmol/min/cells)
Time (minutes)
OCR %
CB-6644 (uM)
ATP production rate (pmol/min/cells)
Glycolysis
OXPHOS
24h
48h

Fig. S4

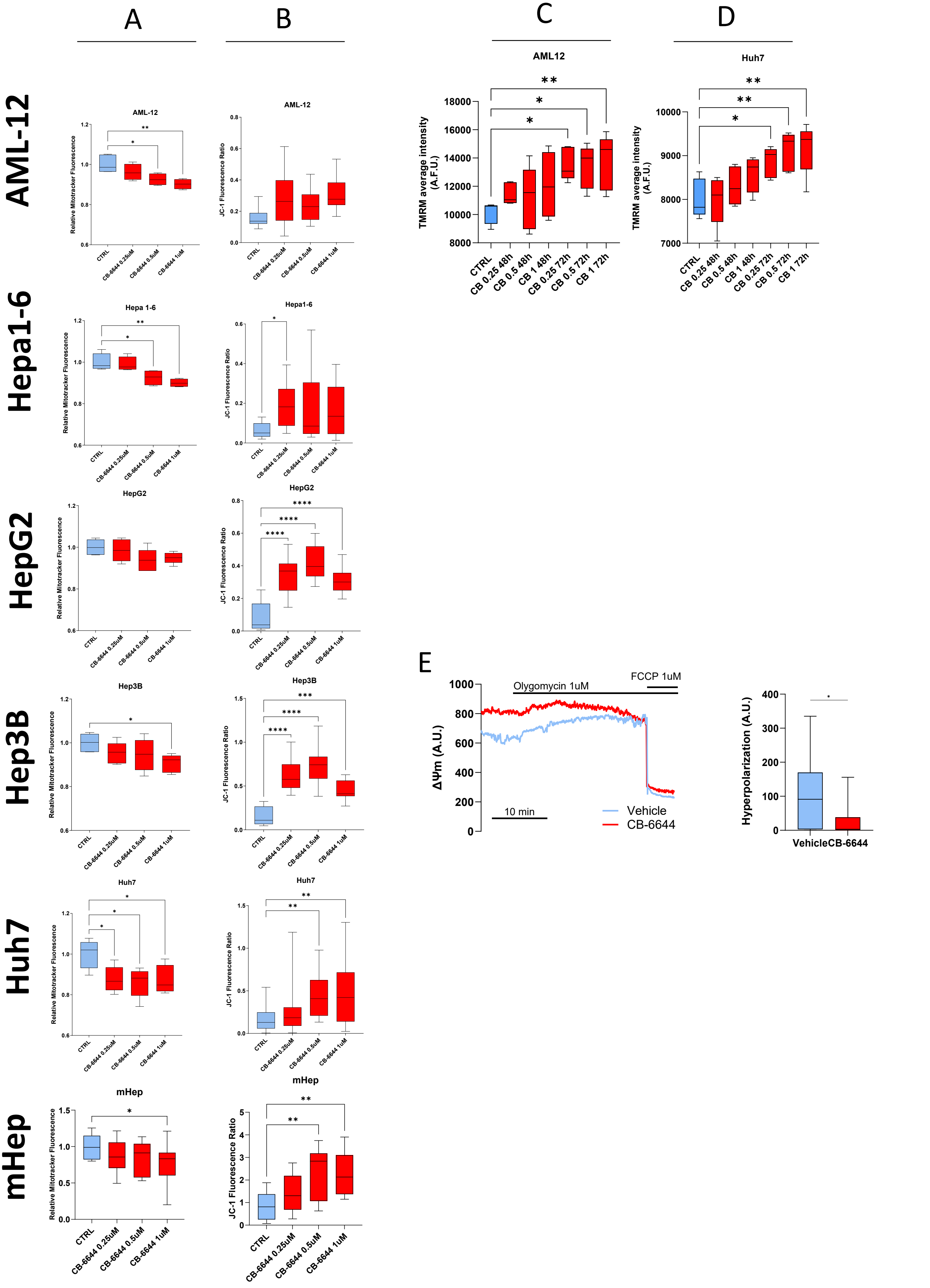
A
B
C
D
E
AML-12
Hepa1-6
HepG2
Hep3B
Huh7
mHep
AML12
Huh7
Relative Mitotracker Fluorescence
JC-1 Fluorescence Ratio
TMRM average intensity (A.F.U.)
CTRL
CB-6644 0.25uM
CB-6644 0.5uM
CB-6644 1uM
CB 0.25 48h
CB 0.5 48h
CB 1 48h
CB 0.25 72h
CB 0.5 72h
CB 1 72h
Olygomycin 1uM
FCCP 1uM
ΔΨm (A.U.)
10 min
Vehicle
CB-6644
Hyperpolarization (A.U.)
VehicleCB-6644

Fig. S5

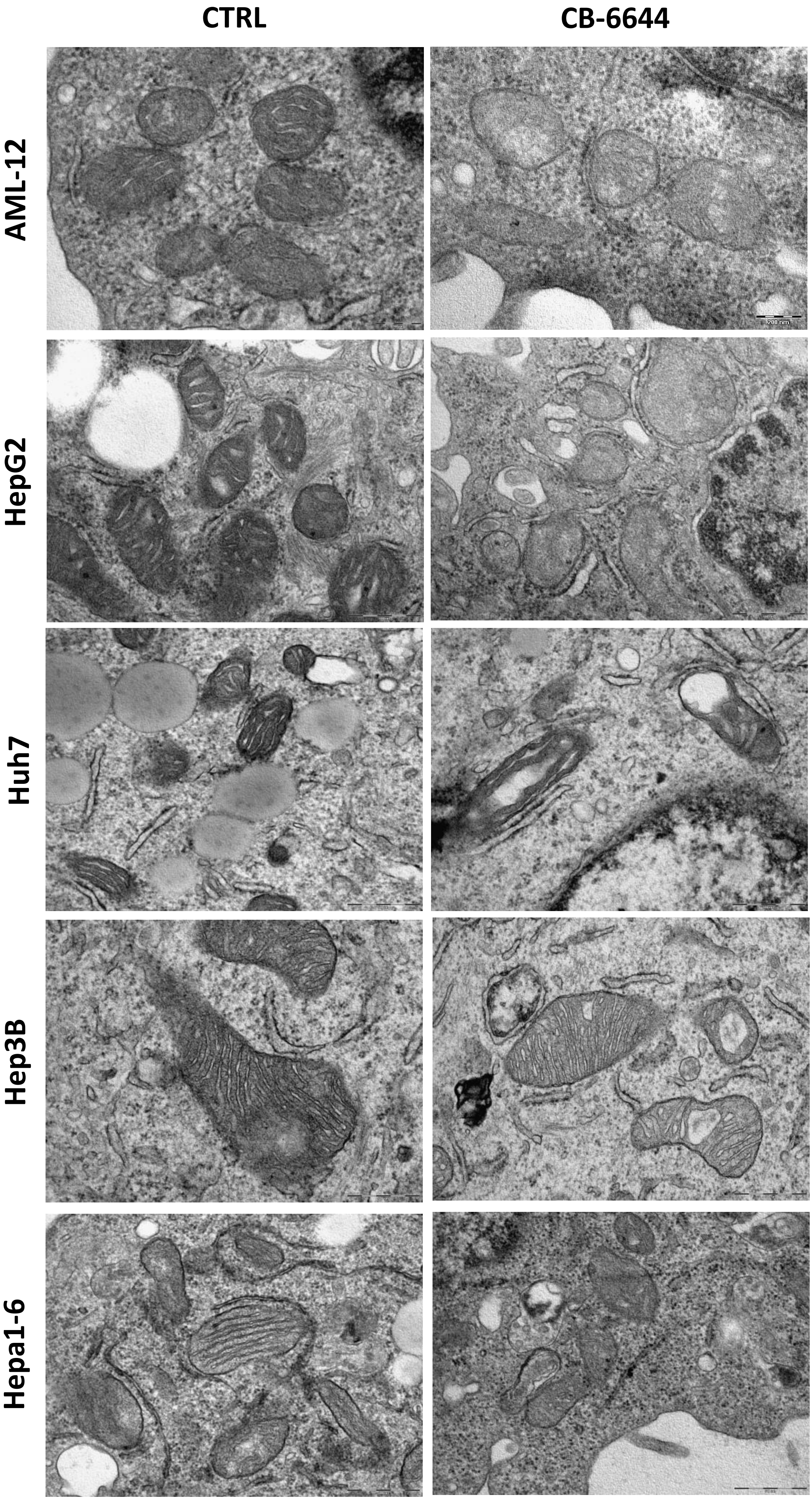

Fig. S6

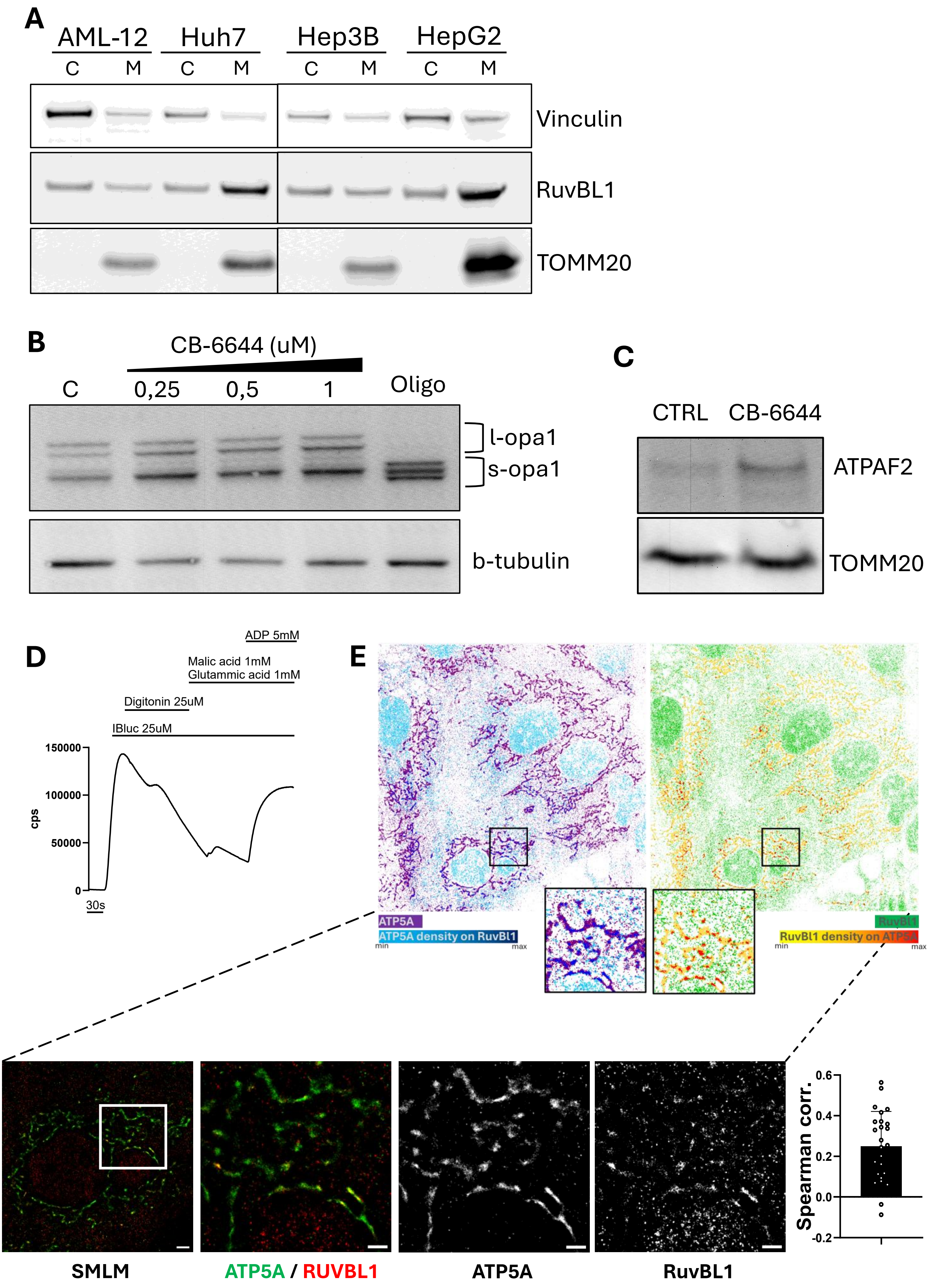
A
AML-12
Huh7
Hep3B
HepG2
C M C M C M C M
Vinculin
RuvBL1
TOMM20
B
CB-6644 (uM)
C
0,25
0,5
1
Oligo
l-opa1
s-opa1
b-tubulin
C
CTRL
CB-6644
ATPAF2
TOMM20
D
ADP 5mM
Malic acid 1mM
Glutammic acid 1mM
Digitonin 25uM
IBluc 25uM
150000
100000
50000
0
cps
30s
E
ATP5A
ATP5A density on RuvBl1
min
max
RuvBl1
RuvBl1 density on ATP5A
min
max
SMLM
ATP5A / RUVBL1
ATP5A
RuvBL1
Spearman corr.
0.6
0.4
0.2
0.0
-0.2

Fig. S7

A

FDR confidence	Accession	Description	Gene Symbol	Group
High	P08133	Annexin A6	ANXA6	CTRL only
High	P01116	GTPase KRas	KRAS	CTRL only
High	Q86TS9	39S ribosomal protein L52, mitochondrial	MRPL52	CTRL only
High	Q9P2J9	Pyruvate dehydrogenase phosphatase 2, mitochondrial	PDP2	CTRL only
High	P36873	Serine/threonine-protein phosphatase PP1-gamma catalytic subunit	PPP1CC	CTRL only
High	O00743	Serine/threonine-protein phosphatase 6 catalytic subunit	PPP6C	CTRL only
High	Q8N357	Solute carrier family 35 member F6	SLC35F6	CTRL only
High	Q96QK1	Vacuolar protein sorting-associated protein 35	VPS35	CTRL only
High	Q13686	Nucleic acid dioxygenase ALKBH1	ALKBH1	CB-6644 only
High	Q9UHK6	Alpha-methylacyl-CoA racemase	AMACR	CB-6644 only
High	P0C7P0	CDGSH iron-sulfur domain-containing protein 3, mitochondrial	CISD3	CB-6644 only
High	M0R0L2	Coenzyme Q8B	COQ8B	CB-6644 only
High	Q8N465	D-2-hydroxyglutarate dehydrogenase, mitochondrial	D2HGDH	CB-6644 only
High	Q5T440	Putative transferase CAF17, mitochondrial	IBA57	CB-6644 only
High	Q86U28	Iron-sulfur cluster assembly 2 homolog, mitochondrial	ISCA2	CB-6644 only
High	Q96AQ8	Mitochondrial calcium uniporter regulator 1	MCUR1	CB-6644 only
High	Q9BV79	Enoyl-[acyl-carrier-protein] reductase, mitochondrial	MECR	CB-6644 only
High	O95822	Malonyl-CoA decarboxylase, mitochondrial	MLYCD	CB-6644 only
High	Q9H019	Mitochondrial fission regulator	MTFR1L	CB-6644 only
High	Q9ULD0	2-oxoglutarate dehydrogenase-like, mitochondrial	OGDHL	CB-6644 only
High	Q8NI37	Protein phosphatase PTC7 homolog	PPTC7	CB-6644 only

B

Accession	Description	Gene Symbol	Group	Expression difference (CTRL-CB6644)	-Log(p-value)
A0A0A0MT83	Isovaleryl-CoA dehydrogenase, mitochondrial	IVD	UP in CB-6644	1,953	2,882
P35914	Hydroxymethylglutaryl-CoA lyase, mitochondrial	HMGCL	UP in CB-6644	1,323	4,354
Q16775	Hydroxyacylglutathione hydrolase, mitochondrial	HAGH	UP in CB-6644	1,213	4,653
Q6NUM9	All-trans-retinol 13,14-reductase	RETSAT	UP in CB-6644	2,385	2,627
Q8N5M1	ATP synthase mitochondrial F1 complex assembly factor 2	ATPAF2	UP in CB-6644	1,312	3,325

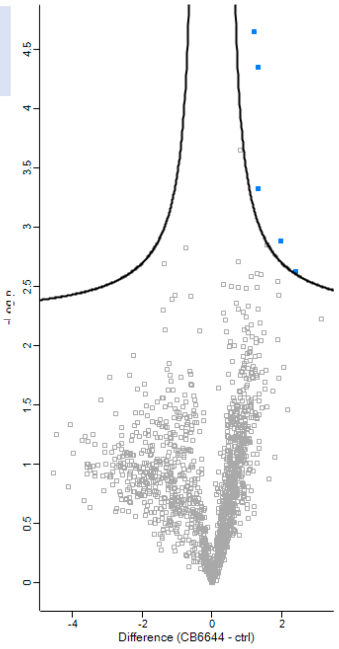

Fig. S8

A

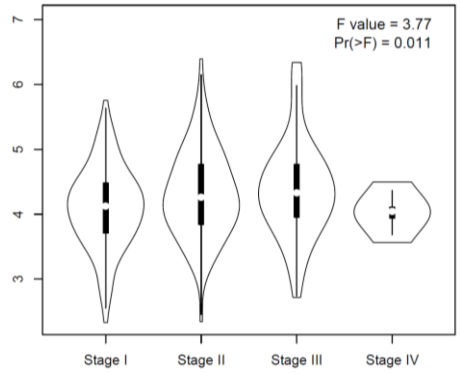

B

Gene Symbol	Gene ID	P-Value (Survival os)
PTDSS2	ENSG00000174915.11	1.82e-9
PIGU	ENSG00000101464.10	1.09e-8
UCK2	ENSG00000143179.12	5.65e-8
KPNA2	ENSG00000182481.8	7.20e-8
HILPDA	ENSG00000135245.9	8.48e-8
MED19	ENSG00000156603.14	1.34e-7
GTPBP4	ENSG00000107937.18	1.80e-7
RUVBL1	ENSG00000175792.11	1.86e-7
CCT5	ENSG00000150753.11	1.90e-7
KB-68A7.1	ENSG00000274225.1	2.01e-7

C

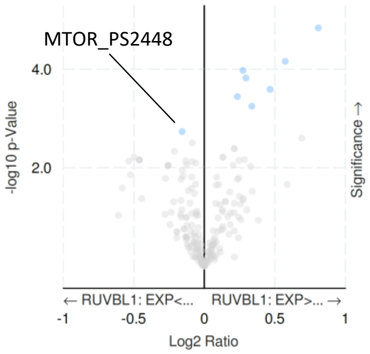

D

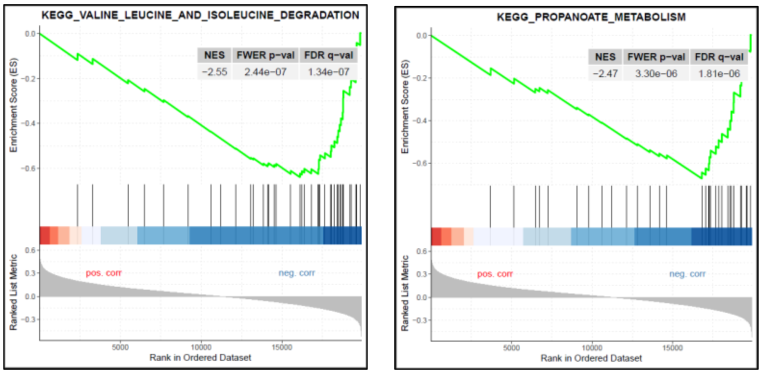

Fig. S9

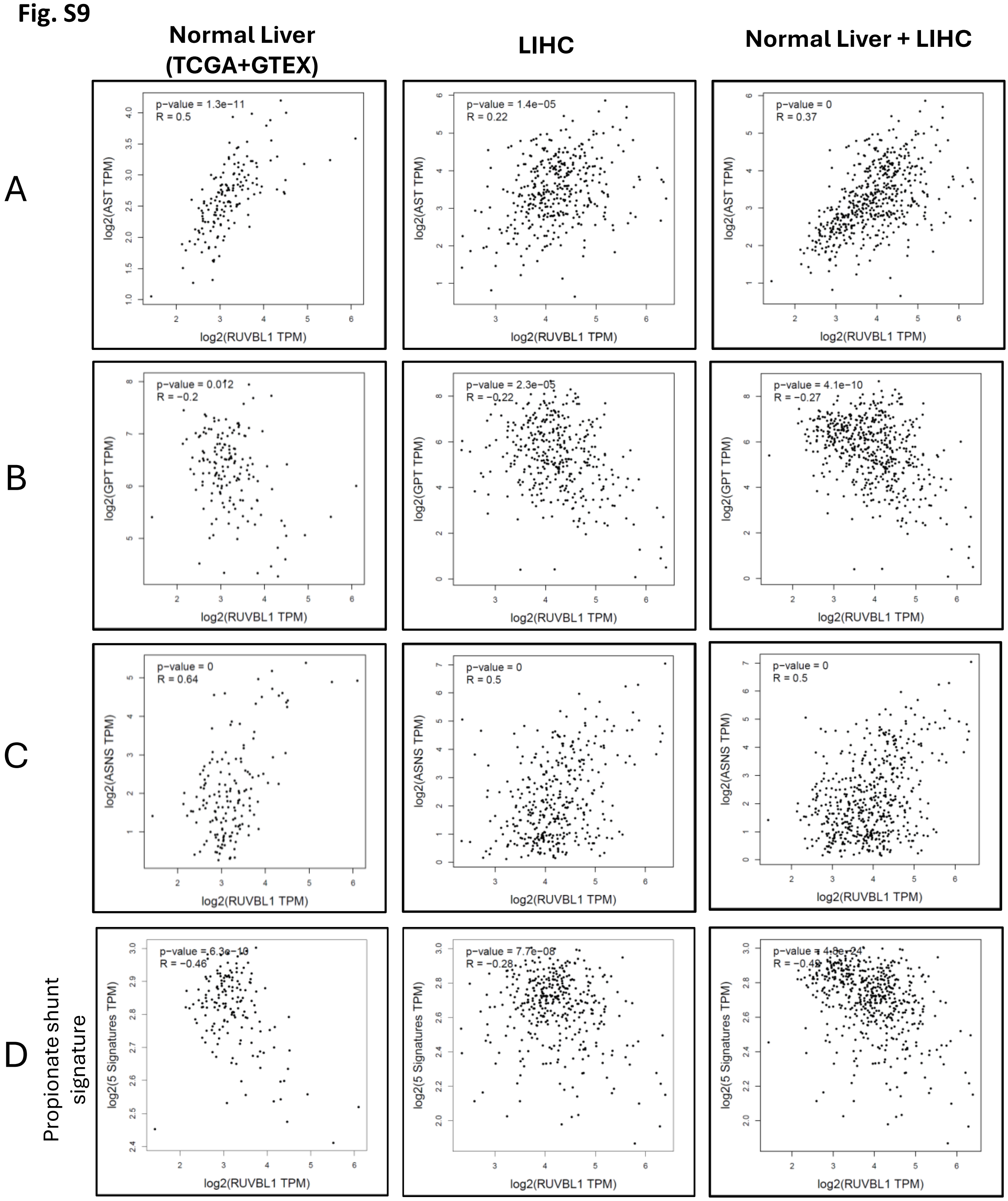
Normal Liver (TCGA+GTEX)
LIHC
Normal Liver + LIHC
A
p-value = 1.3e-11
R = 0.5
p-value = 1.4e-05
R = 0.22
p-value = 0
R = 0.37
log2(AST TPM)
B
p-value = 0.012
R = -0.2
p-value = 2.3e-05
R = -0.22
p-value = 4.1e-10
R = -0.27
log2(GPT TPM)
C
p-value = 0
R = 0.64
p-value = 0
R = 0.5
p-value = 0
R = 0.5
log2(ASNS TPM)
D
Propionate shunt signature
p-value = 6.3e-10
R = -0.46
p-value = 7.7e-08
R = -0.28
p-value = 4.8e-24
log2(5 Signatures TPM)
log2(RUVBL1 TPM)

Table S1. Metabolic pathways modulated by CB-6644 in Huh7 cells.

Functional annotation of the metabolite set modulated by CB-6644 (0.5uM for 48h) in Huh7 cells. Pathway annotation was performed using the Consensus Path Database. KEGG, Reactome and Wikipathway annotation are reported.

p-value	q-value	pathway	source
6,62E-38	4,11E-36	Central carbon metabolism in cancer - Homo sapiens (human)	KEGG
6,44E-30	2,00E-28	Amino Acid metabolism	Wikipathways
6,63E-25	1,37E-23	Transport of inorganic cations/anions and amino acids/oligopeptides	Reactome
2,71E-24	3,38E-23	Transport of bile salts and organic acids, metal ions and amine compounds	Reactome
2,73E-24	3,38E-23	SLC-mediated transmembrane transport	Reactome
4,41E-24	4,56E-23	Transport of small molecules	Reactome
6,21E-23	5,50E-22	Protein digestion and absorption - Homo sapiens (human)	KEGG
3,18E-22	2,47E-21	Aminoacyl-tRNA biosynthesis - Homo sapiens (human)	KEGG
4,30E-22	2,96E-21	Biochemical Pathways Part I	Wikipathways
3,21E-21	1,99E-20	Glucose Homeostasis	Wikipathways
1,92E-19	1,08E-18	Na+/Cl- dependent neurotransmitter transporters	Reactome
3,08E-19	1,59E-18	Amino acid transport across the plasma membrane	Reactome
5,31E-17	2,13E-16	tRNA Aminoacylation	Reactome
5,31E-17	2,13E-16	Cytosolic tRNA aminoacylation	Reactome
5,31E-17	2,13E-16	Mitochondrial tRNA aminoacylation	Reactome
5,50E-17	2,13E-16	Mineral absorption - Homo sapiens (human)	KEGG
4,39E-16	1,60E-15	Translation	Reactome
2,65E-15	9,13E-15	Metabolism of amino acids and derivatives	Reactome
1,59E-14	5,17E-14	Tryptophan catabolism	Reactome
6,50E-13	2,02E-12	Amino Acid Metabolism Pathway Excerpt (Histidine catabolism extension)	Wikipathways
1,15E-12	3,36E-12	Metabolism of proteins	Reactome
1,23E-12	3,36E-12	ABC transporters - Homo sapiens (human)	KEGG
1,25E-12	3,36E-12	Metabolic reprogramming in colon cancer	Wikipathways
1,80E-12	4,64E-12	Alanine and aspartate metabolism	Wikipathways
9,79E-12	2,43E-11	Metabolism	Reactome
3,61E-11	8,61E-11	Glucagon signaling pathway - Homo sapiens (human)	KEGG
6,46E-11	1,48E-10	Alanine, aspartate and glutamate metabolism - Homo sapiens (human)	KEGG
3,58E-10	7,85E-10	Glucose metabolism	Reactome
3,67E-10	7,85E-10	TCA Cycle and Deficiency of Pyruvate Dehydrogenase complex (PDHc)	Wikipathways
5,23E-10	1,08E-09	Citrate cycle (TCA cycle) - Homo sapiens (human)	KEGG
4,06E-09	8,12E-09	Glycolysis and Gluconeogenesis	Wikipathways
4,94E-09	9,57E-09	Gluconeogenesis	Reactome
5,83E-09	1,10E-08	The citric acid (TCA) cycle and respiratory electron transport	Reactome
1,17E-08	2,14E-08	Phenylalanine and tyrosine metabolism	Reactome
5,63E-08	9,97E-08	Pyruvate metabolism and Citric Acid (TCA) cycle	Reactome

6,24E-08	1,07E-07	Phenylalanine metabolism	Reactome
8,88E-08	1,49E-07	Metabolism overview	Wikipathways
1,02E-07	1,63E-07	Glutamate and glutamine metabolism	Reactome
1,02E-07	1,63E-07	Valine, leucine and isoleucine biosynthesis - Homo sapiens (human)	KEGG
4,23E-07	6,55E-07	Citric acid cycle (TCA cycle)	Reactome
5,93E-07	8,96E-07	Pyruvate metabolism - Homo sapiens (human)	KEGG
7,14E-07	1,05E-06	Glyoxylate and dicarboxylate metabolism - Homo sapiens (human)	KEGG
9,27E-07	1,34E-06	Urea cycle and associated pathways	Wikipathways
1,02E-06	1,43E-06	Metabolism of carbohydrates	Reactome
5,84E-06	8,05E-06	TCA Cycle (aka Krebs or citric acid cycle)	Wikipathways
7,31E-06	9,85E-06	Metabolism of vitamins and cofactors	Reactome
1,07E-05	1,42E-05	Metabolism of water-soluble vitamins and cofactors	Reactome
1,76E-05	2,28E-05	Pantothenate and CoA biosynthesis - Homo sapiens (human)	KEGG
2,60E-05	3,29E-05	Glyoxylate metabolism and glycine degradation	Reactome
4,61E-05	5,71E-05	Branched-chain amino acid catabolism	Reactome
1,38E-04	1,64E-04	Lysine degradation - Homo sapiens (human)	KEGG
1,38E-04	1,64E-04	Glycine, serine and threonine metabolism - Homo sapiens (human)	KEGG
2,81E-04	3,28E-04	Phenylalanine metabolism - Homo sapiens (human)	KEGG
3,39E-04	3,89E-04	Cysteine and methionine metabolism - Homo sapiens (human)	KEGG
6,09E-04	6,87E-04	GPCR downstream signalling	Reactome
1,55E-03	1,72E-03	Signaling by GPCR	Reactome
1,62E-03	1,76E-03	Signal Transduction	Reactome
3,45E-03	3,69E-03	Fatty acid metabolism	Reactome
5,93E-03	6,12E-03	Metabolism of nucleotides	Reactome
5,93E-03	6,12E-03	Post-translational protein modification	Reactome
6,57E-03	6,67E-03	GPCR ligand binding	Reactome

Supplementary references

[1] Chen WW, Freinkman E, Sabatini DM. Rapid immunopurification of mitochondria for metabolite profiling and absolute quantification of matrix metabolites. Nat Protoc 2017;12:2215–31. https://doi.org/10.1038/nprot.2017.104.

[2] Schindelin J, rganda-Carreras I, Frise E, et al. Fiji: an open-source platform for biological-image analysis. Nature Methods 2012;9:676.

[3] Herwig R, Hardt C, Lienhard M, et al. Analyzing and interpreting genome data at the network level with ConsensusPathDB. Nat Protoc 2016;11:1889–907. https://doi.org/10.1038/nprot.2016.117.

[4] Cox J, Mann M. MaxQuant enables high peptide identification rates, individualized p.p.b.-range mass accuracies and proteome-wide protein quantification. Nat Biotechnol 2008;26:1367–72. https://doi.org/10.1038/nbt.1511.

[5] Cox J, Neuhauser N, Michalski A, et al. Andromeda: A Peptide Search Engine Integrated into the MaxQuant Environment. J Proteome Res 2011;10:1794–805. https://doi.org/10.1021/pr101065j.

[6] Tyanova S, Temu T, Sinitcyn P, et al. The Perseus computational platform for comprehensive analysis of (prote)omics data. Nat Methods 2016;13:731–40. https://doi.org/10.1038/nmeth.3901.

[7] Wieckowski MR, Giorgi C, Lebiedzinska M, et al. Isolation of mitochondria-associated membranes and mitochondria from animal tissues and cells. Nat Protoc 2009;4:1582–90. https://doi.org/10.1038/nprot.2009.151.

[8] Morciano G, Sarti AC, Marchi S, et al. Use of luciferase probes to measure ATP in living cells and animals. Nat Protoc 2017;12:1542–62. https://doi.org/10.1038/nprot.2017.052.

[9] Ollion J, Cochennec J, Loll F, et al. TANGO: a generic tool for high-throughput 3D image analysis for studying nuclear organization. Bioinformatics 2013;29:1840–1. https://doi.org/10.1093/bioinformatics/btt276.

[10] **Tang Z**, **Li C**, Kang B, et al. GEPIA: a web server for cancer and normal gene expression profiling and interactive analyses. Nucleic Acids Res 2017;45:W98–102.

[11] Hayashi A, Ruppo S, Heilbrun EE, et al. GENI: A web server to identify gene set enrichments in tumor samples. Computational and Structural Biotechnology Journal 2023;21:5531–7. https://doi.org/10.1016/j.csbj.2023.10.053.

[12] Gao J, Aksoy BA, Dogrusoz U, et al. Integrative analysis of complex cancer genomics and clinical profiles using the cBioPortal. SciSignal 2013;6:l1.

[13] Watson E, Olin-Sandoval V, Hoy MJ, et al. Metabolic network rewiring of propionate flux compensates vitamin B12 deficiency in C. elegans. eLife n.d.;5:e17670. https://doi.org/10.7554/eLife.17670.
