# Supplementary material for: Targeting RuvBL1 disrupts mitochondrial metabolism and structure in hepatocellular carcinoma
Source: JHEP Rep. 2026 Apr 17;8(7):101858. doi: 10.1016/j.jhepr.2026.101858 (PMC13310627; doi:10.1016/j.jhepr.2026.101858)
Supplement: Multimedia component 2 [file mmc2.docx]

**JHEP Reports**

**CTAT methods**

**If the CTAT form is not relevant to your study, please outline the reasons why:**

|  |
| --- |

- 1. **Antibodies**

| **Name** | **Citation** | **Supplier** | **Cat no.** | **Clone no.** |
| --- | --- | --- | --- | --- |
| RUVBL1 |  | Proteintech | 10210-2-AP |  |
| GAPDH |  | Sigma Aldrich | G9545 |  |
| TOMM20 |  | Abcam | AB186735 |  |
| ATPAF2 |  | ThermoFischer Scientific | PA5101441 |  |
| OXPHOS kit |  | Invitrogen | 45-8099 |  |
| IMMT(Mic60) |  | Sigma Aldrich | HPA036164 |  |
| CHCHD3(Mic19) |  | Sigma Aldrich | HPA042935 |  |
| ATPIF1 |  | Cell Signalling Technology | 13268S |  |
| OPA1 |  | Proteintech | 27733-1-AP |  |
| Beta tubulin |  | Sigma Aldrich | T5201 |  |
| HA |  | Cell Signalling Technology | C29F4 |  |
| ATP5B |  | Santa cruz | sc 74549 |  |
| ATP5A |  | Abcam | ab14748 |  |
| ATPG |  | Abcam | ab119686 |  |
| ATP5H |  | Abcam | ab110275 |  |
| ATP5L |  | Abcam | ab126181 |  |
| ATP5I |  | Abcam | ab122241 |  |
| Citrate synthase |  | Cell Signalling Technology | cs 14309s |  |
| TIM23 |  | BD Biosciences | BD611222 |  |
| Pontin |  | Sigma Aldrich | SAB4200194 | 5G3-11 |
| Goat anti-rabbit IgG (H+L) highly cross-adsorbed, Alexa Fluor 488 |  | Invitrogen | A11034 |  |
| Goat anti-Mouse IgG (H+L) highly cross-adsorbed Secondary Antibody, Alexa Fluor 568 |  | Invitrogen | A11031 |  |
|  |  |  |  |  |
|  |  |  |  |  |

- 1. **Cell lines**

| **Name** | **Citation** | **Supplier** | **Cat no.** | **Passage no.** | **Authentication test method** |
| --- | --- | --- | --- | --- | --- |
| HepG2 |  | ECACC | 85011430 | 100; Lot#17K028 | STR |
| Hep3B |  | ECACC | 86062703 | 5; Lot#13C010 | STR |
| Huh7 |  | CLS GmbH | 300156 | 46; Lot#300156-719 | STR |
| AML-12 |  | LGC Standards | ATCC-CRL-2254 | 28;  Lot#70008244 | CoA ATCC |
| Hepa1-6 |  | LGC Standards | ATCC-CRL-1830 | Unknown + 6;  Lot#70018321 | CoA ATCC |

- 1. **Organisms**

| **Name** | **Citation** | **Supplier** | **Strain** | **Sex** | **Age** | **Overall n number** |
| --- | --- | --- | --- | --- | --- | --- |
|  |  |  |  |  |  |  |

- 1. **Sequence based reagents**

| **Name** | **Sequence** | **Supplier** |
| --- | --- | --- |
| iBONI siRNA hRUVBL1 (guide) | 5'-UAGAGAGAGAGAGAGAGAGCCCCC-3' | Riboxx GmbH |
| iBONI siRNA mRUVBL1 (guide) | 5'-AUUAAUUCUACAAUGACGCCCCC-3' | Riboxx GmbH |
| iBONI siRNA negative control N3 (guide) | 5'-ACAUUCAUAUAGCΜGCCCCC-3' | Riboxx GmbH |
| iBONI siRNA mGAPDH | 5’-ACAAUCUCCACUUUGCCACCCCC-3’ | Riboxx GmbH |
| iBONI siRNA hGAPDH | 5’-AUGAGUCCUUCCACGAUACCCCC-3’ | Riboxx GmbH |

- 1. **Biological samples**

| **Description** | **Source** | **Identifier** |
| --- | --- | --- |
|  |  |  |

- 1. **Deposited data**

| **Name of repository** | **Identifier** | **Link** |
| --- | --- | --- |
| PRIDE | PXD075574 DOI: 10.6019/PXD075574 | <https://www.ebi.ac.uk/pride/> |

- 1. **Software**

| **Software name** | **Manufacturer** | **Version** |
| --- | --- | --- |
| Prism | Graphpad | 10 |
| Metaboanalyst | https://www.metaboanalyst.ca/ | 6.0 |
| GEPIA2[1] | http://gepia2.cancer-pku.cn/#index | 2 |
| GENI[2] | https://yoavshaul-lab.shinyapps.io/gsea-geni/ | NA |
| Wave | Agilent Tecnologies | 2.6.1.53 |
| Huygens Professional | Scientific Volume Imaging | 17.0 |
| Fiji | [doi:10.1038/nmeth.2019](https://doi.org/10.1038/nmeth.2019) |  |
| MS Quantitative Analysis | Agilent | 10.2 |
| MaxQuant | <https://doi.org/10.1038/nbt.1511> | 1.6.1.0 |

- 1. **Other (*e.g*. drugs, proteins, vectors etc.)**

| **Description** | **Manufacturer** | **Cat. No.** |
| --- | --- | --- |
| **Cell culture media and reagents** | | |
| HepatoZYME-SFM | Gibco | 17705-021 |
| William’s E Medium | Gibco | 12551032 |
| Glutamax | Gibco | 35050061 |
| Liver perfusion Medium | Gibco | 17701-038 |
| Liver Digest Medium | Gibco | 17703-034 |
| DMEM | Thermo Fisher Scientific | 11960044 |
| MEM | Euroclone | ECB2071L |
| DMEM/F12 | Thermo Fisher Scientific | 11320082 |
| FluoroBrite DMEM | Thermo Fisher Scientific | A1896701 |
| Fœtal Bovine Serum | Euroclone | ECS5000L |
| Hepes | Euroclone | ECM0180L |
| Insulin-Transferrin-Slenium | Gibco | 41400-045 |
| Dexamethasone | Gibco | A13449 |
| CB-6644 | ChemScene | CS-0085306 |
| ADP | Sigma-Aldrich | A2754 |
| Oligomycin | Sigma-Aldrich | O4876 |
| FCCP | Sigma-Aldrich | C2920 |
| Digitonin | Sigma-Aldrich | D5628 |
| Malic acid | Sigma-Aldrich | M7397 |
| Glutammic acid | Sigma-Aldrich | G8415 |
| Bovine Type I collagen solution 0.1% | Sigma-Aldrich | C8919 |
| Pen/Strep solution | Sigma-Aldrich | P0781 |
| LookOut® Mycoplasma PCR Detection Kit | Sigma-Aldrich | MP0035-1KT |
| Fugene HD | Promega | E2311 |
| INTERFERin | Polyplus | 101000028 |
|  |  |  |
| **Molecular biology reagents and kit** |  |  |
| Western blot ECLTM Select. | Cytiva | GERPN2235 |
| Anti-HA magnetic beads | Sigma Aldrich | SAE0197 |
| Seahorse XF Real-Time ATP Rate Assay Kit | Agilent Technologies | 103592-100 |
| Seahorse XF Cell Mito Stress Test Kit | Agilent Technologies | 103015-100 |
| **Vectors** |  |  |
| OMP25-HA | Addgene | 83356 |
| OMP25-Myc | Addgene | 83355 |
| **Fluorescent probes and dyes** | | |
| To-Pro-3 Iodide | Invitrogen | T3605 |
| Mitotracker Deep Red FM | Invitrogen | M22426 |
| Calcein AM | Invitrogen | C3099 |
| JC-1 | Invitrogen | M34152 |
| TMRM | Invitrogen | M20036 |
| Prolong Glass Antifade Mountant | Thermo Fisher Scientific | P36982 |
| **Metabolomics Reagents** | | |
| Methoxamine hydrochloride | Merck | 226904 |
| Pyridine | Merck | 270970 |
| MTBSTFA | Merck | 375934 |
|  |  |  |
|  |  |  |
|  |  |  |

- 1. **Please provide the details of the corresponding methods author for the manuscript:**

| **Tommaso Mello –** Dept. of Clinical and Experimental Biomedical Sciences “Mario Serio”, University of Florence, Florence, Italy  Email: tommaso.mello@unifi.it |
| --- |

**2.0 Please confirm for randomised controlled trials all versions of the clinical protocol are included in the submission. These will be published online as supplementary information.**

|  |
| --- |
