# Supplementary material for: Targeting RuvBL1 disrupts mitochondrial metabolism and structure in hepatocellular carcinoma
Source: JHEP Rep. 2026 Apr 17;8(7):101858. doi: 10.1016/j.jhepr.2026.101858 (PMC13310627; doi:10.1016/j.jhepr.2026.101858)
Supplement: Multimedia component 5 [file mmc5.pdf]

Research article

JHEP|Reports

Targeting RuvBL1 disrupts mitochondrial metabolism and structure in hepatocellular carcinoma

Authors

Tommaso Mello, Irene Simeone, Alice Guida, …, Paolo Pinton, Massimo Bonora, Andrea Galli

Correspondence

tommaso.mello@unifi.it (T. Mello), andrea.galli@unifi.it (I. Simeone).

Graphical abstract

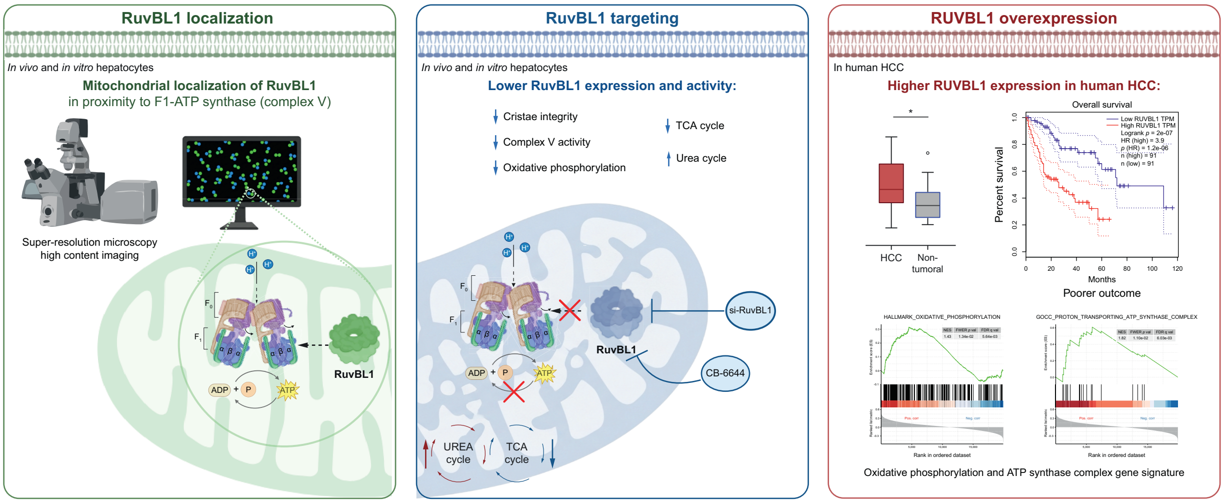

Highlights:

- The AAA+ ATPase RuvBL1 localises to mitochondria and interacts with ATP synthase.
- Targeting RuvBL1 impairs mitochondrial structure and metabolism.
- In human HCC, RUVBL1 expression correlates with mitochondrial oxidative phosphorylation, ATP synthase complex and adverse clinical prognosis.

Impact and implications:

Metabolic reprogramming is a key feature driving HCC onset, progression, and plasticity, contributing to treatment resistance and poor prognosis. RUVBL1 overexpression correlates with reduced survival of patients with HCC and has emerged as a potential metabolic modulator. In this study, we found that targeting RuvBL1 impairs its interaction with mitochondrial ATP synthase, disrupting mitochondrial metabolism and cristae structure. In human HCC samples, RUVBL1 expression correlates with hallmark mitochondrial metabolic processes. These findings may inform the development of targeted therapeutic approaches aimed at impairing the metabolic rewiring and plasticity of HCC.

https://doi.org/10.1016/j.jhepr.2026.101858
© 2026 The Authors. Published by Elsevier B.V. on behalf of European Association for the Study of the Liver (EASL). This is an open access article under the CC BY license (http://creativecommons.org/licenses/by/4.0/).

Targeting RuvBL1 disrupts mitochondrial metabolism and structure in hepatocellular carcinoma

Tommaso Mello[1,*,†], **Irene Simeone**[1,†], Alice Guida[1], Dimitri Papini[1], Francesca Begnozzi[2], Alice Santi[1], Daniele Guasti[3], Patrizia Nardini[3], Simone Polvani[1], Matteo Lulli[1], Oxana Bereshchenko[4], Elisabetta Ceni[1], Armando Curto[1], Paolo Pinton[2,5], Massimo Bonora[2], Andrea Galli[1,*]

JHEP Reports **2026**. vol. 8 | 1–14

Check for updates

Background & Aims: The AAA+ ATPase RuvBL1 takes part in several biological processes, including chromatin remodelling and DNA repair, ribosome biogenesis, mTOR signalling, and oncogenic transformation. RUVBL1 overexpression correlates with poor survival in patients with hepatocellular carcinoma (HCC). We previously found that RuvBL1 is a key regulator of liver glucose metabolism in mice. Here, we aimed at disentangling the metabolic function of RuvBL1 in HCC cells.

Methods: Non-transformed AML-12, primary mouse hepatocytes, HCC cell lines, and RuvBL1$^{hep-/-}$ mice were used (n = 3). RuvBL1 was targeted by RNAi and by inhibition with CB-6644. Metabolomic profiling and mitochondrial functions were assessed by targeted GC/MS, Seahorse analysis, and ATP synthase activity. Mitochondrial morphology and membrane potential were investigated by fluorescence microscopy, high-content imaging, and transmission electron microscopy. Mitochondrial RuvBL1 was detected by WB, super-resolution microscopy, transmission electron microscopy, and proximity ligation assay. Human HCC and normal liver samples from The Cancer Genome Atlas and GTEx databases were used for *in-silico* analysis (T = 369, N = 160).

Results: Targeting RuvBL1 impairs mitochondria-centred metabolic processes, including amino acid metabolism, TCA cycle, and oxidative phosphorylation. Inhibition of RuvBL1/2 activity induces loss of cristae integrity, mitochondrial hyperpolarisation and fragmentation, a phenotype paralleled by the hepatocytes of RuvBL1$^{hep-/-}$ mice. We detected RuvBL1 in proximity to mitochondrial ATP synthase, a previously unreported localisation for this protein. Mechanistically, CB-6644 reduces ATP synthase-RuvBL1 interaction and impairs complex V activity even under a fuelled TCA cycle. In human HCC, higher RUVBL1 expression correlates with gene signatures associated with mitochondrial oxidative phosphorylation (FDR = 5.64e^{-03}), ATP synthase complex (FDR = 6.03e^{-03}), and poorer outcome (p = 2e^{-07}).

Conclusions: Targeting RuvBL1 impairs complex V activity, disrupting mitochondrial metabolic functions and structural integrity. The mitochondrial functions of RuvBL1 may inform novel therapeutic strategies in the fight against hepatocellular carcinoma.

© 2026 The Authors. Published by Elsevier B.V. on behalf of European Association for the Study of the Liver (EASL). This is an open access article under the CC BY license (http://creativecommons.org/licenses/by/4.0/).

Introduction

RuvBL1 (Pontin52) is an AAA+ ATPase involved in multiple cellular activities, including chromatin remodelling, telomere maintenance,[1] ribosome biogenesis, and oncogenic transformation.[2–4] Overexpression of RUVBL1 and of the closely related RUVBL2 genes occurs frequently in human cancers,[5–10] including hepatocellular carcinoma,[11,12] and their higher expression correlates with reduced survival.

RuvBLs are increasingly drawing attention as potential targets for cancer treatment,[13–15] and the recently developed small-molecule inhibitor CB-6644, which targets RuvBL1/2 ATPase activity, showed promising results in a large panel of cancer cell lines.[16]

A notable function of RuvBL1/2 is to promote the expression of mTOR[17] as well as the assembly of mTORC1 and mTORC2 complexes.[18] Mechanistically, RuvBL1/2 are key components of the HSP90 co-chaperone complex R2TP, which, together with the TTT-chaperone, assists in the co-translational folding of mTOR and maturation of the mTORC1 complex.[18–20] In yeast, the R2TP complex participates in nutrient sensing, coordinating cell growth with nutrient availability.[21] We have previously shown that the haploinsufficiency of RUVBL1 in mouse liver impairs mTOR expression and results in a metabolic phenotype characterised by hepatic insulin resistance, increased glucose output, hyperglycaemia, and increased blood triglycerides and cholesterol levels.[12] Recently, Chen *et al.*[22] uncovered HPCAL1 as a negative regulator of hepatocellular carcinoma (HCC) growth *in vivo,* which acts by binding to RuvBL1 and disrupting the TTT-RuvBL-mTORC1 interaction. Collectively, RuvBL1 and RuvBL2 are emerging as potent metabolic modulators and potential therapeutic targets for anticancer strategies.[12,16,20]

Hepatocellular carcinoma (HCC) is a multifaceted entity arising on top of different underlying liver conditions, aetiologies, and molecular drivers. Despite these diversities,

* Corresponding authors. Address: Gastroenterology Unit, Viale Pieraccini 6, 50139 Florence, Italy.
E-mail addresses: tommaso.mello@unifi.it (T. Mello), andrea.galli@unifi.it (I. Simeone).
† Equally contributed as joint first authors.
https://doi.org/10.1016/j.jhepr.2026.101858

metabolic reprogramming emerges as a shared characteristic that drives virtually any phase of HCC development, from onset to progression, resistance, and immune escape, offering potential leverage for precision medicine approaches, from diagnosis to treatment.[23–26]

In this work, we aimed to deepen the understanding of RuvBL1's metabolic role in HCC. We found that RuvBL1 is required for proper mitochondrial respiration and that targeting RuvBL1/2 activity results in a peculiar mitochondrial damage characterised by membrane hyperpolarisation, swelling, and loss of cristae integrity. We detected RuvBL1 into the mitochondria, a previously unreported localisation for this protein. Mechanistically, RuvBL1 and ATP synthase (complex V) are found in proximity, and treatment with CB-6644 reduces their interaction, impairing mitochondrial ATP production.

RUVBL1 expression in human HCC samples correlates with the expression of (F_1)-ATP synthase subunits and metabolic genes involved in both glycolysis and oxidative phosphorylation (OXPHOS), with mTOR pathway activation, with a more advanced tumour stage and with reduced survival.

These results suggest that RuvBL1 overexpression in HCC is required to meet the increased metabolic demands of cancer cells, supporting mitochondrial activity for ATP generation and biosynthesis.

Materials and methods

Please refer to the supplementary material and supplementary CTAT table for a complete materials and methods description.

Animal model

All procedures involving laboratory animals were conducted in accordance with institutional ethical norms and national laws, after approval by the Italian Ministry of Health (decrees 30/2013 and 665/2018). The conditional knockout RuvBL1$^{hep-/-}$ mice were created by crossing RuvBL1$^{floxed/floxed}$ (RuvBL1$^{f/f}$) mice[27] with Albumin-Cre$^{tg/tg}$ mice acquired from Jackson Laboratories through the local distributor (Charles River Italia). The first round of breeding generated RuvBL1$^{hep+/-12}$ offspring, which were crossed again to produce RuvBL1$^{hep-/-}$ mice. Three male mice per genotype were used for the transmission electron microscopy analysis in this study.

Cell lines and primary hepatocytes

Authenticated Hepa1-6, Huh7, HepG2, Hep3B, and AML-12 cell lines were obtained from suppliers reported in the supplementary CTAT table. Upon arrival and at regular intervals thereafter, all cell lines were tested for *Mycoplasma* by PCR. All cell lines were maintained in culture without antibiotics. Primary hepatocytes were isolated from 3-month-old C57BL/6 mice by collagenase-dispase perfusion and Percoll gradient centrifugation. Primary hepatocytes were maintained on collagen-coated plates and layered with collagen I for sandwich culture.

RUVBL1 gene silencing was performed with 20 nM IBONI siRNA (RiboxX GmbH, Dresden, Germany) or with 5 nM Silencer Select validated siRNAs (Life Technologies Italia, Monza, Italy) using negative-control siRNA and GAPDH siRNA to evaluate the silencing efficiency and the transfection efficiency, respectively. INTERFERin (Polyplus-Sartorius, Göttingen, Germany) or RNAiMAX (Life Technologies Italia, Monza, Italy) were used as transfection reagents. RUVBL1/2 ATPase activity was targeted with the specific inhibitor CB-6644 (ChemScene, USA).

Metabolomic analysis

Targeted gas chromatography-mass spectroscopy (GC-MS) metabolomics was performed on Huh7 cells cultured in complete medium and treated for 48 h with 0.5 μM of CB-6644 or vehicle alone. Metabolites were extracted with 80% cold methanol supplemented with 1% norvaline as internal standard. Dried extracts were derivatised in 10 μl of 40 mg/ml methoxamine hydrochloride in pyridine at 37 °C for 90 min, followed by 50 μl of MTBSTFA (Sigma-Adrich, MERCK, Italy) at 60 °C for 30 min. Data acquisition was performed using an Intuvo 9000 GC/5977B MS System (Agilent Technologies Italy) equipped with an HP-5MS capillary column (30 m × 0.25 mmx0.25 μm). For relative metabolite abundances, the peak area of each metabolite was normalised to norvaline and to protein concentration.

Complex V activity

Complex V activity was assessed in Huh7 cells transiently transfected with a mitochondrially targeted luciferase chimera (mtLuc). After treatment with CB-6644, cells were incubated in a thermostated perfusion chamber, and real-time luminescence emission was recorded using a custom-built luminometer. Recordings were initiated with a 30-s baseline measurement in intracellular buffer (IB) designed to mimic the cytosolic ionic composition. Cells were then perfused with IB containing 25 μM luciferin (IBluc) to stabilise ATP-driven luciferase light production. After plasma membrane permeabilisation with 25 μM digitonin (Sigma-Aldrich, MERCK, Italy), cells were sequentially exposed to IBluc supplemented with 1 mM malic acid and 1 mM glutamic acid (Sigma-Aldrich, MERCK, Italy), followed by 5 mM ADP (Sigma-Aldrich, MERCK, Italy). The resulting increase in luminescence, measured in counts per second, reflected ATP synthesis driven by complex V activity in response to exogenous ADP.

In-silico analysis

RUVBL1 expression in normal liver (The Cancer Genome Atlas [TCGA] and GTEx) and HCC samples of the TCGA_liver hepatocellular carcinoma (LIHC) cohort was evaluated through the GEPIA2 web tool, last accessed on 18 January 2026. Overall survival and most differential survival genes analyses in the LIHC cohort were performed in GEPIA2 using RUVBL1 expression quartiles (75–25%) as cut-off values for group definition. RUVBL1 expression levels across HCC stages were also graphed in GEPIA2.

Gene Set Enrichment Analysis (GSEA) on the LIHC cohort was performed through the web app GENI, using Spearman's correlation method and default settings. The TCGA database was accessed via cBioPortal for Cancer Genomics to retrieve RuvBL1 mRNA expression data in the LIHC cohort. Based on RUVBL1 expression, patients were assigned to the HI_RUVBL1 (Z-score >2) or LOW_RUVBL1 (Z-score <2) group. The mRNA expression data of genes differentially expressed

between the two groups was used to run a Gene Ontology (GO) analysis with ClueGO (Cytoscape app).

Statistical analysis

Statistical analyses were performed with GraphPad Prism 10 (GraphPad Software, San Diego, CA, USA) on data from three or more replicates. The statistical significance levels achieved, and the types of tests used are reported in figure legends using standard notations: *p <0.05, **p <0.01, ***p <0.001, and ****p <0.0001. Exact p values for each analysis are reported in the Supplementary materials.

Results

RuvBL1/2 ATPase activity is required for mitochondrial metabolism

To determine the extent of RuvBLs-regulated metabolic processes in human HCC cells, we conducted targeted metabolomic analysis by GC-MS in Huh7 cells treated with CB-6644 (0.5 μM for 48 h), a selective inhibitor of the RuvBL1/2 ATPase complex.[16] The doses and duration of CB-6644 treatment used in this work were chosen based on published ranges[16] and after preliminary verification that no significant cell death was occurring. Several key metabolites along the glycolytic and TCA cycle pathways, such as PEP, pyruvate, lactate, and virtually all TCA cycle intermediates, were significantly reduced by CB-6644 as shown in Fig. 1A, B. The levels of most of the analysed amino acids were also significantly decreased, with the noticeable exception of aspartate and asparagine, whose levels were clearly increased by CB-6644 administration (Fig. 1A, B).

Quantitative enrichment analysis performed with Metaboanalyst allowed a clear separation of CB-6644 treated cells *vs* controls by PCA (Fig. S1A), and identified several significantly enriched pathways, including the urea cycle, malate–aspartate shuttle, Warburg effect, and amino acids metabolism (Fig. 1C; Fig. S1B). Functional annotation of the CB-6644-modulated metabolite set using the Consensus Path Database[28] (within KEGG, Reactome, and WikiPathways), confirmed significant enrichment in pathways involved in the regulation of energy metabolism and cancer metabolic reprogramming, as well as cytosolic and mitochondrial translation-related processes (Table S1). Interestingly, several of the most enriched pathways, such as the urea cycle, amino acids metabolism, and TCA cycle, are centred in or converge on mitochondria, suggesting that this organelle could be pivotal in the metabolic action mediated by RuvBL1/2 ATPase activity.

RuvBL1 is required for OXPHOS

RuvBL1 was knocked down in murine and human HCC cells (Hepa1-6, HepG2, Hep3B, and Huh7), in a non-tumoural hepatocytic cell line (murine AML-12) and the impact on cellular respiration was measured by the Seahorse MitoStress test (Fig. 2A, B; Fig. S2). Reducing RuvBL1 protein level affected oxidative phosphorylation (OXPHOS) in all tested cell lines, clearly hampering their basal respiratory capacity (Fig. 2; Fig. S2). We next investigated whether the ATPase activity of the RuvBL1/2 complex is required for mitochondrial respiration. The abovementioned cell lines and freshly isolated mouse hepatocytes were treated for 24–72 h with increasing doses of CB-6644 (0.25–1 μM). The basal respiratory capacity was affected in a time- and dose-dependent manner, with higher doses inhibiting OXPHOS after 24 h and lower doses becoming effective after 48 h of treatment (Fig. 3A; Fig. S3A and C).

The reduced OXPHOS in CB-6644-treated cells was mirrored by a significant reduction of the mitochondrial ATP production, which was not compensated for by a concomitant increase in glycolysis, as assessed by the Seahorse ATP-rate assay (Fig. 3B and Fig. S3B and D). Intriguingly, inhibition of RuvBL1/2 activity also reduced ATP production through glycolysis in the cancer cell lines but not in non-transformed AML-12 cells and in primary hepatocytes (Fig. 3B and Fig. S3B and D, purple bars).

We then hypothesised that the reduced respiratory capacity of CB-6644-treated and RuvBL1-silenced cells could stem from a reduction in mitochondrial content or activity, the latter being commonly associated with a reduced mitochondrial membrane potential ($\Delta\Psi$m).

Treatment with CB-6644 mildly decreases Mitotracker staining intensity, suggesting a reduction in the total mitochondrial mass (Fig. S4A). Although statistically significant, this reduction was relatively modest (5–10% depending on dose and cell line) hardly accounting for the robust loss of the cell respiratory capacity (Fig. 3A). Contrary to our expectation, the $\Delta\Psi$m indicator JC-1 surprisingly revealed that treatment with CB-6644 induces the hyperpolarisation of mitochondria (increased negative charge across the membrane), a type of disfunction known to precede mitochondrial fragmentation[29] (Fig. S4B). These data were further corroborated by high-content imaging analysis of AML-12 and Huh7 cells stained with the mitochondrial dye tetramethyl rhodamine methyl ester (TMRM), which accumulates in active mitochondria in a voltage-dependent manner. Consistent with the JC-1 results, CB-6644 dose- and time-dependently increased the average fluorescence intensity of TMRM-stained AML-12 and Huh7 cells, strongly suggesting an increase of the $\Delta\Psi$m (Fig. S4C and D). Oligomycin inhibits the ATP synthase activity, preventing proton re-entry through complex V and typically inducing a transient hyperpolarisation in coupled mitochondria. As expected, oligomycin treatment caused a marked increase in $\Delta\Psi$m in vehicle-treated AML-12 cells (Fig. S4E). In contrast, CB-6644–treated cells exhibited an elevated basal $\Delta\Psi$m and showed little to no further increase upon oligomycin addition, indicating a reduced oligomycin-sensitive proton flux through ATP synthase. Together, these results indicate that targeting RuvBL1 reduces mitochondrial oxidative phosphorylation, which is accompanied by mitochondrial hyperpolarisation and a decrease in mitochondrial mass.

RuvBL1 is localised to mitochondria, and it is required for their integrity

Staining with Mitotracker and TMRM clearly revealed that CB-6644 treatment alters mitochondrial morphology. Fig. 4 shows the heterogeneity of mitochondrial shapes observed in primary mouse hepatocytes and cell lines treated with 0.5 μM CB-6644 for 48 h. Although mitochondria are elongated and uniformly shaped in vehicle-treated cells, they become swollen or

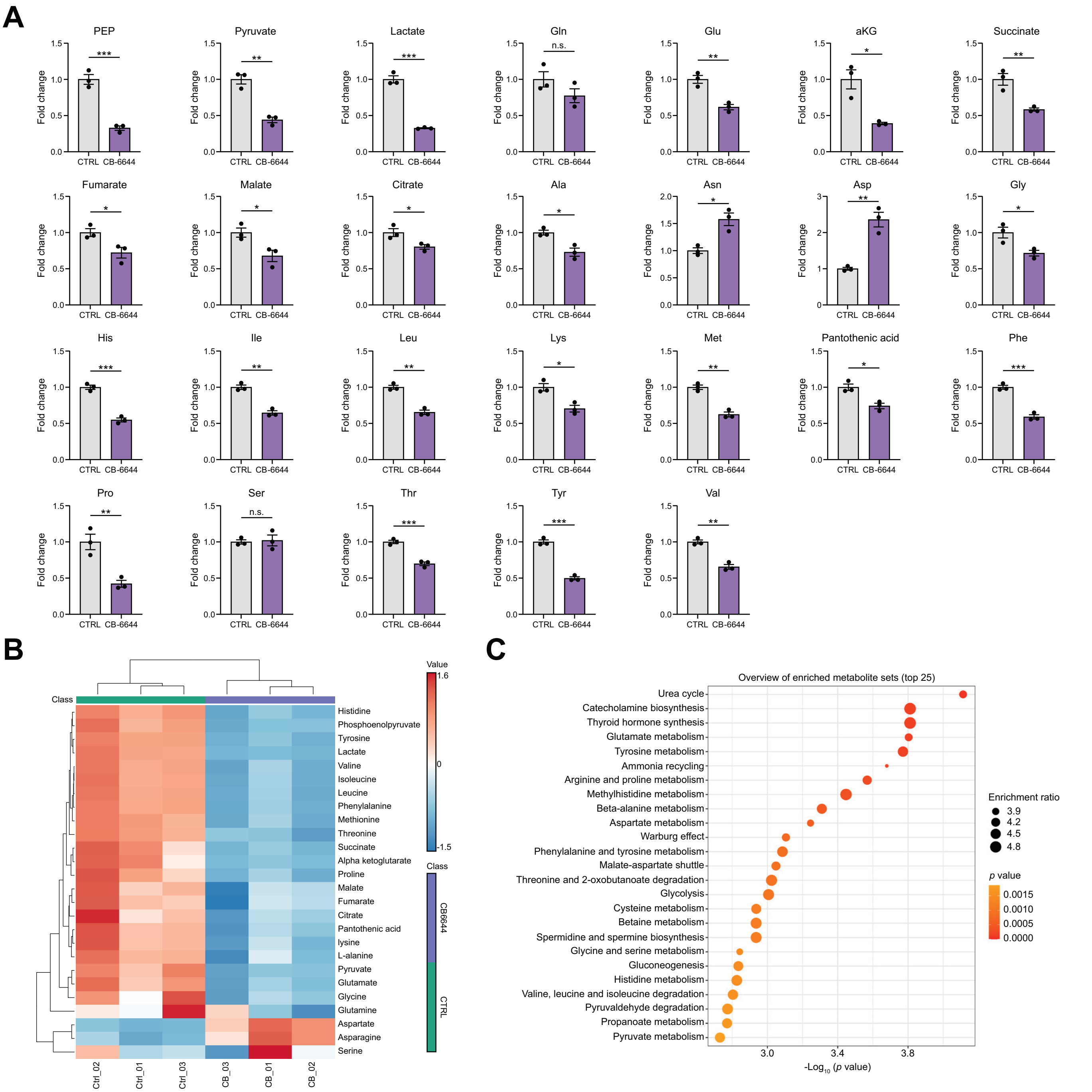

Fig. 1. Targeting RuvBL1/2 ATPase activity impairs cellular metabolism. (A) Targeted metabolomic analysis of Huh7 cells treated with CB-6644 0.5 μM for 48 h. Normalised AUC values (mean ± SEM) from three independent experiments are reported. Unpaired Student's *t* test was used for statistical significance. $^{*}p < 0.05$, $^{**}p < 0.01$, $^{***}p < 0.001$. (B) Hierarchical clustering of the analysed metabolites in CB-6644-treated Huh7 cells. (C) Enrichment analysis performed with Metaboanalyst in CB-6644-treated Huh7 cells.

fragmented in CB-6644-treated cells. Quantitative assessment of the mitochondrial network integrity, performed by high-content imaging in TOMM20-stained AML-12 and Huh7 cell lines, confirmed that inhibition of RuvBL1/2 ATPase activity results in the progressive fragmentation of the mitochondrial network (Fig. 4C).

Assessment of the mitochondrial structure by transmission electron microscopy (TEM) revealed that even the lower dose of CB-6644 (0.25 μM) impacts mitochondrial morphology after 48 h in all the tested cell lines, causing the loss of matrix electron density and the disruption of mitochondrial cristae (Fig. S5). We further investigated this intriguing aspect by a detailed morphometric analysis of Huh7 mitochondria by TEM. As shown in Fig. 5, treatment with CB-6644 induces the swelling of mitochondrial cristae in a dose- and time-dependent manner.

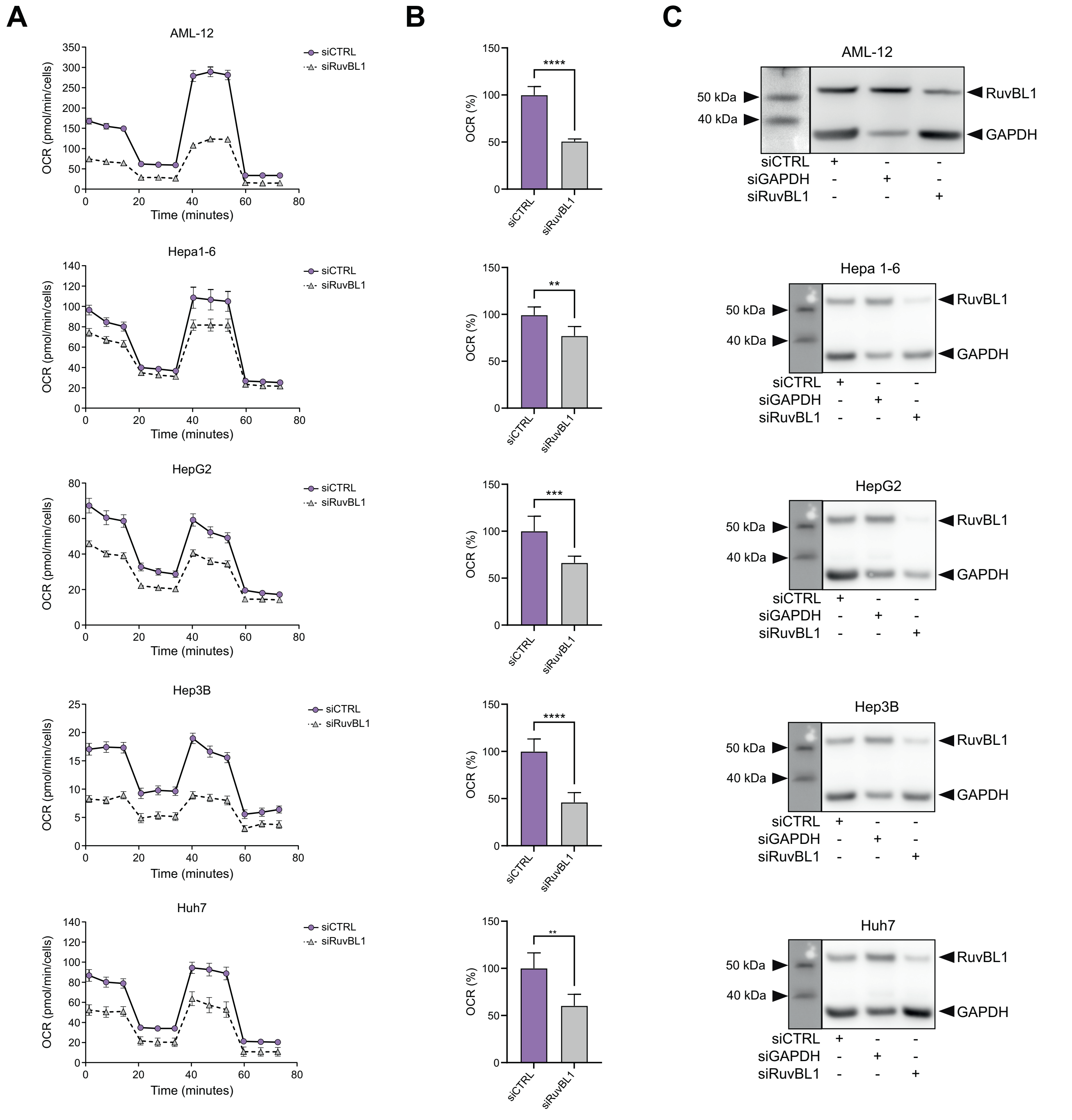

Fig. 2. RuvBL1 knockdown impairs mitochondrial respiration. (A) Seahorse MitoStress test in RuvBL1-silenced cells. Representative normalised Oxygen Comsumption Rate (OCR) profiles (mean ± SEM). (B) Quantification of the basal respiratory capacity in RuvBL1-silenced cells. Values are scaled to the siCTRL average and presented as mean ± SD of at least three independent experiments. Unpaired Student's *t* test was used for statistical significance. *p <0.05, **p <0.01, ***p <0.001, ****p <0.0001. (C) RuvBL1 expression in control and RuvBL1-silenced cells. Representative WB for each cell line, transfected with scramble siRNA, siGAPDH (to monitor transfection efficiency), and siRUVBL1.

Notably, a similar phenotype was observed in liver samples from RuvBL1$^{hep-/-}$ mice, whose hepatocytes show reduced mitochondrial density and strikingly fewer mitochondrial cristae, compared to RuvBL1$^{f/f}$ control mice (Fig. 5B). Notably, RuvBL1$^{hep-/-}$ mice, but not RuvBL1$^{f/f}$ or RuvBL1$^{hep+/-}$ ones,[12] present hepatocellular damage, high apoptosis and proliferation indexes, suggestive of chronic liver damage and regeneration (manuscript in preparation).

The evident *in vitro* and *in vivo* mitochondrial phenotype induced by RuvBL1 targeting prompted us to investigate a yet

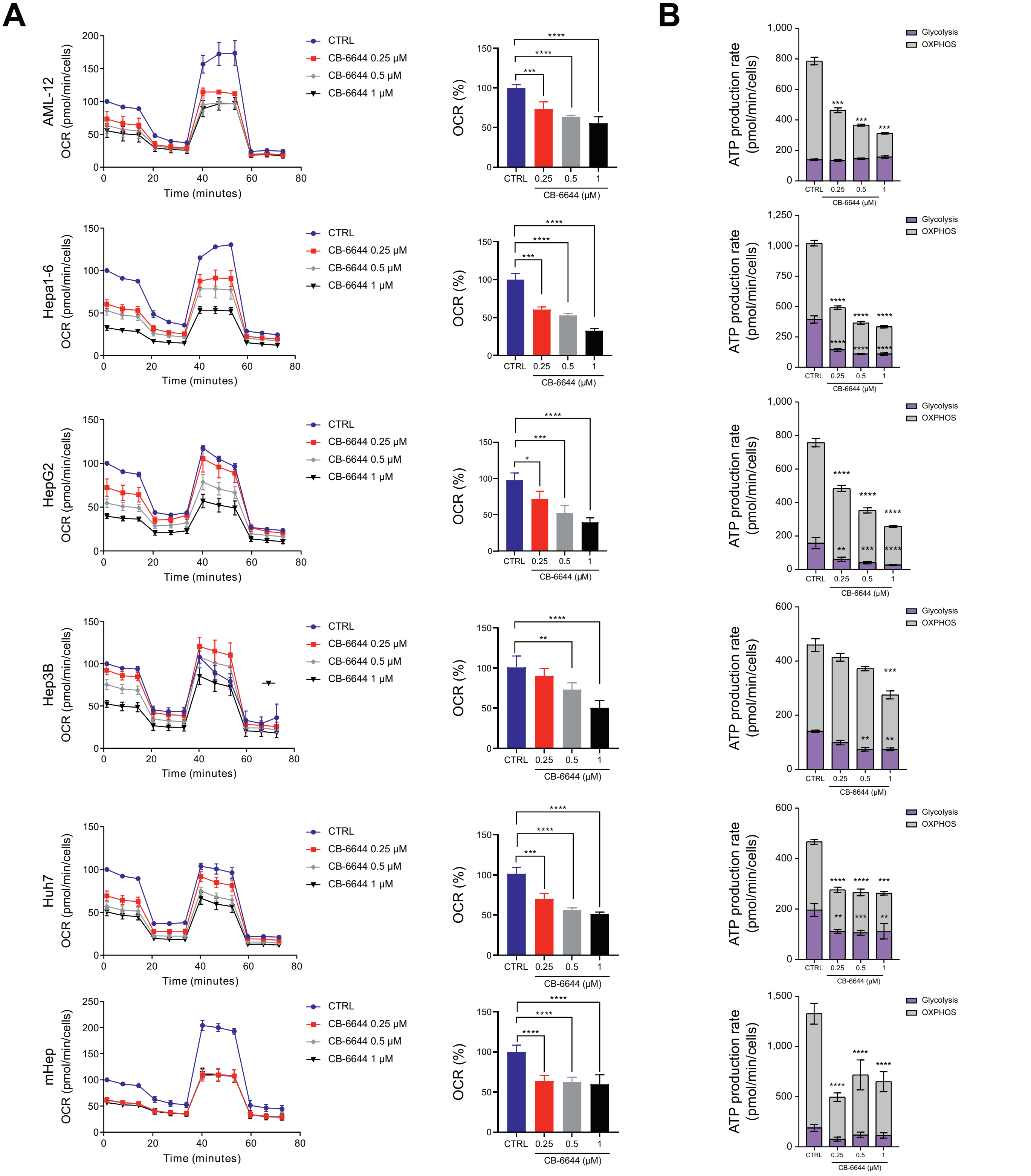

Fig. 3. Inhibition of RuvBL1/2 ATPase activity impairs OXPHOS and ATP production. (A) Line graphs: Seahorse MitoStress test profiles of cell lines and primary mouse hepatocytes treated with CB-6644 for 72 h (mean ± SEM, n = 3–5 independent experiments). OCR values are normalised by cell number and scaled relative to the basal OCR of non-treated cells. Bar-graph: Quantification of the basal respiratory capacity shown in panel A (mean ± SD). Statistical significance was calculated by one-way ANOVA with Dunnett's correction for multiple comparisons. **p* <0.05, ***p* <0.01, ****p* <0.001, *****p* <0.0001. (B) ATP-rate assay showing the relative contribution of glycolysis and OXPHOS to the total ATP production in cells treated with CB-6644 for 72 h (mean ± SEM, *n* = 3). Statistical significance (*vs.* control) was calculated by two-way ANOVA with Dunnett's correction for multiple comparison. **p* <0.05, ***p* <0.01, ****p* <0.001, *****p* <0.0001. OXPHOS, oxidative phosphorylation.

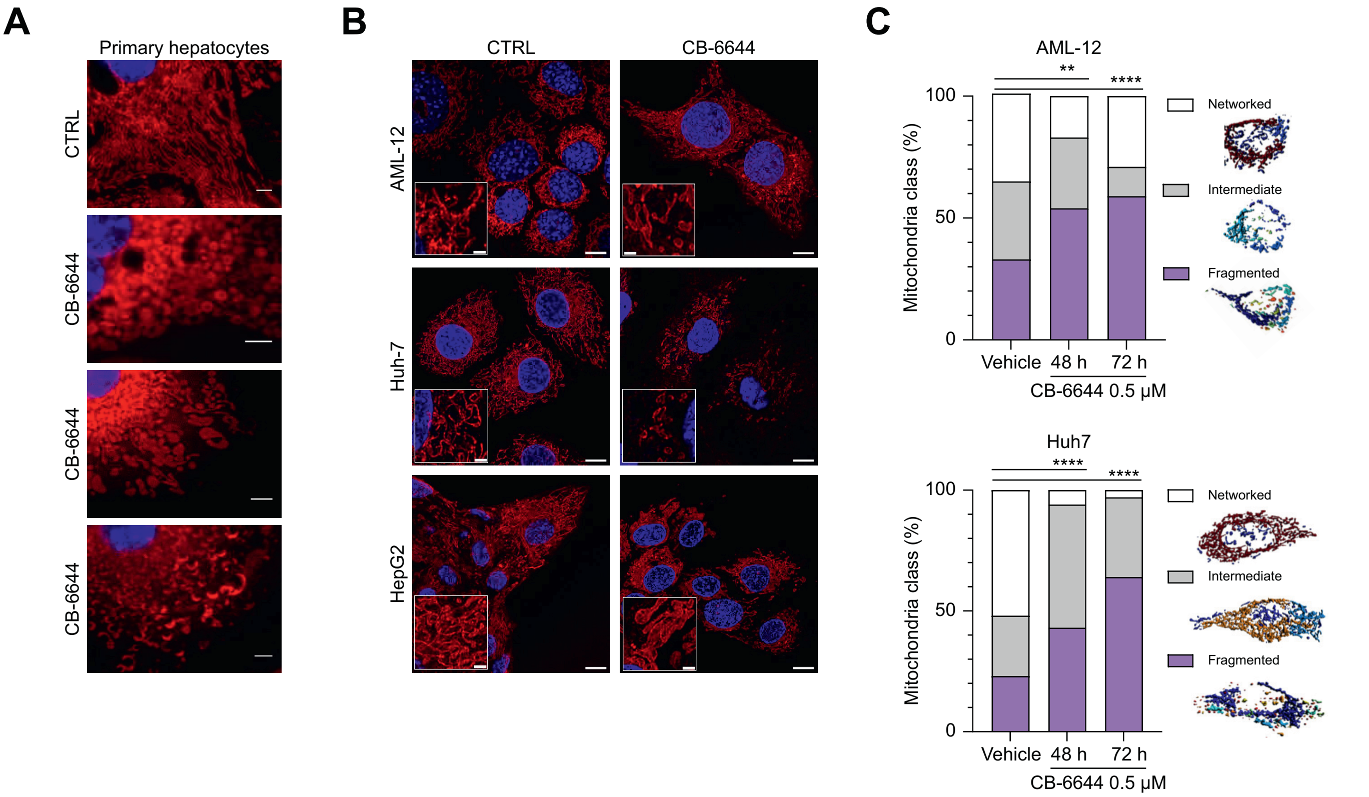

Fig. 4. CB-6644 induces mitochondrial swelling and fragmentation. (A) Primary mouse hepatocytes treated with 0.5 μM CB-6644 for 48 h. Widefield images, scale bar = 5 μm. (B) Cell lines treated with 0.5 μM CB-6644 for 48 h. Confocal images, scale bar = 10 μm. Inset scale bar = 2 μm. (C) Classification of the mitochondrial network structure in AML12 and Huh7 exposed to 0.5 μM CB-6644 for 48 h. Cells were immunostained for the mitochondrial marker TOMM20 and imaged by high-content confocal imaging. Representative isosurface rendering are also shown (right panel). Isosurface rendering was false-coloured to represent the volume of each mitochondrial particle. Statistical significance (*vs*. CTRL) was calculated by Fisher's exact test: **p* <0.05, ***p* <0.01, ****p* <0.001, and *****p* <0.0001.

unreported mitochondrial localisation of RuvBL1. Indeed, super-resolution STED microscopy imaging strongly suggested that RuvBL1 localises in proximity and within mitochondria in the tested cell lines (Fig. 6A). Immunogold labelling and TEM consolidated the mitochondrial localisation of RuvBL1 in all tested cell lines as well as in murine liver sections (Fig. 6B). Furthermore, cellular fractionation experiments confirmed the enrichment of RuvBL1 in mitochondria purified by ultracentrifugation (Fig. S6A).

Inhibition of RuvBL1/2 activity affects the mitochondrial proteome and impairs ATP synthase activity

We next focused on the potential mechanisms underlying the disruption of mitochondrial respiration and cristae structure after CB-6644 treatment. We first assessed the integrity of the electron transport chain (ETC) by analysing the expression of key subunits that are degraded upon disassembly of the ETC complexes. Treatment with CB-6644 for 72 h caused a dose–response reduction of MTCO1 (complex IV) and NDUFB8 (complex I) subunits in Huh7 cells, suggesting a detrimental effect on the stability of these ETC complexes (Fig. 7A).

The mitochondrial cristae structure is shaped and stabilised by several factors, including the ATP synthase complex at the cristae tips and the MICOS complex and OPA1 tethering at the cristae junctions.[30,31]

The expression of MICOS complex subunits Mic60 (IMMT) and Mic19 (CHCHD3) was reduced after 72 h of treatment with CB-6644 in Huh7 cells (Fig. 7A). Full-length OPA1 protein is processed by cellular proteases Oma1 and Yme1l1 to produce several long and short forms, which act as key regulators of the inner mitochondrial membrane (IMM) dynamics during fission/fusion events and cristae remodelling.[32] Consistent with the change in mitochondria morphology, treatment of Huh7 cells with CB-6644 resulted in a shift in the abundance of OPA1 fragments with a relative increase of the short forms, a pattern partially overlapping with that generated by oligomycin, which has been shown to induce OPA1 cleavage and mitochondrial fragmentation[33] (Fig. S6B).

We reasoned that the disruption of the mitochondrial structure caused by CB-6644 would be mirrored by informative modifications of the mitochondrial proteome. To rapidly isolate mitochondria from control and CB-6644-treated cells for subsequent MS analysis, we generated stable Huh7 clones expressing HA-OMP25-EGFP for IP, and FLAG-OMP25-EGFP as negative controls.[34]

Quantitative IP/MS analysis of the mitochondrial proteome identified eight proteins consistently present in control samples only (*bona fide* downregulated by CB-6644), 13 proteins consistently identified only in treated samples (*bona fide* upregulated by CB-6644), and five proteins which were

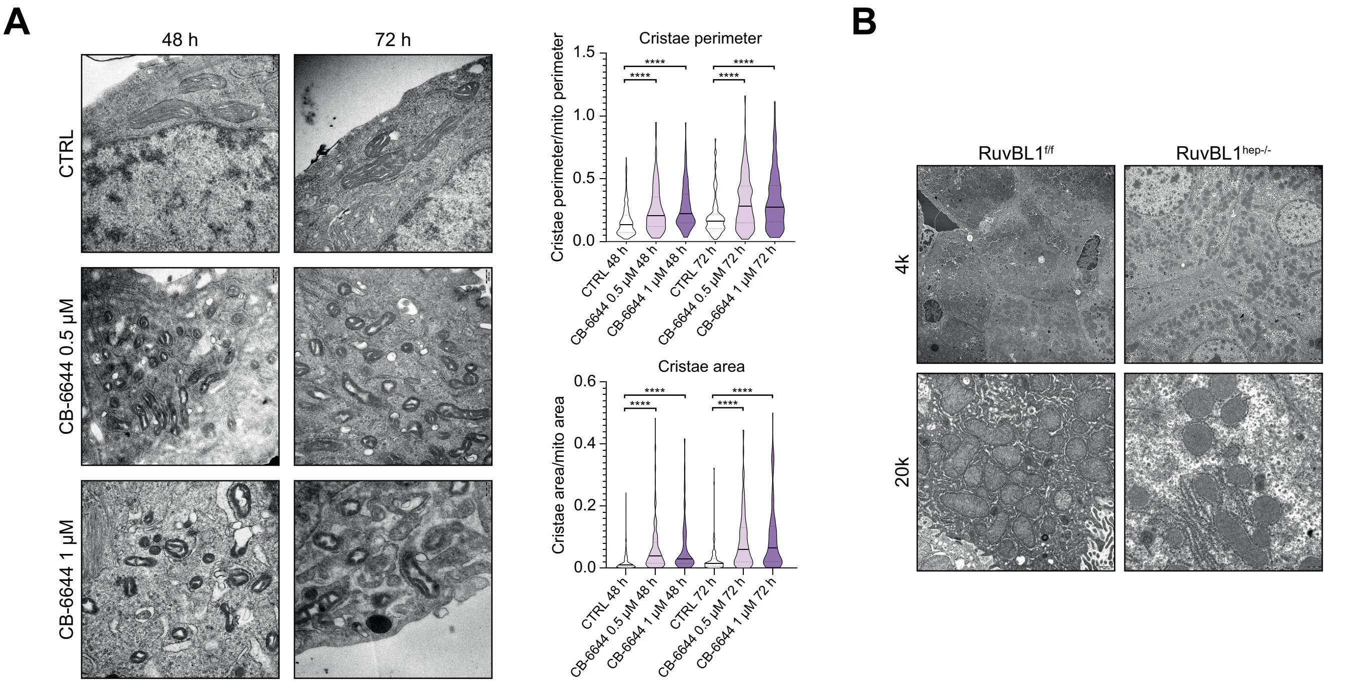

Fig. 5. CB-6644 disrupts mitochondrial cristae structure. (A) TEM morphometric analysis of mitochondrial cristae in Huh7 cells treated with 0.5–1 μM CB-6644 for 48–72 h. Statistical significance (*vs.* time-matched CTRL) was calculated by Kruskal–Wallis with Dunn's correction for multiple comparison. **p* <0.05, ***p* <0.01, ****p* <0.001, and *****p* <0.0001. (B) Liver samples of RuvBL1$^{f/f}$ and RuvBL1$^{hep-/-}$ mice imaged by TEM at 4,000 × and 20,000 × magnification.

identified in all samples, but whose expression was significantly upregulated in CB-6644-treated samples (Fig. S7).

Interestingly, among these modulated proteins, several are related to pyruvate metabolism and TCA (PDP2, D2HGDH, OGDHL, HAGH), lipid metabolism (MECR, AMACR, MLYCD), mitochondrial translation (MRPL52, ALKBH1), iron-sulphur cluster assembly and electron transport (ISCA2, IBA57, CISD3, COQ8B, PPTC7), leucine degradation and ketogenesis

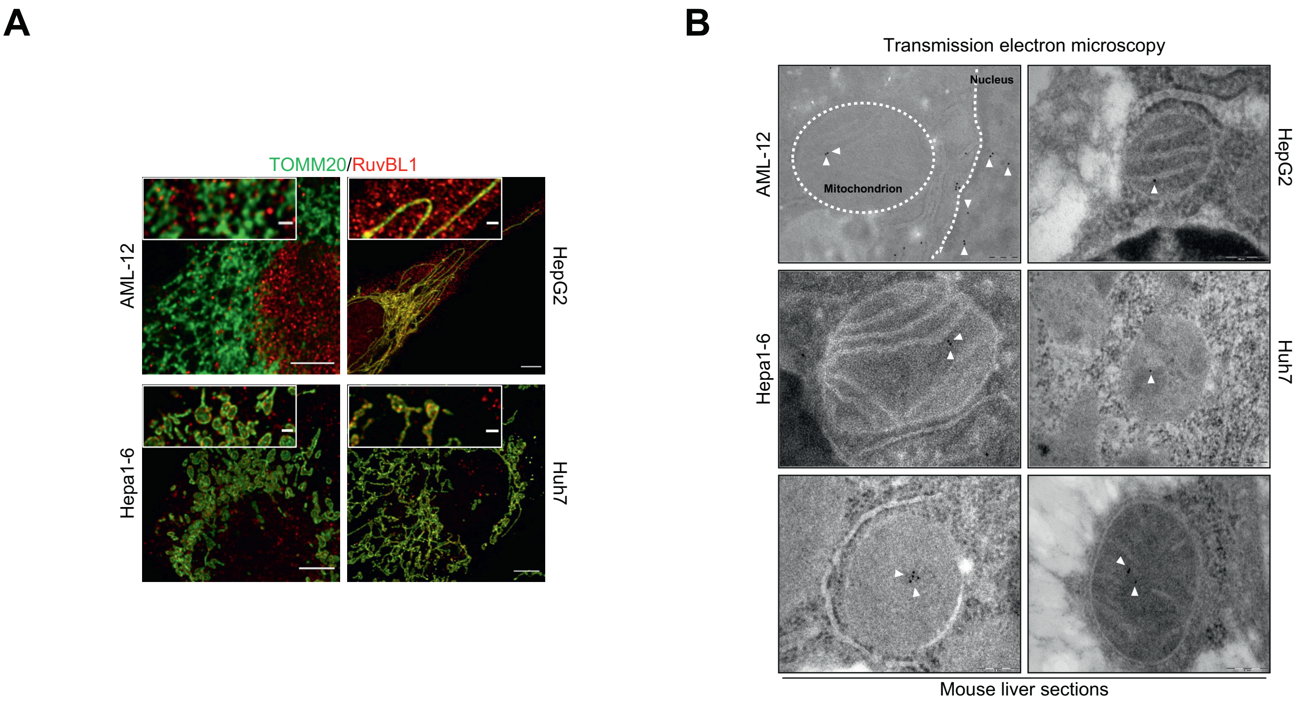

Fig. 6. RuvBL1 is partially localised to mitochondria. (A) Deconvoluted super-resolution STED images of RuvBL1 (red) and TOMM20 (green). Images are presented as maximum intensity projection of z-stacks. Scale bar = 5 μm. Insets: enlarged details with scale bar = 1 um. (B) Immunogold TEM showing RuvBL1 localisation within mitochondria (arrowheads). TEM, transmission electron microscopy.

(IVD, HMGCL), and to membrane transporters involved in the maintenance of the mitochondrial membrane potential (SLC35F6, MCUR1, ATP synthase mitochondrial F1 complex Assembly Factor 2 [ATPAF2]). All these processes and functions appear to be consistent with the metabolic data and mitochondrial phenotype described so far.

ATPAF2 drew our attention as its upregulation by CB-6644 is suggestive of a RuvBL1/2-mediated mechanism that converges on complex V. We first validated the proteomic data by western blotting (WB), confirming that CB-6644 treatment upregulates ATPAF2 protein level in immunoprecipitated mitochondria of Huh7 cells (Fig. S6C). Next, we evaluated the expression of specific ATP synthase subunits essential for the function and stability of the complex, particularly α and β subunits (F1, catalytic portion), γ subunit (central stalk), and d, g, and e subunits (peripheral stalk) (Fig. 7B). Intriguingly, several ATP synthase subunits were upregulated by CB-6644, paralleling ATPAF2 expression and in line with ATP5A data obtained from the total cell lysates (Fig. 7A). Collectively, these data suggest that complex V assembly is not impaired by CB-6644. Nevertheless, the mitochondrial ATP production, measured in digitonin-permeabilised Huh7 under fuelled TCA cycle and in the presence of exogenous ADP, was clearly reduced by CB-6644 (Fig. 7C; Fig. S6D). Interestingly, the expression of ATPIF1, the main inhibitor of complex V activity, was found upregulated by CB-6644 in a dose-dependent manner (Fig. 7A, B).

Wondering how RuvBL1/2 inhibition could result in reduced ATP synthase activity, we looked for potential interaction between these two complexes. Super-resolution STED and STORM microscopy, performed with different antibody pairs, clearly localises RuvBL1 in proximity with (F_1)-ATP synthase (alpha and beta subunits, Fig. 7D; Fig. S6E). Proximity ligation assay (PLA), which detects proteins interacting within a 40 nm range, confirmed the association of RuvBL1 with the (F_1)-ATP synthase complex (Fig. 7E). Quantification of the PLA signal by high-content imaging microscopy revealed that CB-6644 induces a moderate but consistent reduction of the RuvBL1-ATPB interaction (Fig. 7E). Taken together, these data demonstrate that inhibition of RuvBL1/2 affects complex V ATP synthase activity by possibly disrupting the interaction between these two complexes, leading to mitochondrial membrane hyperpolarisation and, eventually, to mitochondrial damage.

In-silico analysis highlights a significant correlation between mitochondrial-related processes and RUVBL1 expression in human HCC

Finally, we explored the relevance of our findings to human HCC, leveraging the publicly available TCGA and GTEx databases. RUVBL1 is overexpressed in the TCGA-LIHC cohort compared with matched TCGA and GTEx normal liver samples, confirming a previous report,[11] and its higher expression correlates with a more advanced stage and a worse prognosis[12] (Fig. 8A, B; Fig. S8A). Indeed, in the TCGA-LIHC cohort, RUVBL1 scores within the top 10 genes predicting overall survival (Fig. S8B). Gene Set Enrichment Analysis (GSEA) of RUVBL1-correlated genes in the TCGA-LIHC cohort was performed through the GENI webapp.[35] The top-scoring GSEA signatures positively correlated with RUVBL1 expression are shown in Fig. 8C, among which MYC, E2F, and mTORC1 signalling are well-known functions regulated by RUVBL1.[12,14,19,36] Indeed, the inhibitory phosphorylation of mTOR at serine 2448[37] is significantly associated with reduced RUVBL1 expression in the LIHC cohort (Fig. S8C), supporting a key metabolic role of this ATPase in human HCC. Strikingly, glycolysis and oxidative phosphorylation are also significantly enriched with RUVBL1 expression in the LIHC dataset (Fig. 8D). These data are further corroborated by the GO analysis of differentially expressed genes (DEGs), which identifies the mitochondrial compartment as a significantly enriched cellular component in HCC with high RUVBL1 expression (Fig. 8E). Finally, in human liver samples from the TCGA and GTEx databases, RUVBL1 shows a very strong correlation with (F1)-ATP synthase subunits and, to a lesser extent, with ATPIF1 and ATPAF2 (Fig. 8G).

Taken together, these findings strongly suggest that RUVBL1 expression in human HCC is associated with mitochondrial metabolic processes, including oxidative phosphorylation, and that these functions likely contribute to the increased aggressiveness of tumours with higher RUVBL1 expression.

Discussion

RuvBLs proteins are drawing increasing attention for their pleiotropic role in many key cellular processes, including DNA repair, chromatin remodelling, ribosome biogenesis, and mTOR pathway regulation. Despite a growing body of reports highlighting the connections of RuvBLs with metabolic functions,[12,14,19,20] the impact of RuvBL1 targeting on cell metabolism is still poorly understood.

Here, we present clear evidence for the role of RuvBL1 in maintaining mitochondrial integrity and metabolic functions.

We found that inhibiting RuvBL1/2 ATPase activity in Huh7 HCC cells strongly affects amino acid metabolism and ATP production from glycolysis and the TCA cycle (Figs 1 and 3; Figs. S1 and S3, Table S1). It is interesting to note that aspartate and asparagine are the only amino acids whose levels are clearly increased by CB-6644. Aspartate and asparagine largely share the same metabolic pathways at the crossroad of TCA and urea cycles. Aspartate aminotransferase (AST) catalyses the reversible conversion of glutamate to aspartate concomitantly generating alpha-ketoglutarate from oxalacetate.[38,39] Therefore, aspartate levels are key both for the malate–aspartate shuttle (which imports glycolysis-produced NADH into mitochondria) and for the anaplerotic reactions that, through amino acid catabolism in the urea cycle, replenish the TCA cycle intermediates. Indeed, the urea cycle is among the top-scoring pathways emerging from the metabolomic analysis of CB-6644-treated cells (Fig. 1C; Fig. S1B, Table S1). We previously observed that RuvBL1$^{hep+/-}$ mice have reduced levels of AST (but not ALT/GPT) compared with wild-type mice[12] and, interestingly, RUVBL1 and AST (but not ALT/GPT) are found positively correlated in human liver samples from the GTEx and TCGA cohorts (Fig. S9A, B). In amino acid-deprived cells, asparagine and aspartate serve as key exchange factors for other amino acids, in particular arginine and serine, which act as mTORC1 activators.[40] RUVBL1 and asparagine synthetase show a very strong positive correlation in human liver samples from the

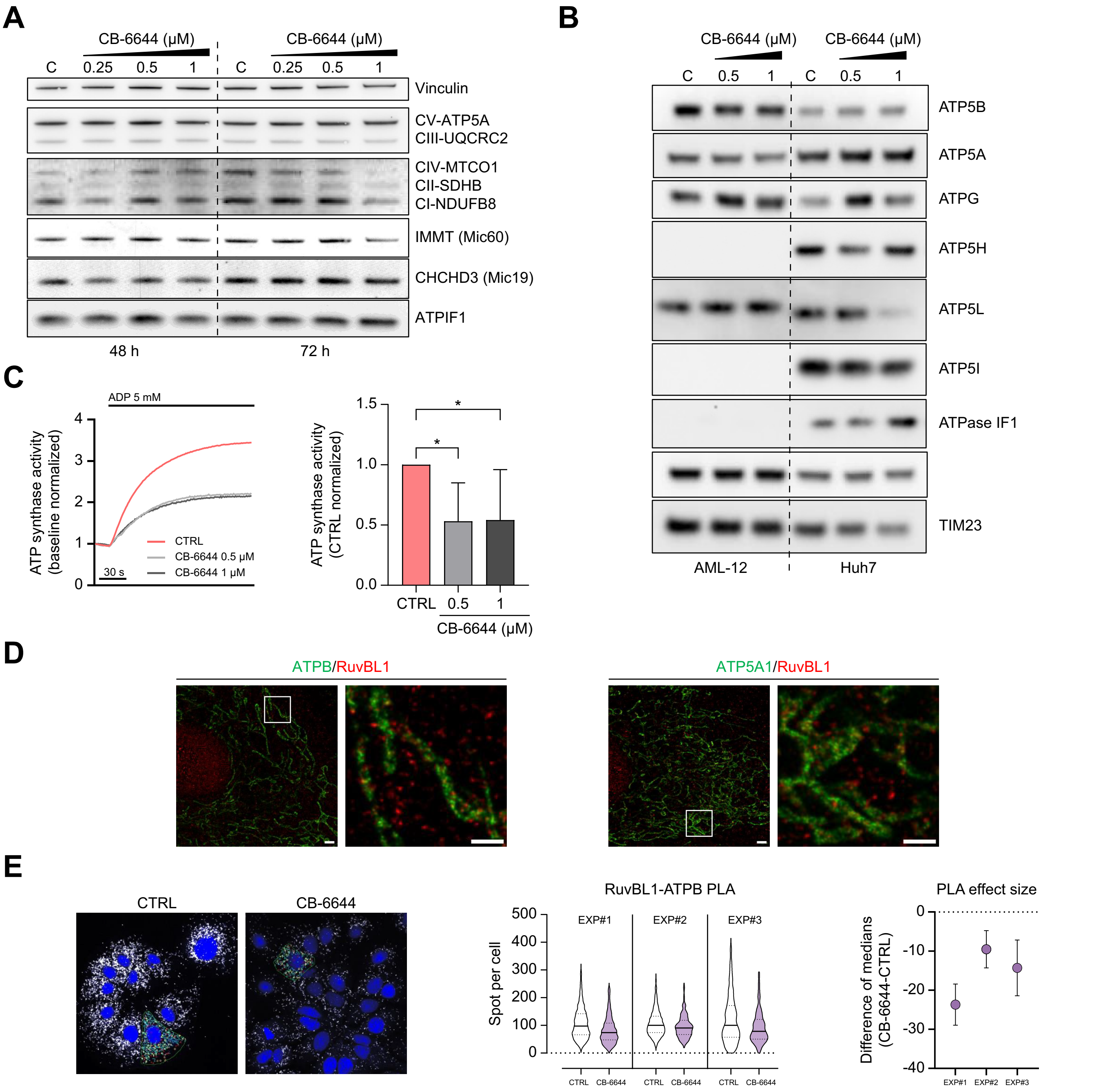

Fig. 7. CB-6644 impairs complex V activity without affecting its assembly. WB of ETC complex subunits, MICOS components, and APTIF1 in CB-6644-treated Huh7 cells. (B) WB of complex V subunits in AML-12 and Huh7 cells treated with CB-6644 for 48 h. (C) ATP synthase activity in Huh7 exposed to 0.5–1 μM CB-6644 for 72 h Huh7 cells expressing mitochondrially targeted firefly luciferase were permeabilised with digitonin, then mitochondria were energised with glutamate and malate. ATP synthesis was stimulated by the administration of 5 mM ADP. Statistical significance was calculated by one-way ANOVA with Dunnett's correction for multiple comparisons (mean ± SD, n = 3). *p <0.05. (D) STED microscopy of RuvBL1 and (F_1)-ATP synthase subunits in Huh7 cells. Scale bar = 2 μm for the larger field and = 1 μm for the enlarged detail. (E) Representative ATPB-RuvBL1 PLA images in Huh7 cells, showing cell segmentation with ScanR. The graphs show the distribution of PLA spot (count per cell) in CTRL and CB-6644-treated cell across three replicate experiments and the relative effect size with 95% CI (EXP#1: n = 1,500; EXP#2 n = 2,445; EXP#3 n = 901).

TCGA and GTEX databases (Fig. S9C), in line with the role of RuvBL1 in supporting mTORC1 activity. along this line of thought, asparagine and aspartate levels may increase in RuvBL1-targeted cells as a compensatory mechanism for reduced mTORC1 activity.

Among the amino acids whose levels decrease upon RuvBL1/2 inhibition, leucine, isoleucine, and valine are of particular interest. Under low glucose conditions, these branched-chain amino acids are degraded to produce ketone bodies, which supply the carbon backbone for TCA cycling. In

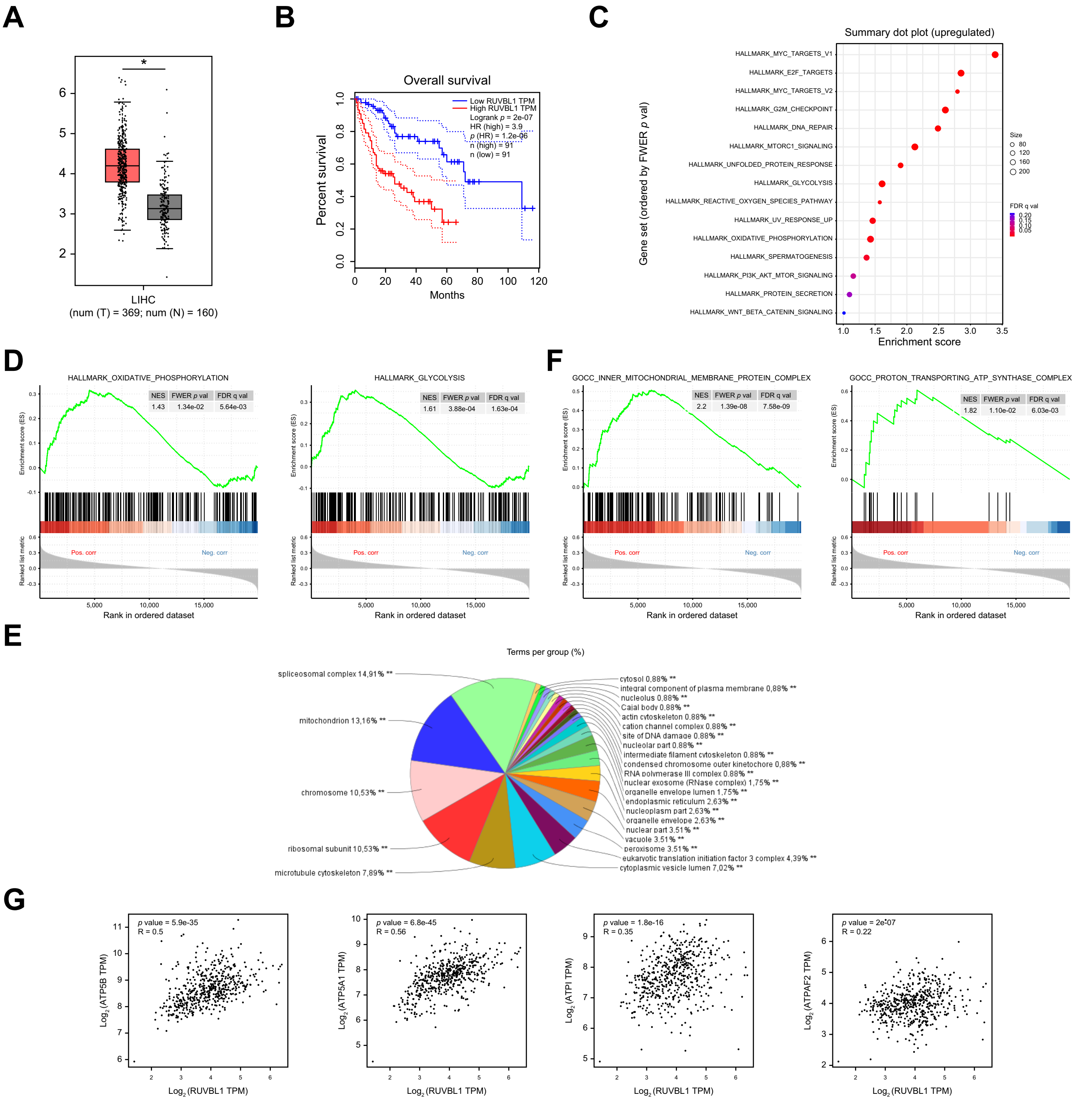

Fig. 8. RuvBL1 expression correlates with mitochondrial functions in human HCC. (A) RuvBL1 expression in HCC and non-tumoural liver samples (TCGA-LIHC and GTEX cohorts, performed with GEPIA2). (B) Overall survival of patients with HCC with high-*vs*. low-RuvBL1 expression (quartile cut-off, TCGA-LIHC cohort, performed with GEPIA2). (C) Enrichment analysis of high-RuvBL1 expressing HCC in the LIHC cohort *vs*. HALLMARK gene sets (performed with GENI). (D) GSEA profile of selected HALLMARK gene sets (performed with GENI). (E) Gene Ontology analysis of DEGs in high-*vs*. low-LIHC (cut-off z-score ± 2, performed with ClueGO). (F) GSEA of cellular components in high-RuvBL1 expressing LIHC (performed with GENI). (G) Spearman's correlation analysis of RUVBL1 and (F_1)-ATP synthase subunits, ATPIF1 and ATPAF2 in liver samples (TCGA-LIHC + GTEx, performed with GEPIA2). DEGs, differentially expressed genes; GSEA, Gene Set Enrichment Analysis; HCC, hepatocellular carcinoma; LIHC, liver hepatocellular carcinoma; TCGA, The Cancer Genome Atlas.

the context of cells maintained in complete medium, the activation of ketogenesis may appear counterintuitive. However, several clues support this interpretation; first, CB-6644 induces a significant upregulation of mitochondrial IVD and HMGHCL, two key enzymes of leucine catabolism and ketogenesis (Fig. S7B).[41–43] Second, CB-6644 increases the expression of mitochondrial D2HGDH (Fig. S7A), which catalyses the conversion of D-2-hydroxyglutarate (2-DHG) to alpha-

ketoglutarate to support the TCA cycle. 2-DHG can be produced by the 4-hydroxybutyrate catabolism,[44] oncogenic isocitrate dehydrogenase (IDH), and propionyl-CoA shunting into the TCA cycle.[45,46] Propionyl-CoA is a central intermediate metabolite of isoleucine and valine degradation[47] and can also be formed by odd-chain fatty acids, cholesterol, C-5 ketone bodies, threonine, and methionine.[48] Within the LIHC and GTEx databases, RUVBL1 expression shows a very strong negative correlation with the gene signature of propionate shunting (Fig. S9D).[49] Intriguingly, while ketone bodies produced by the liver are normally utilised by extrahepatic tissues, under nutrient deprivation stress, HCC cells have been recently shown to reactivate OXCT1 expression and ketolysis as a metabolic adaptation for energy supply.[50,51]

Taken together, these data show that the inhibition of RuvBL1/2 ATPase activity has a noticeable impact on glycolysis and on the TCA cycle, and suggests that mitochondrial metabolism is rewired towards anaplerosis, likely through amino acid degradation and ketogenesis.

Direct measurement of mitochondrial activity by Seahorse analysis confirmed that targeting RuvBL1 by RNAi (Fig. 2; Fig. S2), or CB-6644 treatment (Fig. 3; Fig. S3), reduces mitochondrial respiration and ATP production from OXPHOS. Remarkably, glycolytic ATP production was reduced by CB-6644 in all the tested cancer cell lines but not in non-transformed AML-12 cells and in primary mouse hepatocytes. Besides the altered metabolic flux described above, we collected several pieces of evidence supporting an impairment of ATP synthase activity, which may contribute to the reduced mitochondrial ATP production. In fact, CB-6644 induces an unexpected hyperpolarisation of the $\Delta\Psi m$, as shown by JC-1 and TMRM staining (Fig. 4B–D). These observations are confirmed by direct measurement of the $\Delta\Psi m$ in CB-6644-treated AML-12 cells, which is higher than in control cells and refractory to further increase by the complex V inhibitor oligomycin (Fig. 4E). Interestingly, ATPAF2, a key assembly factor for complex V,[52] was among the few proteins identified by MS as significantly upregulated by CB-6644 (Figs. S6C and S7B), which prompted us to investigate ATP synthase in more detail. Analysis of ETC complexes in whole cells and in immunoprecipitated mitochondria of Huh7 cells failed to reveal a consistent downregulation of ATP synthase subunits (Fig. 7A, B), suggesting that the assembly of complex V is not impaired. Nevertheless, CB-6644 dose-dependently increases ATPIF1, the main inhibitory factor of complex V (Fig. 7A, B),[53] and reduces ATP synthase activity even when measured in energised mitochondria (Fig. 7C).

It is conceivable that the increased expression of ATPAF2 and ATPIF1 may occur as an adaptive feedback mechanism to the impaired ATP synthase activity aimed at restoring its function and at preventing the reverse mode of action of complex V, thereby avoiding further ATP disposal, stabilising complex V dimerisation despite loss of cristae, and promoting cell survival.[53] These molecular clues are paralleled by evident alteration of the mitochondrial morphology, reduced network integrity and disarrangement of the cristae in CB-6644 treated cells (Figs 4 and 5; Fig. S5). Of notice, the genetic deletion of RuvBL1 in mature hepatocytes (RuvBL1$^{hep-/-}$ mice) recapitulates this phenotype *in vivo*, made evident by the reduced mitochondrial density and scarcity of cristae (Fig. 5B).

The altered cristae morphology is accompanied by an increase in short OPA1 isoforms (Fig. S6B) and a reduction in MICOS complex components (Fig. 7A). OPA1 and MICOS complex, together with complex V dimerisation, play a key role in the shaping of the IMM at the junctional and tip sides of the mitochondrial cristae[31,54] and are crucial for isolating the cristae lumen from the intermembrane space, thus contributing to the generation of the proton gradient driving the $\Delta\Psi m$. The unambiguous localisation of RuvBL1 within the mitochondrion (Fig. 6; Figs S6A and S7D, E), to our knowledge previously unreported, strongly suggests that this protein may directly participate in processes essential for the structural and functional integrity of this organelle. This line of thought is supported by super-resolution imaging and PLA localising RuvBL1 within 40 nm of (F1)-ATP synthase in Huh7 cells (Fig. 7D, E; Fig. S6E). The small but consistent reduction of RuvBL1-ATPB proximity caused by CB-6644 (Fig. 7E) suggests that this interaction may be required for proper complex V function and warrants further mechanistic investigation. Nevertheless, the emerging picture clearly depicts RuvBL1 as a key element supporting mitochondrial metabolism, structure, and function. In human HCC samples from the TCGA-LIHC cohort, higher RuvBL1 expression correlates with more advanced stage (Fig. S8A) and RUVBL1 scores within the 10 top genes correlating with reduced survival (Fig. 8A; Fig. S8B). RUVBL1 correlates with glycolysis and oxidative phosphorylation in GSEA (Fig. 8C, D), which agrees with its role in supporting mTORC1 signalling (Fig. 8C; Fig. S8C).[37] Consistent with our metabolomic results, RUVBL1 also negatively correlates with branched-chain amino acids degradation and propanoate metabolism (Fig. S8D). Interestingly, a prominent proportion of genes differentially expressed between LIHC samples with high-*vs* low-RuvBL1 expression are annotated with the term ‘mitochondrion’ in GO cellular component analysis (Fig. 8E). Indeed, the IMM and the ATP synthase complex are among the cellular components terms significantly enriched for RUVBL1 in the LIHC cohort (Fig. 8F), and RUVBL1 gene expression shows a strong positive correlation with (F_1)-ATP synthase components and regulators in human liver samples from the TCGA and GTEx database (Fig. 8G). It is therefore tempting to speculate that, among the several potential tumour-promoting functions of RuvBL1,[3] supporting the mitochondrial metabolic processes and complex V activity are key to human HCC. As RuvBL1 overexpression and mitochondrial metabolic rewiring are shared features across several cancer types, future studies will need to assess the relevance of mitochondrial RuvBL1 in context beyond HCC.

In conclusion, we uncovered a novel metabolic function and cellular localisation of the AAA+ ATPase RuvBL1, which interacts with ATP synthase and emerges as a central regulator of mitochondrial activity. As RuvBL1 plays multiple roles in human pathophysiology, including cancer, further research is warranted to exploit its mitochondrial localisation and related functions as potential therapeutic targets.

Affiliations

[1]*Department of Clinical and Experimental Biomedical Sciences "Mario Serio", University of Florence, Florence, Italy;* [2]*Department of Medical Sciences, Section of Experimental Medicine, and Laboratory for Technologies of Advanced Therapies (LTTA), University of Ferrara, Ferrara, Italy;* [3]*Department of Experimental and Clinical Medicine, Imaging Platform, University of Florence, Florence, Italy;* [4]*Department of Philosophy, Social Sciences, Humanities and Education, University of Perugia, Perugia, Italy;* [5]*Maria Cecilia Hospital, GVM Care & Research, Cotignola, Italy*

Abbreviations

2-DHG, D-2-hydroxyglutarate; AST, aspartate aminotransferase; ATPAF2, ATP synthase mitochondrial F1 complex Assembly Factor 2; DEGs, differentially expressed genes; ETC, electron transport chain; GC-MS, gas chromatography-mass spectroscopy; GO, Gene Ontology; GSEA, Gene Set Enrichment Analysis; HCC, hepatocellular carcinoma; IB, intracellular buffer; IBluc, IB containing 25 μM luciferin; IDH, isocitrate dehydrogenase; IMM, inner mitochondrial membrane; LIHC, liver hepatocellular carcinoma; OXPHOS, oxidative phosphorylation; PLA, proximity ligation assay; TCGA, The Cancer Genome Atlas; TMRM, tetramethyl rhodamine methyl ester, OPA1, Optic Atrophy 1, OCR, oxygen comsumption rate.

Financial support

This work was supported by the Italian Association for Cancer Research (AIRC) with grants IG-20590 (to AG) and IG-23670 (to PP). The study was also supported by Progetti di Rilevante Interesse Nazionale (PRIN20227Z2XRB to MB; 2020RRJP5L, 202259LHXM, P2022WY85 K_001, and PNRR-CN00000041 to PP), by the Italian Ministry of Health - Tuscany Region with grant GR-1600315 awarded to TM, and by the Multi-User Equipment Program by AIRC and Fondazione Cassa di Risparmio di Firenze for the Molecular Medicine Facility of the Department of Clinical and Experimental Biomedical Sciences "Mario Serio", University of Florence.

Conflicts of interest

The authors declare no conflicts of interest.
Please refer to the accompanying ICMJE disclosure forms for further details.

Authors' contributions

Conceptualization: TM. Project administration: TM, AG. Investigation: TM, IS, AG, DP, FB, AS, DG, PN, SP, EC, AC, MB. Formal analysis: TM, IS, AG, DP, FB, AS, DG, SP, MB. Resources: ML, OB, PP, MB. Methodology: PP, MB. Validation: PP, MB. Supervision: AG. Funding acquisition: AG. Writing – original draft, review & editing: TM. Writing – review & editing: AG, DP, FB, PP, MB, AG.

Data availability

The data that support the findings of this study are available from the corresponding author upon reasonable request. The mass spectrometry proteomics data have been deposited to the ProteomeXchange Consortium via the PRIDE[55] partner repository with the dataset identifier PXD075574 and 10.6019/PXD075574.

Acknowledgements

We are grateful to Prof. Daniele Bani (Department of Clinical and Experimental Medicine, University of Florence) for insightful discussion and guidance on TEM analysis, to Prof. Lorenzo Di Cesare Mannelli (Department of Neuroscience, Psychology, Drug Research and Child Health – NEUROFARBA, University of Florence) for providing access to the High Content Imaging platform, and to Dr. Giulia Cantini (Department of Clinical and Experimental Biomedical Sciences "Mario Serio", University of Florence, Florence) for editing the graphical abstract.

Supplementary data

Supplementary data to this article can be found online at https://doi.org/10.1016/j.jhepr.2026.101858.

References

Author names in bold designate shared co-first authorship

[1] **Li W**, **Zeng J**, **Li Q**, et al. Reptin is required for the transcription of telomerase reverse transcriptase and over-expressed in gastric cancer. Mol-Cancer 2010;9:132.

[2] Huber O, Menard L, Haurie V, et al. Pontin and reptin, two related ATPases with multiple roles in cancer. Cancer Res 2008;68:6873–6876.

[3] Grigoletto A, Lestienne P, Rosenbaum J. The multifaceted proteins reptin and pontin as major players in cancer. Biochim Biophys Acta 2011;1815:147–157.

[4] Matias PM, Baek SH, Bandeiras TM, et al. The AAA+ proteins pontin and reptin enter adult age: from understanding their basic biology to the identification of selective inhibitors. Front Mol Biosci 2015;2:17.

[5] Breig O, Bras S, Martinez SN, et al. Pontin is a critical regulator for AML1-ETO-induced leukemia. Leukemia 2014;28:1271–1279.

[6] Lauscher JC, Elezkurtaj S, Dullat S, et al. Increased pontin expression is a potential predictor for outcome in sporadic colorectal carcinoma. Oncol Rep 2012;28:1619–1624.

[7] Ishii T, Akiyama Y, Shimada S, et al. Identification of a novel target of SETD1A histone methyltransferase and the clinical significance in pancreatic cancer. Cancer Sci 2023;114:463–476.

[8] Mikesch J-H, Hartmann W, Angenendt L, et al. AAA+ ATPases reptin and pontin as potential diagnostic and prognostic biomarkers in salivary gland cancer - a short report. Cell Oncol (Dordr) 2018;41:455–462.

[9] Durślewicz J, Wybierała AM, Szczepanek S, et al. RUVBL1 in clear-cell renal cell carcinoma: unraveling prognostic significance and correlation with HIF1A. Cancers (Basel) 2024;16:1273.

[10] Tian J, Wen M, Gao P, et al. RUVBL1 ubiquitination by DTL promotes RUVBL1/2-β-catenin-mediated transcriptional regulation of NHEJ pathway and enhances radiation resistance in breast cancer. Cell Death Dis 2024;15:259.

[11] Haurie V, Ménard L, Nicou A, et al. Adenosine triphosphatase pontin is overexpressed in hepatocellular carcinoma and coregulated with reptin through a new posttranslational mechanism. Hepatology 2009;50:1871–1883.

[12] Mello T, Materozzi M, Zanieri F, et al. Liver haploinsufficiency of RuvBL1 causes hepatic insulin resistance and enhances hepatocellular carcinoma progression. Int J Cancer 2020;146:3410–3422.

[13] Mannar D, Ahmed S, Subramaniam S. AAA ATPase protein–protein interactions as therapeutic targets in cancer. Curr Opin Cell Biol 2024;86:102291.

[14] Vogt M, Dudvarski Stankovic N, Cruz Garcia Y, et al. Targeting MYC effector functions in pancreatic cancer by inhibiting the ATPase RUVBL1/2. Gut 2024;73:1509–1528.

[15] Chen D, Ji F, Zhou Q, et al. RUVBL1/2 blockade targets YTHDF1 activity to suppress m6A-dependent oncogenic translation and colorectal tumorigenesis. Cancer Res 2024;84:2856–2872.

[16] Assimon VA, Tang Y, Vargas JD, et al. CB-6644 is a selective inhibitor of the RUVBL1/2 complex with anticancer activity. ACS Chem Biol 2019;14:236–244.

[17] Izumi N, Yamashita A, Iwamatsu A, et al. AAA+ proteins RUVBL1 and RUVBL2 coordinate PIKK activity and function in nonsense-mediated mRNA decay. SciSignal 2010;3:ra27.

[18] López-Perrote A, Serna M, Llorca O. Maturation and assembly of mTOR complexes by the HSP90-R2TP-TTT chaperone system: molecular insights and mechanisms. Subcell Biochem 2024;104:459–483.

[19] Kim SG, Hoffman GR, Poulogiannis G, et al. Metabolic stress controls mTORC1 lysosomal localization and dimerization by regulating the TTT-RUVBL1/2 complex. Mol Cell 2013;49:172–185.

[20] **Shin SH**, **Lee JS**, **Zhang J-M**, et al. Synthetic lethality by targeting the RUVBL1/2-TTT complex in mTORC1-hyperactive cancer cells. Sci Adv 2020;6:eaay9131.

[21] Kakihara Y, Makhnevych T, Zhao L, et al. Nutritional status modulates box C/D snoRNP biogenesis by regulated subcellular relocalization of the R2TP complex. Genome Biol 2014;15:404.

[22] **Chen T**, **Yuan Z**, **Lei Z**, et al. Hippocalcin-Like 1 blunts liver lipid metabolism to suppress tumorigenesis via directly targeting RUVBL1-mTOR signaling. Theranostics 2022;12:7450–7464.

[23] **Gao B**, **Lu Y**, **Lai X**, et al. Metabolic reprogramming in hepatocellular carcinoma: mechanisms of immune evasion and therapeutic implications. Front Immunol 2025;16:1592837.

[24] Wang Q, Liu J, Chen Z, et al. Targeting metabolic reprogramming in hepatocellular carcinoma to overcome therapeutic resistance: a comprehensive review. Biomed Pharmacother 2024;170:116021.
[25] Ziki RA, Colnot S. Glutamine metabolism, a double agent combating or fuelling hepatocellular carcinoma. JHEP Rep 2024;6:101077.
[26] Franzè MS, Saffioti F, Mavroeidis VK. Interactions between tumor microenvironment and resistance to transarterial and systemic treatments for HCC. Cancer Drug Resist 2025;8:33.
[27] Bereshchenko O, Mancini E, Luciani L, et al. Pontin is essential for murine hematopoietic stem cell survival. Haematologica 2012;97:1291–1294.
[28] Herwig R, Hardt C, Lienhard M, et al. Analyzing and interpreting genome data at the network level with ConsensusPathDB. Nat Protoc 2016;11:1889–1907.
[29] Gottschalk B, Koshenov Z, Malli R, et al. Implications of mitochondrial membrane potential gradients on signaling and ATP production analyzed by correlative multi-parameter microscopy. Sci Rep 2024;14:14784.
[30] Kondadi AK, Anand R, Reichert AS. Cristae membrane dynamics – a paradigm change. Trends Cell Biol 2020;30:923–936.
[31] **Stephan T**, **Brüser C**, **Deckers M**, et al. MICOS assembly controls mitochondrial inner membrane remodeling and crista junction redistribution to mediate cristae formation. EMBO J 2020;39:e104105.
[32] Ježek P, Jabůrek M, Holendová B, et al. Mitochondrial cristae morphology reflecting metabolism, superoxide formation, redox homeostasis, and pathology. Antioxid Redox Signal 2023;39:635–683.
[33] Fogo GM, Raghunayakula S, Emaus KJ, et al. Mitochondrial membrane potential and oxidative stress interact to regulate Oma1-dependent processing of Opa1 and mitochondrial dynamics. FASEB J 2024;38:e70066.
[34] Chen WW, Freinkman E, Sabatini DM. Rapid immunopurification of mitochondria for metabolite profiling and absolute quantification of matrix metabolites. Nat Protoc 2017;12:2215–2231.
[35] Hayashi A, Ruppo S, Heilbrun EE, et al. GENI: a web server to identify gene set enrichments in tumor samples. Comput Struct Biotechnol J 2023;21:5531–5537.
[36] **Tarangelo A**, **Lo N**, **Teng R**, et al. Recruitment of pontin/reptin by E2f1 amplifies E2f transcriptional response during cancer progression. Nat-Commun 2015;6:10028.
[37] Figueiredo VC, Markworth JF, Cameron-Smith D. Considerations on mTOR regulation at serine 2448: implications for muscle metabolism studies. Cell Mol Life Sci 2017;74:2537–2545.
[38] Ling Z-N, Jiang Y-F, Ru J-N, et al. Amino acid metabolism in health and disease. Sig Transduct Target Ther 2023;8:1–32.
[39] Chandel NS. Amino acid metabolism. Cold Spring Harb Perspect Biol 2021;13:a040584.
[40] Krall AS, Xu S, Graeber TG, et al. Asparagine promotes cancer cell proliferation through use as an amino acid exchange factor. Nat Commun 2016;7:11457.
[41] **Arnedo M**, **Latorre-Pellicer A**, Lucia-Campos C, et al. More than one HMG-CoA lyase: the classical mitochondrial enzyme plus the peroxisomal and the cytosolic ones. Int J Mol Sci 2019;20:6124.
[42] Adeva-Andany MM, López-Maside L, Donapetry-García C, et al. Enzymes involved in branched-chain amino acid metabolism in humans. Amino Acids 2017;49:1005–1028.
[43] Puchalska P, Crawford PA. Multi-dimensional roles of ketone bodies in fuel metabolism, signaling, and therapeutics. Cell Metab 2017;25:262–284.
[44] Struys EA, Verhoeven NM, Brunengraber H, et al. Investigations by mass isotopomer analysis of the formation of D-2-hydroxyglutarate by cultured lymphoblasts from two patients with D-2-hydroxyglutaric aciduria. FEBS Lett 2004;557:115–120.
[45] Kaufman EE, Nelson T, Fales HM, et al. Isolation and characterization of a hydroxyacid-oxoacid transhydrogenase from rat kidney mitochondria. J Biol Chem 1988;263:16872–16879.
[46] **Xiao D**, **Zhang W**, Guo X, et al. A d-2-hydroxyglutarate biosensor based on specific transcriptional regulator DhdR. Nat Commun 2021;12:7108.
[47] Crown SB, Marze N, Antoniewicz MR. Catabolism of branched chain amino acids contributes significantly to synthesis of odd-chain and even-chain fatty acids in 3T3-L1 adipocytes. PLoS One 2015;10:e0145850.
[48] Halarnkar PP, Blomquist GJ. Comparative aspects of propionate metabolism. Compar Biochem Physiol B 1989;92:227–231.
[49] Watson E, Olin-Sandoval V, Hoy MJ, et al. Metabolic network rewiring of propionate flux compensates vitamin B12 deficiency in *C. elegans*. eLife 2016;5:e17670.
[50] **Guo D**, **Yu Q**, **Tong Y**, et al. OXCT1 succinylation and activation by SUCLA2 promotes ketolysis and liver tumor growth. Mol Cell 2025;85:843. 56.e6.
[51] **Huang D**, **Li T**, Wang L, et al. Hepatocellular carcinoma redirects to ketolysis for progression under nutrition deprivation stress. Cell Res 2016;26:1112–1130.
[52] Wang Z-G, White PS, Ackerman SH. Atp11p and Atp12p are assembly factors for the F1-ATPase in human mitochondria. J Biol Chem 2001;276:30773–30778.
[53] Gatto C, Grandi M, Solaini G, et al. The F1Fo-ATPase inhibitor protein IF1 in pathophysiology. Front Physiol 2022;13:917203.
[54] Anand R, Reichert AS, Kondadi AK. Emerging roles of the MICOS complex in cristae dynamics and biogenesis. Biology 2021;10:600.
[55] Perez-Riverol Y, Bandla C, Kundu DJ, et al. The PRIDE database at 20 years: 2025 update. Nucleic Acids Res 2025;53:D543–D553.

Keywords: OXPHOS; ATP synthase; TCA cycle; Amino acids metabolism; Ketogenesis; Liver cancer; Metabolic reprogramming.
Received 5 September 2025; received in revised form 31 March 2026; accepted 7 April 2026; Available online 17 April 2026

Journal of Hepatology, Volume *8*

Supplemental information

Targeting RuvBL1 disrupts mitochondrial metabolism and structure in hepatocellular carcinoma

Tommaso Mello, Irene Simeone, Alice Guida, Dimitri Papini, Francesca Begnozzi, Alice Santi, Daniele Guasti, Patrizia Nardini, Simone Polvani, Matteo Lulli, Oxana Bereshchenko, Elisabetta Ceni, Armando Curto, Paolo Pinton, Massimo Bonora, and Andrea Galli

Fig. S8

Targeting RuvBL1 disrupts mitochondrial metabolism and structure in hepatocellular carcinoma

Tommaso Mello, Irene Simeone, Alice Guida, Dimitri Papini, Francesca Begnozzi, Alice Santi, Daniele Guasti, Patrizia Nardini, Simone Polvani, Matteo Lulli, Oxana Bereshchenko, Elisabetta Ceni, Armando Curto, Paolo Pinton, Massimo Bonora, Andrea Galli

Table of contents

Materials and methods……………………………………………………………………….2

Supplementary figure legends……………………………………………………..……14

Supplementary figures………………………………………………………..........…..……18

Table S1……………………………………………………………………………………...27

Supplementary statistics……………………………………………………separate excel file

Supplementary references…………………………………………………………...….29

Materials and methods

Primary hepatocytes isolation

All procedures involving laboratory animals were conducted in accordance with institutional ethical norms and national laws, following approval from the Italian Ministry of Health (D.No. 30/2013 and D.No. 665/2018). Hepatocytes were isolated from 3 months old C57/BL6 mice by collagenase-dispase perfusion through the portal vein. Livers were perfused in situ with 45 ml of Gibco Liver Perfusion Media (Invitrogen. Carlsbad, CA) followed by 45 ml of Gibco Liver Digestion Media (ThermoFisher Scientific, Italy). The liver was excised, minced, and strained through 100μM and 70μM EASYstrainers (Greiner BIO-ONE). The dispersed hepatocytes were collected by centrifugation at 50g for 2 minutes at 4°C. Hepatocytes were separated by gradient centrifugation in 40% Percoll (Sigma-Aldrich) at 200g for 10 minutes at 4°C and washed twice with pre-cooled plating media (MEM containing 5% FBS and supplemented with GlutaMAX (Gibco), penicillin and streptomycin (Sigma Aldrich). The hepatocyte pellet was then resuspended with plating media, counted and viability was assessed by Trypan Blue exclusion. Typical yield was around $6*10^6$ hepatocytes per liver with >95% viability. Primary hepatocytes were plated in collagen coated plates (Sigma-Aldrich C8919, 10ug/cm^2). After 2.5h, the medium was changed to HepatoZYME-SFM (Gibco) supplemented with Pen/Strep, GlutaMAX and collagen I (1.25ug/cm^2) for sandwich culture.

Cell culture

Authenticated cell lines were obtained from suppliers reported in the supplementary CTAT table. Upon arrival, cell lines were amplified for 3 to 4 passages, tested for mycoplasma (Sigma-Aldrich), aliquoted and stored in liquid nitrogen. Thawed cells were passed twice to ensure full recovery and used within passage 20. All cell lines were cultured in media supplemented with 10% FBS, stable glutamine (GlutaMAX, Gibco) and without antibiotics. Mycoplasma was tested regularly by PCR. Hepa1-6 and Huh7 were maintained in DMEM, HepG2 and Hep3B in MEM, AML-12 in DMEM/F12 supplemented with Selenium, Transferrin, Insulin, Hepes and Dexamethasone.

Silencing was performed using siRNA (iBONI siRNA, Riboxx GmbH) to a final concentration of 20nM in all cell lines. Sequence details are reported in the Supplementary CTAT Table.

Generation of cells with epitope-tagged mitochondria

pMXs-3XHA-EGFP-OMP25 and pMXs-3XMyc-EGFP-OMP25 constructs were purified using the PureYield™ Plasmid Miniprep kit. 1.5 µg of DNA was transfected into Huh7 cells using FuGENE HD at a 4.5:1 lipid:DNA ratio. 48 hours post-transfection, cells were selected with 150 µg/mL Blasticidin and then FACS-sorted for EGFP signal. pMXs-3XHA-EGFP-OMP25 (Addgene plasmid #83355) and pMXs-3XMyc-EGFP-OMP25 (Addgene plasmid #83356) were a gift from David Sabatini[1].

Gene expression analysis

RNA was extracted from Huh7, Hep3B and HepG2 cell lines using the RNeasy mini kit (Qiagen) and complementary DNA was synthesized by the PrimeScript RT Reagent Kit (Promega) according to the manufacturer's protocol. qPCR was performed using 20ng of retrotranscribed RNA per reaction and qPCR validated primers (Qiagen). Gene expression was quantified by qPCR using the ΔΔCt method and β-2 microglobulin as a reference gene. Master mix was Luna Universal qPCR Master Mix (New England Biolabs), thermal cycler was Rotor- Gene Q (Qiagen), and analysis was run on Q-Rex Software (Qiagen). P values were calculated from the ΔCt distributions using Student's t-test.

Seahorse Analysis

The day before transfection, cells were plated in 24 well plates to reach 40-50% confluence in 24h (AML-12, Hepa1-6, Huh-7 and Hep3B: 25.000cells/well; HepG2: 40.000cells/well). Gene silencing was performed with 20nM IBONI siRNA (Riboxx GmBH) or 5nM Silencer Select validated siRNA (Thermo Fisher), using negative-control siRNA and GAPDH siRNA to evaluate silencing efficiency and transfection efficiency, respectively. INTERFERin (Polyplus) or RNAiMAX (Invitrogen) were used as transfection reagents. After 48h, silenced cells were trypsinized and plated in the Seahorse XFe 96 well plate (HepG2, Huh7: 10.000 cells/well; Hep3B, AML-12, Hepa1-6: 8000cells/well). The

following day (72h after siRNA transfection) the Seahorse MitoStress test was performed following the manufacturer protocol. Immediately after the assay, cells were fixed with 4% PFA, then stained with 1uM TO-PRO-3 Iodide (Life technologies) for 30' to label the nuclei and imaged with a Typhoon Scanner equipped with a 633nm laser line a Cy5 filter set (670nm, bandpass) (GE Healthcare). The integrated density of TO-PRO-3 in each well was quantified with Fiji/ImageJ[2] and used to normalize OCR values.

For OCR evaluation after CB-6644 treatment, cells were seeded directly in the Seahorse XFe 96well plate (HepG2, Hep3B, Huh-7: 5000cells/well; AML-12, Hepa1-6: 3000cells/well). 6 hours after seeding, cells were treated with CB-6644 diluted in 20ul of complete culture media for 24h to 72h. MitoStress test (Agilent) and Real Time ATP Rate Assay (Agilent) analysis were performed following the manufacturer protocol, and OCR values were normalized by TO-PRO-3 iodide signal intensity as described above.

Mouse primary hepatocytes were plated in Seahorse XFe 96-well plates, 4000cells/well in 80uL/well of Hepatocyte Plating Medium (Gibco) pre-coated with 10ug/cm^2 of collagen (Sigma-Aldrich). After 2.5h, the plating medium was carefully removed, and primary hepatocytes were sandwiched with collagen (1,25ug/cm^2) diluted in 80ul/well of HepatoZYME-Serum Free Medium (Gibco) containing Pen/Strep (Sigma-Aldrich) and Glutamax (Gibco). The following day the cells were treated with CB-6644 diluted in 20uL/well of HepatoZYME-SFM containing antibiotics and glutamine.

Metabolomic analysis

Huh7 cells were plated in 6-well plates (300.000cells/well) in 3 ml of complete medium (DMEM high glucose, 10% FBS, glutamine) and treated with 0.5 µM of CB6644 or vehicle alone. After 48h, each well was washed twice with cold saline solution (0.9% NaCl) and cells were scraped in 400 µl of 80% cold methanol supplemented with 1 µg/ml of norvaline (Merck, 53721), used as an internal standard. The cell suspension was sonicated 3 times for 5 seconds on ice using a pulse sonicator (Bandelin Sonopuls HD2070, 40% pulse time, 50% power output) and then centrifuged for 15 min at 14.000 x g. The supernatant was stored at -80°C until processed, while the pellet was dissolved with

100 µl of 200 mM NaOH for 15 min at 95°C and used to determine protein concentration by the BCA method. The supernatant was dried using a vacuum concentrator (Labconco). Dried extracts were derivatized in 10 µl of 40 mg/mL methoxamine hydrochloride (Merck, 226904) in pyridine (Merck, 270970) at 37 ◦C for 90 min, followed by 50 µl of MTBSTFA (Merck, 375934) at 60 °C for 30 min. Data acquisition was performed by using an Intuvo 9000 GC/5977B MS System (Agilent Technologies) equipped with an HP-5MS capillary column (30 m × 0.25 mm x 0.25 µm). 1 µL of each sample was injected in splitless mode using an inlet liner temperature of 240 °C. GC runs were performed with helium as carrier gas at 1 mL/min. The GC oven temperature ramp was from 70 °C to 280 °C. The temperature of 70 °C was held for 2min. Then, the first temperature ramp was from 70 °C to 140 °C at 3 °C/min. The second ramp was from 140 °C to 150 °C at 1 °C/min. The third temperature ramp was from 150 °C to 280 °C at 3 °C/min. Metabolite measurements were performed under electron impact ionization at 70 eV using SIM mode. The ion source and transfer line temperatures were set to 230 °C and 290 °C, respectively. For data analysis, the MS Quantitative Analysis software (version 10.2 Agilent) and an in-house library were used. For relative metabolite abundances, the peak area of each metabolite was normalized to norvaline and to protein concentration.

Statistical analysis was performed on triplicate experiments using Graphpad Prism 10 or Metaboanalyst (MetaboAn:https://www.metaboanalyst.ca/MetaboAnalyst/home.xhtmlalyst). Statistical significance was assessed using an unpaired t-test with unequal variances and FDR correction. Metabolite enrichment analysis, hierarchical clustering and PCA were performed in Metaboanalyst after autoscaling. Over-representation analysis (ORA) was performed using the Consensus Path Database[3] web tool (http://cpdb.molgen.mpg.de/) with the list of significantly modulated metabolites identified by Metaboanalyst as input.

Super-resolution STED microscopy

Cells were grown on highly corrected coverslips (170um ± 5um, Menzel GmbH) and fixed with 4% paraformaldehyde (EM grade, EMS cat.15710) for 20 minutes at room temperature.

Cells were washed three times with PBS and then permeabilized with PBS containing 0.1% Triton X-100 for 15 minutes at room temperature. After three washes with PBS, cells were incubated in blocking buffer (5% normal goat serum in PBS) for 30 minutes at room temperature. Primary antibodies (anti-Pontin 1:50, Sigma Aldrich; anti-TOMM20 1:500 Abcam; anti-ATPB dilution Proteintech, anti-ATP5A1 dilution Proteintech) were incubated in blocking buffer overnight at 4°C and secondary antibodies (Invitrogen's goat anti-mouse AlexaFluor568, goat anti-mouse Alexa Fluor 532, goat anti-rabbit AlexaFluor532 and goat-anti rabbit AlexaFluor555 plus, all diluted 1:100) were incubated for 1 hour at room temperature. Coverslips were mounted in Prolong Glass antifade medium (ThermoFisher Scientific). STimulated Emission Depletion (STED) images were collected through an HCPLAPO100X 1.4NA oil objective with a Leica TCS SP8 STED microscope equipped with a supercontinuum white light laser and a 660nm STED laser and deconvolved using Huygen Professional Software.

Electron microscopy

Samples were fixed with 2% formaldehyde and 2.5% glutaraldehyde in 0.1M cacodylate buffer (pH 7.4) and then embedded in epoxy resin. Ultra-thin sections (~70 nm thick) were mounted on gold grids and immunostained overnight at 4° with a monoclonal mouse primary antibody (anti-pontin, SAB4200194) diluted in filtered 1% BSA (1:100). After washes with filtered PBS-BSA 1% solution, a goat anti-mouse secondary antibody conjugated with 10 nm colloidal gold particles was diluted in filtered 1% BSA (1:20) and incubated in a humid chamber for 2 hours at 37°. Samples were rinsed in two steps, first with filtered PBS-BSA 1% solution and then with pure water. After counterstaining with UranyLess (Electron Microscopy Sciences) and alkaline bismuth subnitrate, samples were observed using a JEM 1010 electron microscope (Jeol, Tokyo, Japan) at 80 kV. Photomicrographs were captured with a digital camera, MegaView III (Soft Imaging System, Muenster, Germany), connected to a computer (Dell, Round Rock, Texas) with dedicated software (AnalySIS, Soft Imaging System, Muenster, Germany).

Single Molecule Localization Microscopy

Cells were fixed in 4% PFA for 10 min, washed in PBS, permeabilized with 0.1% Triton X-100 for 10 min, and incubated in 6 M urea (pH 9.0) for 5 min at 80 °C to improve epitope–antibody binding. Therefore, nonspecific binding sites were blocked by incubation in 0.1% Triton X-100 supplemented with 2% BSA for 45 min at room temperature. Cells were then incubated overnight at 4°C with primary antibodies against RuvBL1 (Rabbit, antibody dilution 1:200, 10210-2-AP, Proteintech) and ATP5A (Mouse, antibody dilution 1:100, ab14748, Abcam). Primary antibodies were detected using CF660 (Donkey Rabbit IgG (H+L) 20816, Biotium) - and CF680 (Donkey Mouse IgG (H+L) 20817, Biotium) -conjugated secondary antibodies. dSTORM imaging was performed in an imaging buffer that included Buffer A (10 mM Tris-HCl pH 8.0 + 50 mM NaCl + 10% Glucose), 0,56 mg/mL Glucose Oxidase (Cohesion Biosciences), 0,34 mg/mL Catalase (Serva) and 50 mM cysteamine (Sigma-Aldrich).

Single-molecule imaging was performed using a SAFe MN360 microscope (Abbelight), equipped with two ORCA-Fusion digital cameras (Hamamatsu) and controlled by Abbelight NEO acquisition software. 2D dual-color SMLM images were acquired using an UPlanApo 100×/NA 1.5 TIRF oil-immersion objective (Olympus) with HiLo illumination. Excitation was performed using 640 nm laser. Dual-color imaging was achieved by spectral demixing using a dual-camera detection scheme. For each acquisition, 45000 frames were recorded with an exposure time of 44 ms.

Image analysis was performed using Abbelight NEO analysis software. Single-molecule localization was carried out prior to image reconstruction. In the reconstructed super-resolution images, each detected molecule was represented as a Gaussian spot centered at its centroid position, with localization precision determined from the single-molecule fitting procedure.

Colocalization analysis was performed using Coloc-Tesseler [DOI: 10.1038/s41467-019-10007-4], which computes Voronoi diagrams from localization data. Colocalization was quantified using the Spearman rank correlation coefficient .

Proximity Ligation Assay (PLA)

For PLA, Huh7 cells were seeded on glass coverslips and incubated for 24h in DMEM/10%FBS. After 24h cells were treated with 1µM of CB-6644 for 72h and then fixed with 4% of paraformaldehyde (20min at room temperature). Cells were permeabilized (0.1% Triton X-100) and incubated with the Blocking buffer included in the PLA kit Naveni™TriFlex Cell. Incubation with primary antibody (overnight at 4°C) and PLA assay were performed following the manufacturer's protocol. Cell nuclei were labelled with DAPI. Images were taken with the high content screening system ScanR (Evident Scientific) with a 40X/0.95 UPlanXApo (Evident Scientific). Quantification of the PLA signal was obtained with the ScanR software.

Western blotting

Proteins were extracted using RIPA assay buffer supplemented with protease and phosphatase inhibitors. Extracts were sonicated and quantified using the BCA assay. Protein samples were resolved on NuPAGE 4-12% Bis-Tris precast SDS-polyacrylamide gels and transferred to polyvinylidene difluoride membranes. Membranes were blocked with 5% skim milk and incubated overnight with primary antibodies in 5% bovine serum albumin (BSA). Subsequently, membranes were incubated for 1 hour at room temperature with horseradish peroxidase-conjugated anti-rabbit IgG (LICOR), diluted 1:50.000 in BSA solution. Protein detection was performed using Enhanced Chemiluminescence Select (Cytiva) and visualized with the ImageQuant350 system (GE Healhcare).

Mitochondrial immunoprecipitation

Huh7 cells endogenously tagged with $OMP25^{HA}$ and $OMP25^{MYC}$ were seeded in 15 cm plates. All buffers were supplemented with protease inhibitors. At 80% confluency, the cells were washed twice with cold PBS containing protease inhibitors (Sigma), then once with cold KPBS buffer (136 mM KCl, 10 mM KH_2PO_4, 50 mM sucrose, pH 7.2) supplemented with protease inhibitors. The cells were harvested on ice by scraping and pelleted at 1000g for 5 min at 4°C. The cell pellet was resuspended in 1 mL KPBS with protease inhibitors and lysed using 30 strokes in a 2 mL homogenizer. The lysate

was spun down at 1000g for 5 min at 4°C. The pellet was discarded, and the input sample was incubated with 50 µL of anti-HA magnetic beads (Pierce). The beads were washed with KPBS three times before incubation with samples. The mixture was placed on gentle rotation for 20 min at 4°C. After incubation, the beads were washed twice with KPBS containing 300 mM NaCl and once with KPBS buffer. The samples were then eluted with 100 µL of KPBS containing 0.5% NP-40 in a thermomixer at 30°C for 20 min. Eluates for mass spectrometry were snap-frozen in liquid nitrogen and stored at -80°C until further processing. The same experiment was repeated two times to obtain a biological triplicate.

Proteomics analysis

Samples were first reduced using dithiothreitol (DTT) and alkylated with iodoacetamide at room temperature. Protein precipitation was achieved by adding 100 % methanol. Precipitates were pelleted for 2 min at 4 °C. The pellets were dried and resuspended in 6 M urea. Peptide digestion was carried out with trypsin (Promega) overnight at 37°C. Tryptic peptides were desalted and dried in a vacuum centrifuge prior to mass spectrometry analysis. The samples were then resuspended in water and 0.1% trifluoracetic acid (TFA) and analysed by mass spectrometry. LC-MS/MS analyses were performed on a Q-Exactive HF-X Orbitrap mass spectrometer (Thermo Fisher Scientific). Peptide separation was carried out using a PepMap RSLC C18 column (75 um × 15 cm, 2 µm, 100 Å, Thermo Fisher) at a flow rate of 300 nl/min. The mobile phases A and B used for the analysis were 0.1% formic acid in water and 0.1% formic acid in acetonitrile, respectively. The gradient started with 5% B and increased to 90% over 120 min. The experiment was performed using a data dependent analysis (DDA) setting to select the "top twenty" most-abundant ions for MS/MS analysis. Proteome Discoverer 2.5 (Thermo Scientific) performed protein identification. The peptide spectra were matched against *Homo sapiens* database downloaded from Uniprot (TaxId: 9606). The analysis was based on at least one unique peptide with a minimum length of seven amino acids and a false discovery rate (FDR) of 0.01. The default peak-picking settings were used to process the raw MS files in MaxQuant [4] (version 1.6.1.0) and its integrated search engine Andromeda [5]. Protein

relative quantification and calculation of statistical significance were carried out using a two-tailed Student's t-test with error correction (p-value < 0.05) and the Benjamini–Hochberg method. Moreover, a volcano plot, summarizing the distribution of differentially expressed proteins was generated with Perseus software (version 1.6.1.1) [6]. Data are available via ProteomeXchange with identifier PXD075574.

Cell fractionation and mitochondria purification

Aml-12, Huh7, Hep3B and HepG2 cells were seeded in 15 cm cell plates. For every cell line, 30 plates were used for mitochondria isolation. Cells were collected and immediately transferred on ice. Samples were washed twice with cold PBS and subsequently homogenized in isolation buffer (250 mM sucrose, 10 mM HEPES, pH 7.4, 1 mM EDTA) supplemented with a protease inhibitor cocktail. The samples were homogenized, and crude mitochondria were isolated following the protocol from Wieckowski et al.[7]. The protein concentration of the isolated fraction was determined using the BCA assay. The fractions were aliquoted and stored at -80°C for subsequent analysis.

Complex V activity

Complex V activity was assessed by a bioluminescent luciferin-luciferase assay [8]. Huh7 cells were seeded onto 13 mm glass coverslips and transiently transfected with a mitochondrially targeted luciferase chimera (MT Luc). After 48 hours of treatment with CB-6644 (0.5 µM or 1 µM), coverslips were mounted in a thermostated perfusion chamber, and real-time luminescence was recorded with a custom-built luminometer. Recordings began with a 30-second baseline in intracellular buffer (IB; 130 mM KCl, 10 mM NaCl, 0.5 mM KH_2PO_4, 1 mM $MgSO_4$, 5 mM sodium succinate, and 20 mM HEPES, pH 7.4), designed to mimic the cytosolic ionic composition. Cells were then perfused with IB containing 25 µM luciferin (IBluc). Within 120-180 seconds, luciferase catalysed light production, reaching a plateau as it reacted with intracellular ATP. Plasma membrane permeabilization was achieved by perfusing cells with 25 µM digitonin (Sigma-Aldrich) in IBluc. After permeabilization, cells were sequentially exposed to IBluc supplemented with 1 mM malic acid and 1 mM glutamic

acid (Sigma-Aldrich), followed by 5 mM ADP (Sigma-Aldrich). The resulting increase in luminescence, measured in counts per second (cps), reflected ATP synthesis driven by Complex V activity in response to exogenous ADP.

Mitochondrial mass and membrane potential analysis

Cells were plated in 96wells in the appropriate culture media and incubated with Mitotracker Deep Red FM (final concentration 200nM) and Calcein AM (final concentration 2uM) in FluorBrite DMEM for 20 minutes at 37°C. After labelling, the plate was imaged with a dual channel Typhoon confocal scanner (GE Healthcare) at a resolution of 25um/line. The integrated intensity of the Mitotracker signal in each well was normalized to the Calcein AM signal and measured with Fiji software[2]. JC-1 labelling (1uM in FluorBrite DMEM, 30' at 37°C) was performed on cells plated on optically clear 4-sectors 35mm dishes (Greiner Bio-One, cat. 627975) to ensure consistent labelling and imaging of control and CB-6644 treated cells within the same experimental session. Images were acquired with a Leica AM6000 microscope equipped with a stage incubator (Pecon), a Leica DFC350FXR2 camera and an HCX PL Fluotar 20x0.4NA objective. The ratio of red to green JC-1 signal was measured with Fiji software.

Cells were incubated for 30 min at 37 °C in a solution of modified Krebs-Ringer buffer (mKRB: 135 mM NaCl, 5 mM KCl, 0.4 mM KH_2PO_4, 1 mM $MgSO_4$, 20 mM HEPES, 5.5 mM glucose and 1 mM $CaCl_2$ (pH 7.4)) containing 2 nM tetramethyl rhodamine methyl ester (TMRM; Life Technologies, T-668), Verapamil hydrochloride 20µM (Merck KGaA, V4629), Hoechst 33342 1.6 µM (Thermo Fisher, H3570) and SYTOX™ Green Nucleic Acid Stain 170 nM (Thermo Fisher, S7020). Acquisitions were maintained at 37 °C and captured using a UPLXAPO 20X/0.8 air objective on a Olympus IX83 inverted microscope. Excitation was performed at 561 nm and emission was collected at 590-650 nm. Images were analysed using the scanR High-Content Screening Station (Life sciences, Evident). Briefly, Hoechst 33342 signal was used to define the area of cell nuclei and a mask was generated. The intensity of the SYTOX™ Green was measured within the nuclei of all cells

identified in the first step, and all cells positive for the signal were marked as dead. Then, TMRM average fluorescent units (AFU) were quantified in SYTOX™ Green-negative cells, and each condition was compared with the untreated control.

Mitochondrial network morphometry

Huh7 and AML12 cells were grown on coated glass coverslips and then fixed with paraformaldehyde 4% for 10 minutes at room temperature. The cells were permeabilized with PBS/0.05% Triton X-100 for 10 minutes and blocked for 1 hr in PBS/0.05% Triton X-100 containing 3% BSA before incubation with theTOMM20 primary antibody. Secondary antibodies were Alexa Fluor 594-conjugated. DAPI was used to counterstain DNA. Slides were mounted with ProLong Gold Antifade Mountant (Life Technologies). Z-stack acquisitions (51 planes, one each 0,3 μM) were captured from at least six different fields using a confocal laser scanning microscopy (Olympus FV3000) equipped with a 60X oil immersion objective (PLAPON60XOSC2, N.A.1.4). Pixel size was set at 80 nm. Alexa Fluor 594 was excited at 561 nm, and emission was collected at 590-650 nm.

The Z-stacks were deconvolved using Huygens Essential software (Scientific Volume Imaging B.V.) and a theoretical PSF. Following image reconstruction, single cells were isolated from each acquisition and processed in ImageJ (Fiji software) to calculate the number of objects (count), total (sum), mitochondrial density (total mitochondrial volume/cell volume), and per cell average values of: object volume, object sphericity, object compactness, object surface area, object elongation, and object flatness. The threshold for each cell was automatically calculated using the Ridler-Calvard algorithm. Morphological indexes were computed using the 3D suite plugin [9] All data were then grouped in Excel the imported into an R project to calculate principal component analysis. The first two PCA components were then used to cluster using K-means algorithm. The number of clusters was set to 3, corresponding to 3 classes of mitochondrial network: fragmented, intermediate and connected. The percentage of each mitochondrial network morphology class per condition was then calculated.

In silico analysis

RUVBL1 expression in normal liver (TCGA and GTEx) and HCC samples of the TCGA_LIHC cohort was evaluated through the GEPIA2 web tool [10], last accessed on the 18th of January 2026. Overall Survival analysis and most differential survival gene analysis in the LIHC cohort were performed in GEPIA2 using RUVBL1 expression quartiles (75%-25%) as cut-off values for group definition. RUVBL1 expression level in HCC stages was also graphed within GEPIA2.

Gene Set Enrichment Analysis (GSEA) of the LIHC cohort (Firehose Legacy, 373 samples) was performed using the web app GENI [11] with Spearman correlation and default settings (last accessed on the18th of January 2026). The TCGA database was accessed through the cBioPortal for cancer genomics[12] to retrieve RuvBL1 mRNA expression data in the Liver Hepatocellular Carcinoma cohort (LIHC). Patients were assigned to the HI_RUVBL1 or LOW_RUVBL1 groups based on a Z-score of >2. Twenty-nine (8%) of 371 fully sequenced patients were assigned to the HI_RUVBL1 group. The mRNA expression data of genes significantly enriched in the two groups were used to run a Gene Ontology analysis with ClueGo (Cytoscape app).

Statistics

Statistics analysis was performed with GraphPad Prism 10, from three or more replicates. Achieved statistical significance levels and type of test used are reported in figure legends with standard notations: * $p<0.05$, ** $p<0.01$, *** $p<0.001$, **** $p<0.0001$. Exact p-values for each analysis are reported in the supplementary materials.

Supplementary figure legends

Fig. S1. Metabolomics analysis.

A) Principal Components Analysis of metabolomics data in CTRL and CB-6644 treated Huh7 cells. **B)** Enrichment analysis of metabolic pathways in CB-6644 treated Huh7 cells. Both analyses were performed with MetaboAnalyst web tools.

Fig. S2. RuvBL1 knockdown impairs mitochondrial respiration.

A) Line graph: Seahorse MitoStress Test profiles of RuvBL1-silenced human cell lines. OCR values are normalized by cell number and scaled relative to the basal OCR of non-silenced cells (mean ± SEM). Bar-graph: Quantification of the basal respiratory capacity shown in panel **A (**mean ± SD). Statistical significance was calculated by one-way ANOVA with Dunnett's correction for multiple comparisons. * $p<0.05$, ** $p<0.01$, *** $p<0.001$, **** $p<0.0001$. **B)**. RuvBL1 mRNA expression in control and RuvBL1-silenced cells. Representative qPCR analysis of RuvBL1 inhibition in the three cell lines. Statistical significance (vs Neg Ctrl siN1, mean ± SD) was calculated by 1-way ANOVA with Dunnett's correction for multiple comparisons. * $p<0.05$, ** $p<0.01$, *** $p<0.001$, **** $p<0.0001$.

Fig. S3. Inhibition of RuvBL1/2 ATPase activity impairs OXPHOS and ATP production.

A) Line graph: Seahorse MitoStress profile of cell lines and primary mouse hepatocytes treated with CB-6644 for 24h (mean ± SEM, n=3 to 5 independent experiments). OCR values are normalized by cell number and scaled relative to the basal OCR of non-treated cells. Bar-graph: Quantification of the basal respiratory capacity shown in panel **A (**mean ± SD). Statistical significance was calculated by one-way ANOVA with Dunnett's correction for multiple comparisons. * $p<0.05$, ** $p<0.01$, *** $p<0.001$, **** $p<0.0001$. **B)** ATP-rate assay showing the relative contribution of glycolysis and OXPHOS to the total ATP production in cells treated with CB-6644 for 24h. Statistical significance

(vs CTRL, mean ± SEM) was calculated by 2-way ANOVA with Dunnett's correction for multiple comparisons. * p<0.05, ** p<0.01, *** p<0.001, **** p<0.0001.

C) Line graph: Seahorse MitoStress profile of cell lines and primary mouse hepatocytes treated with CB-6644 for 48h (mean ± SEM, n=3 to 5 independent experiments). OCR values are normalized by cell number and scaled relative to the basal OCR of non-treated cells. Bar-graph: Quantification of the basal respiratory capacity shown in panel **A (**mean ± SD). Statistical significance was calculated by one-way ANOVA with Dunnett's correction for multiple comparisons. * p<0.05, ** p<0.01, *** p<0.001, **** p<0.0001. **D)** ATP-rate assay showing the relative contribution of glycolysis and OXPHOS to the total ATP production in cells treated with CB-6644 for 48h. Statistical significance (vs CTRL, mean ± SEM) was calculated by 2-way ANOVA with Dunnett's correction for multiple comparisons. * p<0.05, ** p<0.01, *** p<0.001, **** p<0.0001.

Fig. S4. Inhibition of RuvBL1/2 ATPase activity increases mitochondrial polarization.

A) Relative Mitotracker intensity measured in cell lines and primary mouse hepatocytes exposed to the indicated doses of CB-6644 for 72h. Statistical significance was calculated by one-way ANOVA with Dunnett's correction for multiple comparisons (median, min to max, n=3). * p<0.05, ** p<0.01.

B) Quantification of oxidized to reduced JC-1 ratio in cells exposed to CB-6644 for 72h. Statistical significance was calculated by one-way ANOVA with Dunnett's correction for multiple comparisons (median, min to max, n=3). * p<0.05, ** p<0.01, *** p<0.001, ****. p<0.0001.

C, D) Analysis of steady state mitochondrial membrane potential through high content confocal imaging of the potentiometric dye TMRM in AML-12 (**C**) and Huh7 (**D**). Statistical significance was calculated by one-way ANOVA with Dunnett's correction for multiple comparisons (median, min to max, n=3). * p<0.05, ** p<0.01**E)** Analysis of mitochondrial hyperpolarization in AML-12 cells exposed to CB-6644 (0.5 μM, 72 hours) or vehicle. Mitochondrial membrane potential was assessed by confocal imaging using the potentiometric dye TMRM. Cells were then challenged with ATP synthase inhibition via oligomycin (1 μM). Hyperpolarization was quantified as the change in TMRM

fluorescence intensity after 30 minutes of oligomycin treatment, relative to baseline values (median, min to max, n=3). Statistical significance was calculated by non-parametric Mann- Whitney test. * $p<0.05$.

Fig. S5. CB-6644 alters mitochondrial morphology.

TEM images depicting the morphology of mitochondria in CTRL and CB-6644-treated cell lines (0.25 uM for 48h). Loss of mitochondrial matrix electron density, cristae swelling and disruption are visible in CB-6644 treated cells. Original magnifications: 50k for Huh7, Hep3B, Hepa1-6, 80k for AML-12 and HepG2.

Fig. S6. CB-6644 promotes OPA-1 cleavage and ATPAF2 expression.

A) WB analysis of RuvBL1 in mitochondrial/cytosol fractionation of human cell lines. TOMM20 and Vinculin were used as positive control of mitochondria fraction and cytosol fraction respectively. **B)** WB of OPA-1 fragments in Huh7 cells treated for 48h with CB-6644 or for 24h with oligomycin 2.5 uM. **C)** WB analysis of ATPAF2 in immunoprecipitated mitochondria of CTRL and CB-6644 (0.5uM, 48h) -treated Huh7. **D)** Representative kinetic of light emitted by mitochondrial-targeted firefly luciferase expressed in Huh7 during the determination of ATP synthase activity. Huh7 cells were exposed to intracellular buffer supplemented with luciferin 25 μM (IBluc), then permeabilized with digitonin 20 μM. After successful permeabilization of the plasma membrane (represented by drop in light emission) mitochondria were energized by supplementation with malate 1 mM and glutamate 1mM. ATP synthesis was then stimulated by the administration of 5 mM ADP. **E)** STORM (Single Molecule Localization Microscopy) microscopy of RuvBL1 (red) and ATP5A (green) in Huh7 cell lines. Representative colocalization map and images of analysed cells. Scale bar = 2um for the larger field and =1um for the enlarged detail. Correlation analysis calculated by Spearman coefficient (mean ± SD).

Fig. S7. Mitoproteome analysis of CB-6644 treated Huh7 cells.

A) Mitochondrial proteins selectively detected in immunoprecipitated mitochondria of CTRL or CB-6644-treated cells (1 uM for 48h). **B)** Volcano plot and table of mitochondrial proteins differentially expressed in CTRL or CB-6644 treated cells.

Fig. S8. RuvBL1 expression correlates with disease progression and metabolism in human HCC.

A) RUVBL1 positively correlates with stage in the TCGA_LIHC cohort. Analysis performed with GEPIA2 **B)** RUVBL1 scores within the 10 top enriched genes affecting OS in the TCGA_LIHC cohort. Analysis performed with GEPIA2 **C)** Phosphorylation of mTOR S2448 is significantly correlated with RUVBL1 expression in the TGCA_LIHC cohort (RUVBL1 cut-off value Z±0.67, performed with cBioportal). **D)** GSEA analysis showing inverse correlation between RUVBL1 expression and ketogenic pathways in TCGA_LIHC cohort (performed with GENI).

Fig. S9. RuvBL1 correlation in human normal liver and HCC samples.

The analyses were performed through the GEPIA2 web portal. **A)** RUVBL1-AST correlation, **B)** RUVBL1-ALT correlation, **C)** RUVBL1-ASNS correlation, **D)** Correlation of RUVBL1 with conserved genes of involved in the propionate shunt (ACADSB, ECHS1, HIBCH, ADHFE1, ALDH6A1)[13].

Supplementary figures

Fig. S1

A

B

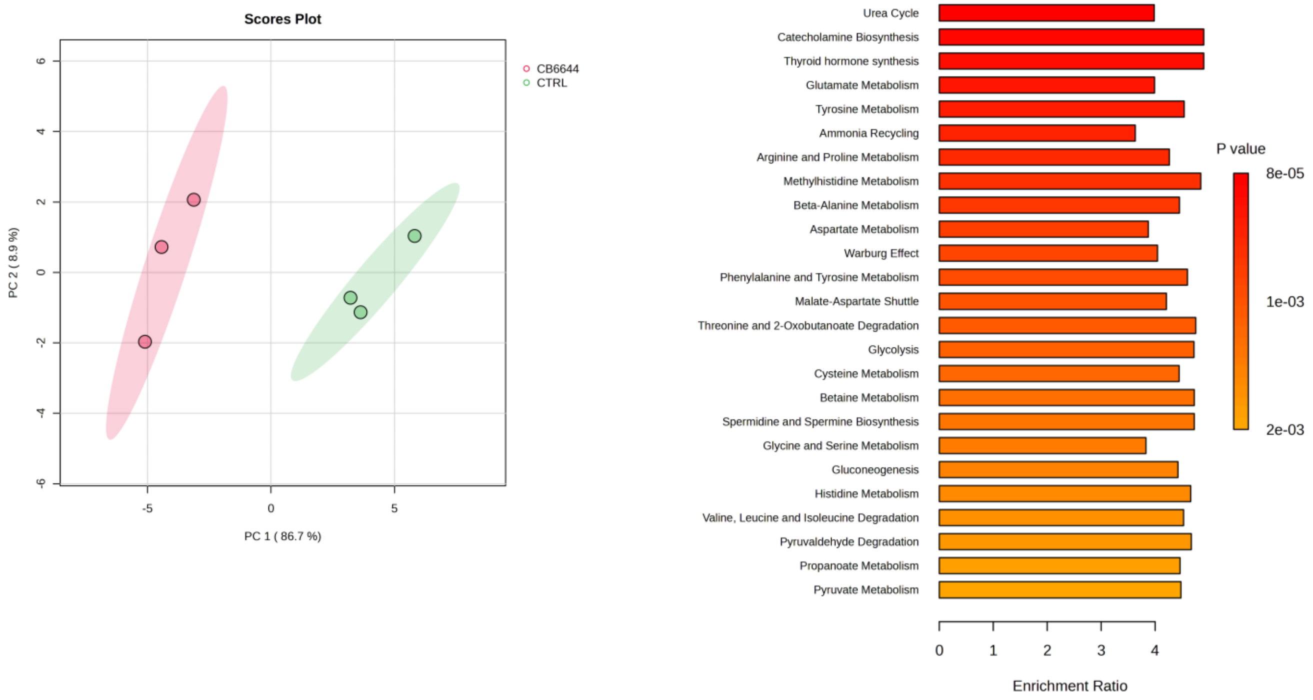

Fig. S2

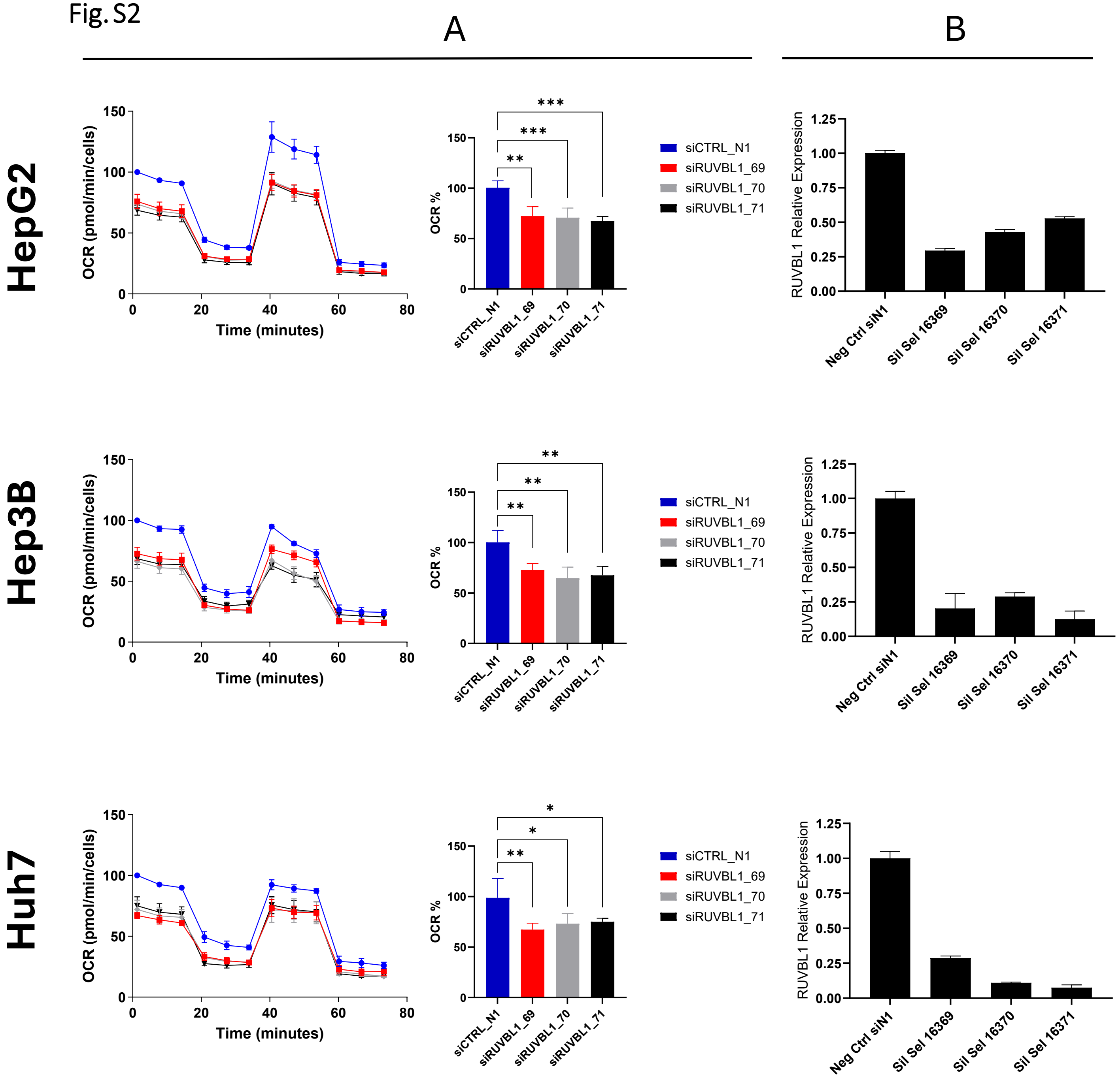

A
B
HepG2
Hep3B
Huh7
OCR (pmol/min/cells)
Time (minutes)
OCR %
siCTRL_N1
siRUVBL1_69
siRUVBL1_70
siRUVBL1_71
RUVBL1 Relative Expression
Neg Ctrl siN1
Sil Sel 16369
Sil Sel 16370
Sil Sel 16371

Fig. S3

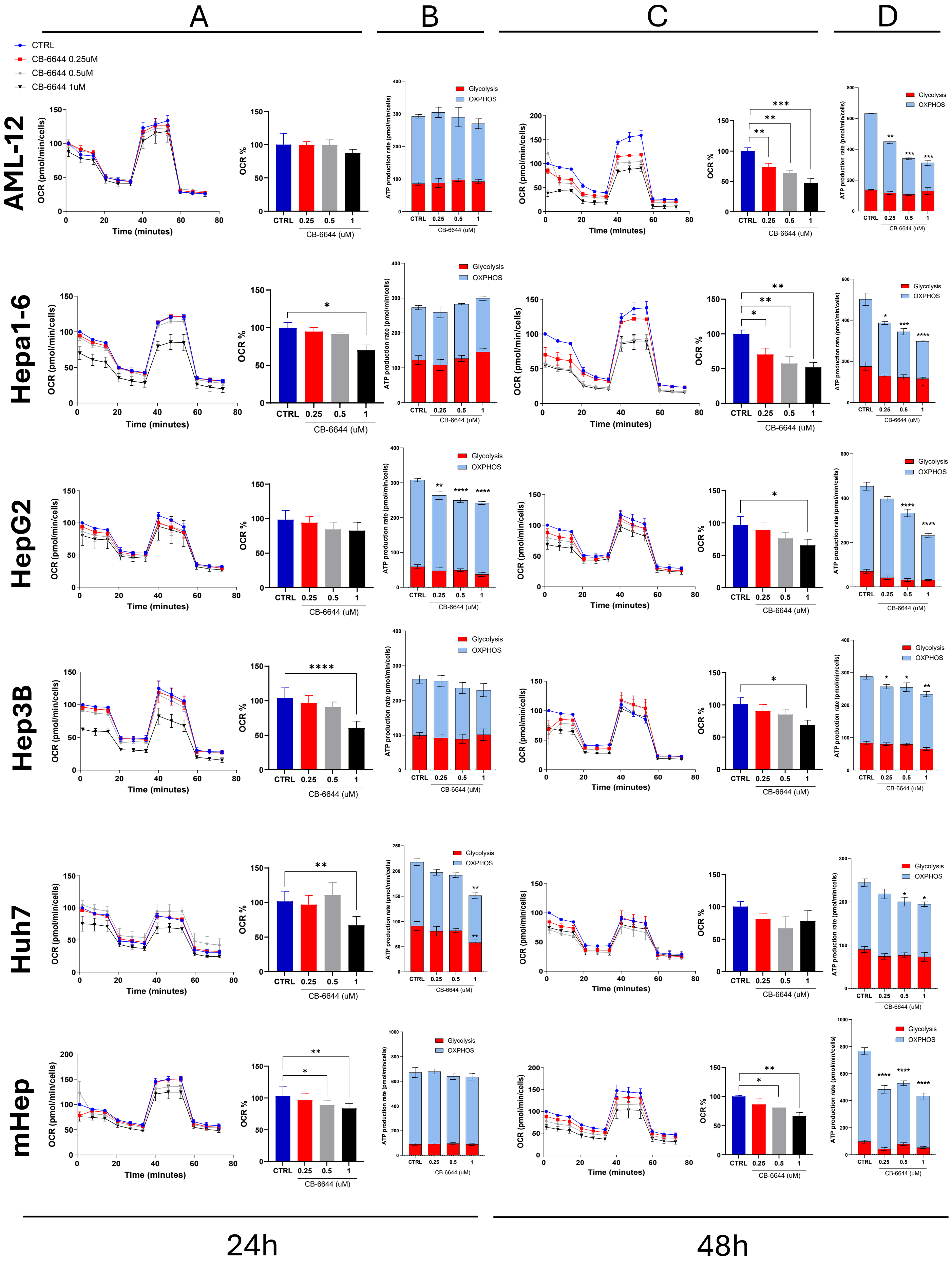

A
B
C
D
CTRL
CB-6644 0.25uM
CB-6644 0.5uM
CB-6644 1uM
AML-12
Hepa1-6
HepG2
Hep3B
Huh7
mHep
OCR (pmol/min/cells)
Time (minutes)
OCR %
CB-6644 (uM)
ATP production rate (pmol/min/cells)
Glycolysis
OXPHOS
24h
48h

Fig. S4

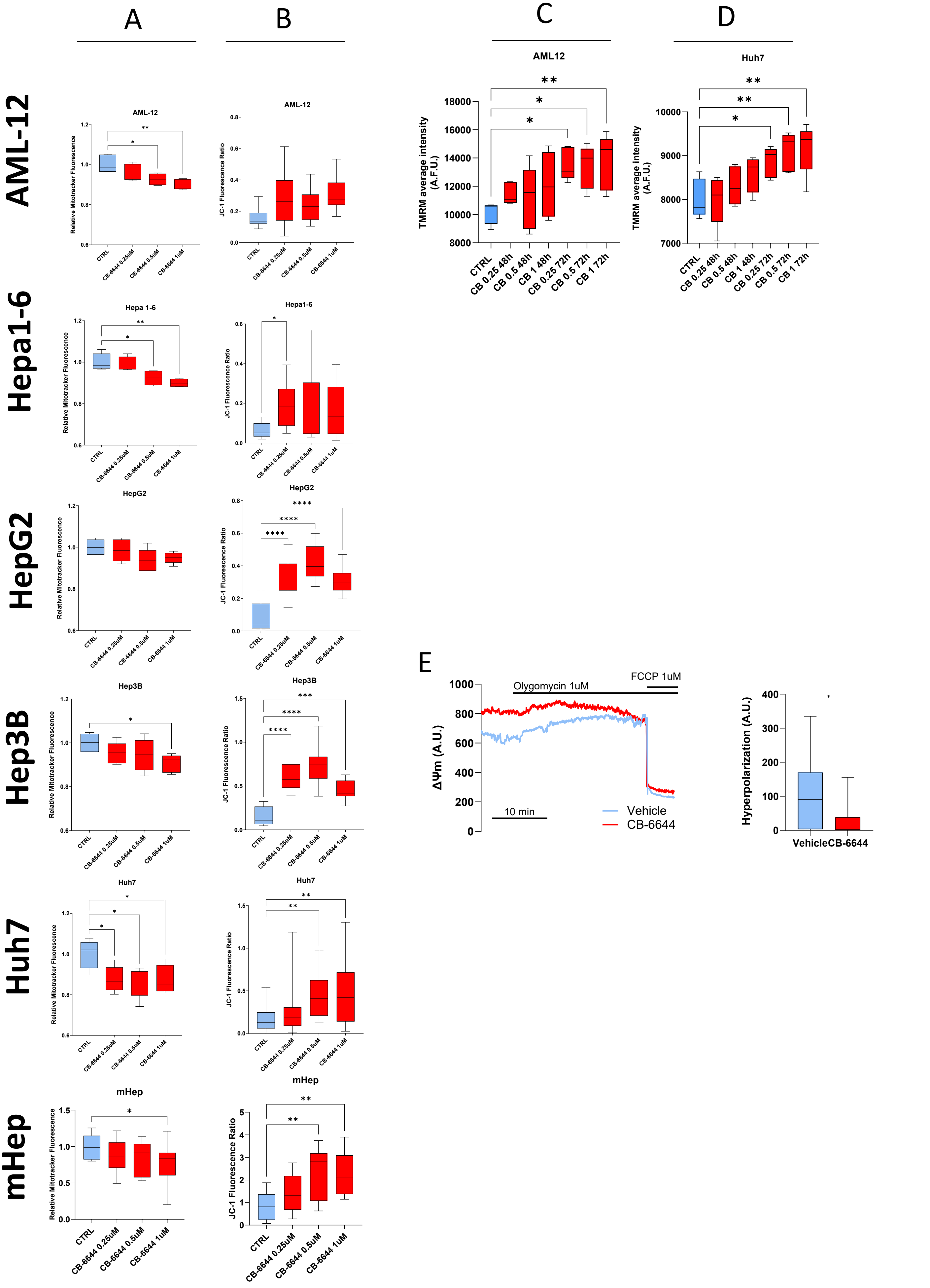

A
B
C
D
E
AML-12
Hepa1-6
HepG2
Hep3B
Huh7
mHep
AML-12
Hepa 1-6
Hepa1-6
HepG2
Hep3B
Huh7
mHep
AML12
Relative Mitotracker Fluorescence
JC-1 Fluorescence Ratio
TMRM average intensity (A.F.U.)
CTRL
CB-6644 0.25uM
CB-6644 0.5uM
CB-6644 1uM
CB 0.25 48h
CB 0.5 48h
CB 1 48h
CB 0.25 72h
CB 0.5 72h
CB 1 72h
Olygomycin 1uM
FCCP 1uM
ΔΨm (A.U.)
10 min
Vehicle
CB-6644
Hyperpolarization (A.U.)
VehicleCB-6644

Fig. S5

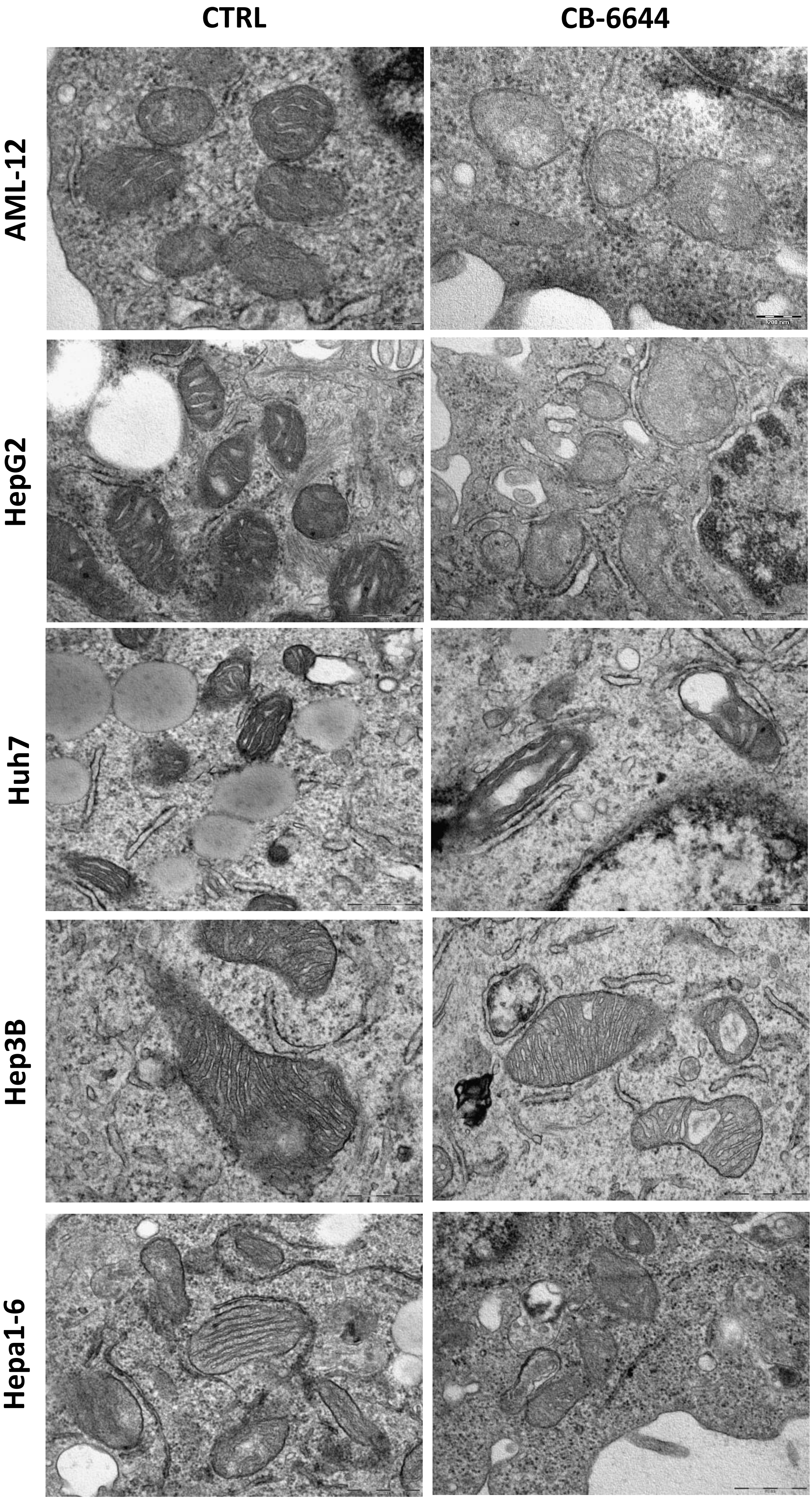

Fig. S6

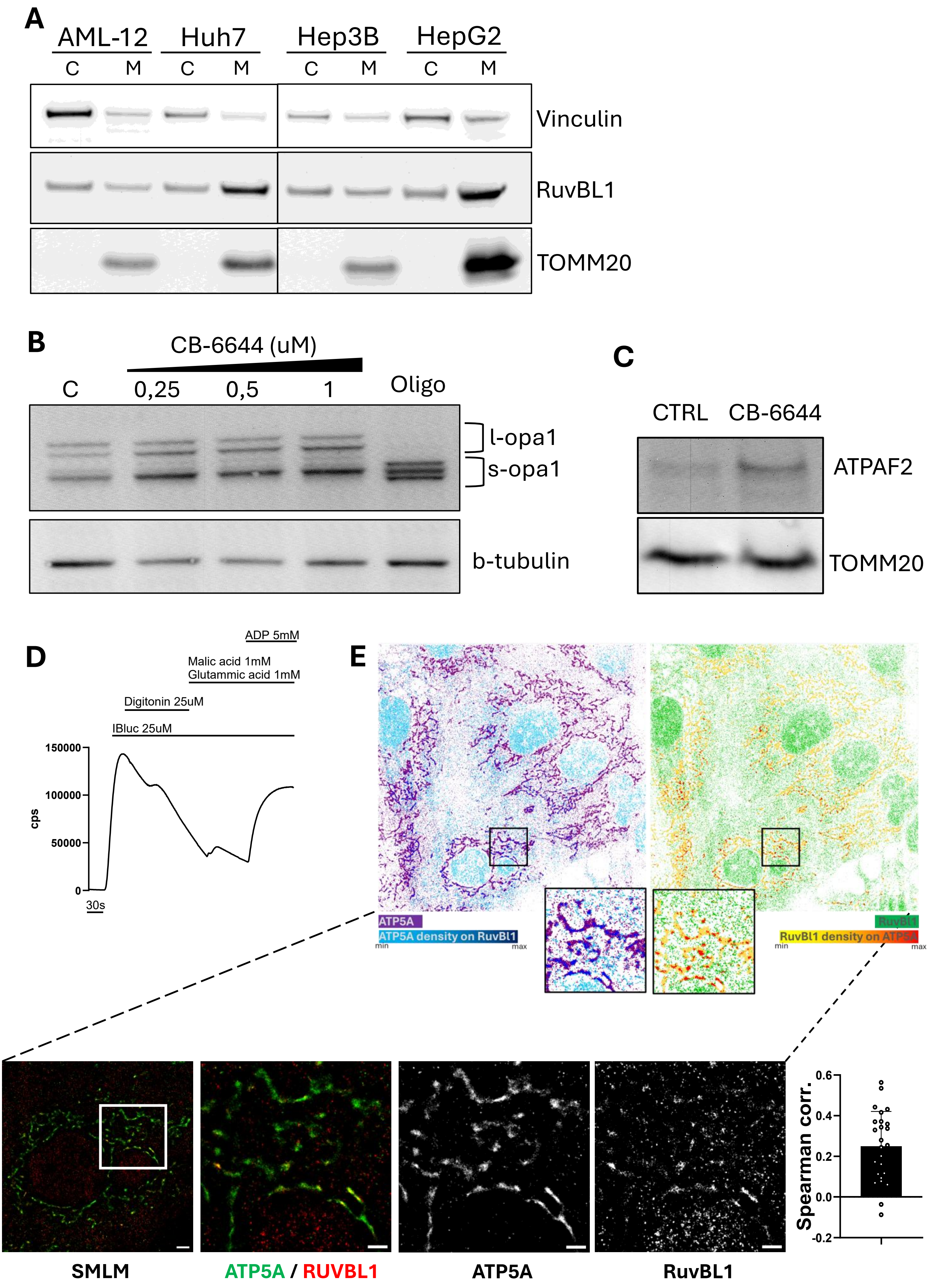

A
AML-12
Huh7
Hep3B
HepG2
C
M
C
M
C
M
C
M
Vinculin
RuvBL1
TOMM20
B
CB-6644 (uM)
C
0,25
0,5
1
Oligo
l-opa1
s-opa1
b-tubulin
C
CTRL
CB-6644
ATPAF2
TOMM20
D
ADP 5mM
Malic acid 1mM
Glutammic acid 1mM
Digitonin 25uM
IBluc 25uM
150000
100000
50000
0
cps
30s
E
ATP5A
ATP5A density on RuvBl1
min
max
RuvBl1
RuvBl1 density on ATP5A
min
max
Spearman corr.
0.6
0.4
0.2
0.0
-0.2
SMLM
ATP5A / RUVBL1
ATP5A
RuvBL1

Fig. S7

A

FDR confidence	Accession	Description	Gene Symbol	Group
High	P08133	Annexin A6	ANXA6	CTRL only
High	P01116	GTPase KRas	KRAS	CTRL only
High	Q86TS9	39S ribosomal protein L52, mitochondrial	MRPL52	CTRL only
High	Q9P2J9	Pyruvate dehydrogenase phosphatase 2, mitochondrial	PDP2	CTRL only
High	P36873	Serine/threonine-protein phosphatase PP1-gamma catalytic subunit	PPP1CC	CTRL only
High	O00743	Serine/threonine-protein phosphatase 6 catalytic subunit	PPP6C	CTRL only
High	Q8N357	Solute carrier family 35 member F6	SLC35F6	CTRL only
High	Q96QK1	Vacuolar protein sorting-associated protein 35	VPS35	CTRL only
High	Q13686	Nucleic acid dioxygenase ALKBH1	ALKBH1	CB-6644 only
High	Q9UHK6	Alpha-methylacyl-CoA racemase	AMACR	CB-6644 only
High	P0C7P0	CDGSH iron-sulfur domain-containing protein 3, mitochondrial	CISD3	CB-6644 only
High	M0R0L2	Coenzyme Q8B	COQ8B	CB-6644 only
High	Q8N465	D-2-hydroxyglutarate dehydrogenase, mitochondrial	D2HGDH	CB-6644 only
High	Q5T440	Putative transferase CAF17, mitochondrial	IBA57	CB-6644 only
High	Q86U28	Iron-sulfur cluster assembly 2 homolog, mitochondrial	ISCA2	CB-6644 only
High	Q96AQ8	Mitochondrial calcium uniporter regulator 1	MCUR1	CB-6644 only
High	Q9BV79	Enoyl-[acyl-carrier-protein] reductase, mitochondrial	MECR	CB-6644 only
High	O95822	Malonyl-CoA decarboxylase, mitochondrial	MLYCD	CB-6644 only
High	Q9H019	Mitochondrial fission regulator	MTFR1L	CB-6644 only
High	Q9ULD0	2-oxoglutarate dehydrogenase-like, mitochondrial	OGDHL	CB-6644 only
High	Q8NI37	Protein phosphatase PTC7 homolog	PPTC7	CB-6644 only

B

Accession	Description	Gene Symbol	Group	Expression difference (CTRL-CB6644)	-Log(p-value)
A0A0A0MT83	Isovaleryl-CoA dehydrogenase, mitochondrial	IVD	UP in CB-6644	1,953	2,882
P35914	Hydroxymethylglutaryl-CoA lyase, mitochondrial	HMGCL	UP in CB-6644	1,323	4,354
Q16775	Hydroxyacylglutathione hydrolase, mitochondrial	HAGH	UP in CB-6644	1,213	4,653
Q6NUM9	All-trans-retinol 13,14-reductase	RETSAT	UP in CB-6644	2,385	2,627
Q8N5M1	ATP synthase mitochondrial F1 complex assembly factor 2	ATPAF2	UP in CB-6644	1,312	3,325

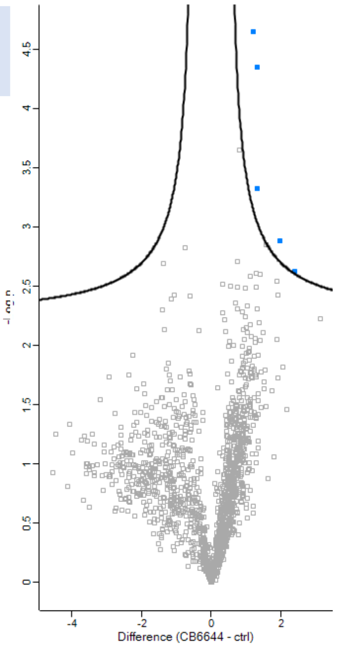

Fig. S8

A

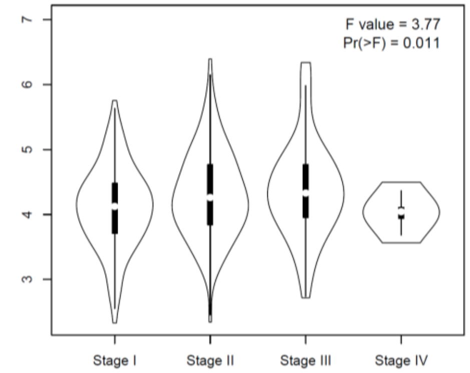

B

Gene Symbol	Gene ID	P-Value (Survival os)
PTDSS2	ENSG00000174915.11	1.82e-9
PIGU	ENSG00000101464.10	1.09e-8
UCK2	ENSG00000143179.12	5.65e-8
KPNA2	ENSG00000182481.8	7.20e-8
HILPDA	ENSG00000135245.9	8.48e-8
MED19	ENSG00000156603.14	1.34e-7
GTPBP4	ENSG00000107937.18	1.80e-7
RUVBL1	ENSG00000175792.11	1.86e-7
CCT5	ENSG00000150753.11	1.90e-7
KB-68A7.1	ENSG00000274225.1	2.01e-7

C

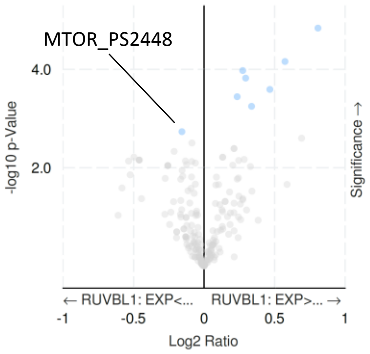

D

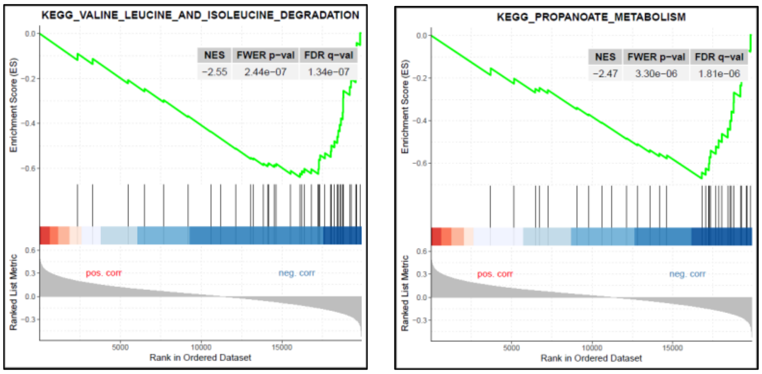

Fig. S9

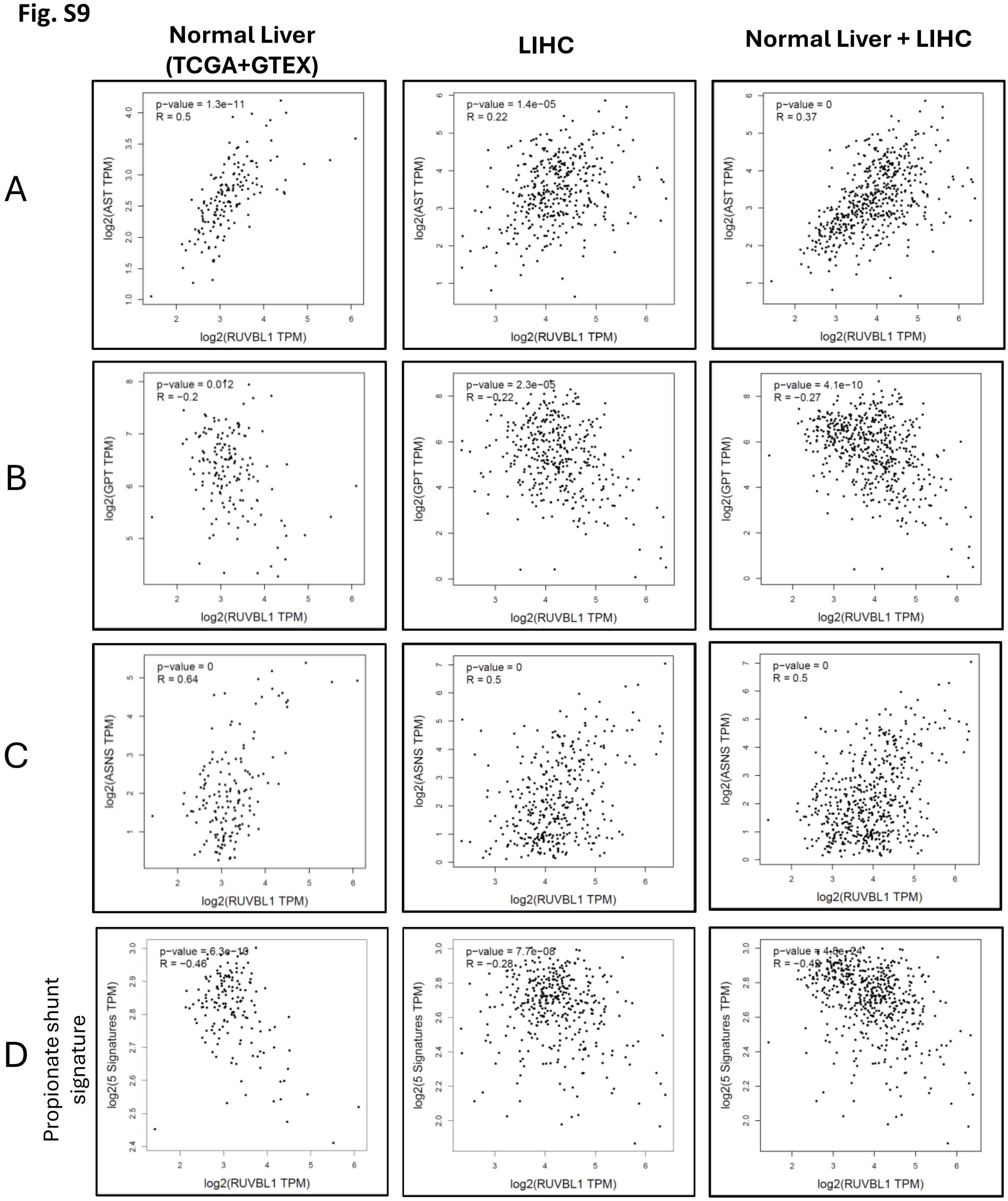

Normal Liver (TCGA+GTEX)
LIHC
Normal Liver + LIHC
A
B
C
D
Propionate shunt signature
p-value = 1.3e-11
R = 0.5
p-value = 1.4e-05
R = 0.22
p-value = 0
R = 0.37
p-value = 0.012
R = -0.2
p-value = 2.3e-05
R = -0.22
p-value = 4.1e-10
R = -0.27
p-value = 0
R = 0.64
p-value = 0
R = 0.5
p-value = 0
R = 0.5
p-value = 6.3e-10
R = -0.46
p-value = 7.7e-08
R = -0.28
p-value = 4.8e-24
R = -0.48
log2(AST TPM)
log2(GPT TPM)
log2(ASNS TPM)
log2(5 Signatures TPM)
log2(RUVBL1 TPM)

Table S1. Metabolic pathways modulated by CB-6644 in Huh7 cells.

Functional annotation of the metabolite set modulated by CB-6644 (0.5uM for 48h) in Huh7 cells. Pathway annotation was performed using the Consensus Path Database. KEGG, Reactome and Wikipathway annotation are reported.

p-value	q-value	pathway	source
6,62E-38	4,11E-36	Central carbon metabolism in cancer - Homo sapiens (human)	KEGG
6,44E-30	2,00E-28	Amino Acid metabolism	Wikipathways
6,63E-25	1,37E-23	Transport of inorganic cations/anions and amino acids/oligopeptides	Reactome
2,71E-24	3,38E-23	Transport of bile salts and organic acids, metal ions and amine compounds	Reactome
2,73E-24	3,38E-23	SLC-mediated transmembrane transport	Reactome
4,41E-24	4,56E-23	Transport of small molecules	Reactome
6,21E-23	5,50E-22	Protein digestion and absorption - Homo sapiens (human)	KEGG
3,18E-22	2,47E-21	Aminoacyl-tRNA biosynthesis - Homo sapiens (human)	KEGG
4,30E-22	2,96E-21	Biochemical Pathways Part I	Wikipathways
3,21E-21	1,99E-20	Glucose Homeostasis	Wikipathways
1,92E-19	1,08E-18	Na+/Cl- dependent neurotransmitter transporters	Reactome
3,08E-19	1,59E-18	Amino acid transport across the plasma membrane	Reactome
5,31E-17	2,13E-16	tRNA Aminoacylation	Reactome
5,31E-17	2,13E-16	Cytosolic tRNA aminoacylation	Reactome
5,31E-17	2,13E-16	Mitochondrial tRNA aminoacylation	Reactome
5,50E-17	2,13E-16	Mineral absorption - Homo sapiens (human)	KEGG
4,39E-16	1,60E-15	Translation	Reactome
2,65E-15	9,13E-15	Metabolism of amino acids and derivatives	Reactome
1,59E-14	5,17E-14	Tryptophan catabolism	Reactome
6,50E-13	2,02E-12	Amino Acid Metabolism Pathway Excerpt (Histidine catabolism extension)	Wikipathways
1,15E-12	3,36E-12	Metabolism of proteins	Reactome
1,23E-12	3,36E-12	ABC transporters - Homo sapiens (human)	KEGG
1,25E-12	3,36E-12	Metabolic reprogramming in colon cancer	Wikipathways
1,80E-12	4,64E-12	Alanine and aspartate metabolism	Wikipathways
9,79E-12	2,43E-11	Metabolism	Reactome
3,61E-11	8,61E-11	Glucagon signaling pathway - Homo sapiens (human)	KEGG
6,46E-11	1,48E-10	Alanine, aspartate and glutamate metabolism - Homo sapiens (human)	KEGG
3,58E-10	7,85E-10	Glucose metabolism	Reactome
3,67E-10	7,85E-10	TCA Cycle and Deficiency of Pyruvate Dehydrogenase complex (PDHc)	Wikipathways
5,23E-10	1,08E-09	Citrate cycle (TCA cycle) - Homo sapiens (human)	KEGG
4,06E-09	8,12E-09	Glycolysis and Gluconeogenesis	Wikipathways
4,94E-09	9,57E-09	Gluconeogenesis	Reactome
5,83E-09	1,10E-08	The citric acid (TCA) cycle and respiratory electron transport	Reactome
1,17E-08	2,14E-08	Phenylalanine and tyrosine metabolism	Reactome
5,63E-08	9,97E-08	Pyruvate metabolism and Citric Acid (TCA) cycle	Reactome

6,24E-08	1,07E-07	Phenylalanine metabolism	Reactome
8,88E-08	1,49E-07	Metabolism overview	Wikipathways
1,02E-07	1,63E-07	Glutamate and glutamine metabolism	Reactome
1,02E-07	1,63E-07	Valine, leucine and isoleucine biosynthesis - Homo sapiens (human)	KEGG
4,23E-07	6,55E-07	Citric acid cycle (TCA cycle)	Reactome
5,93E-07	8,96E-07	Pyruvate metabolism - Homo sapiens (human)	KEGG
7,14E-07	1,05E-06	Glyoxylate and dicarboxylate metabolism - Homo sapiens (human)	KEGG
9,27E-07	1,34E-06	Urea cycle and associated pathways	Wikipathways
1,02E-06	1,43E-06	Metabolism of carbohydrates	Reactome
5,84E-06	8,05E-06	TCA Cycle (aka Krebs or citric acid cycle)	Wikipathways
7,31E-06	9,85E-06	Metabolism of vitamins and cofactors	Reactome
1,07E-05	1,42E-05	Metabolism of water-soluble vitamins and cofactors	Reactome
1,76E-05	2,28E-05	Pantothenate and CoA biosynthesis - Homo sapiens (human)	KEGG
2,60E-05	3,29E-05	Glyoxylate metabolism and glycine degradation	Reactome
4,61E-05	5,71E-05	Branched-chain amino acid catabolism	Reactome
1,38E-04	1,64E-04	Lysine degradation - Homo sapiens (human)	KEGG
1,38E-04	1,64E-04	Glycine, serine and threonine metabolism - Homo sapiens (human)	KEGG
2,81E-04	3,28E-04	Phenylalanine metabolism - Homo sapiens (human)	KEGG
3,39E-04	3,89E-04	Cysteine and methionine metabolism - Homo sapiens (human)	KEGG
6,09E-04	6,87E-04	GPCR downstream signalling	Reactome
1,55E-03	1,72E-03	Signaling by GPCR	Reactome
1,62E-03	1,76E-03	Signal Transduction	Reactome
3,45E-03	3,69E-03	Fatty acid metabolism	Reactome
5,93E-03	6,12E-03	Metabolism of nucleotides	Reactome
5,93E-03	6,12E-03	Post-translational protein modification	Reactome
6,57E-03	6,67E-03	GPCR ligand binding	Reactome

Supplementary references

[1] Chen WW, Freinkman E, Sabatini DM. Rapid immunopurification of mitochondria for metabolite profiling and absolute quantification of matrix metabolites. Nat Protoc 2017;12:2215–31. https://doi.org/10.1038/nprot.2017.104.

[2] Schindelin J, rganda-Carreras I, Frise E, et al. Fiji: an open-source platform for biological-image analysis. Nature Methods 2012;9:676.

[3] Herwig R, Hardt C, Lienhard M, et al. Analyzing and interpreting genome data at the network level with ConsensusPathDB. Nat Protoc 2016;11:1889–907. https://doi.org/10.1038/nprot.2016.117.

[4] Cox J, Mann M. MaxQuant enables high peptide identification rates, individualized p.p.b.-range mass accuracies and proteome-wide protein quantification. Nat Biotechnol 2008;26:1367–72. https://doi.org/10.1038/nbt.1511.

[5] Cox J, Neuhauser N, Michalski A, et al. Andromeda: A Peptide Search Engine Integrated into the MaxQuant Environment. J Proteome Res 2011;10:1794–805. https://doi.org/10.1021/pr101065j.

[6] Tyanova S, Temu T, Sinitcyn P, et al. The Perseus computational platform for comprehensive analysis of (prote)omics data. Nat Methods 2016;13:731–40. https://doi.org/10.1038/nmeth.3901.

[7] Wieckowski MR, Giorgi C, Lebiedzinska M, et al. Isolation of mitochondria-associated membranes and mitochondria from animal tissues and cells. Nat Protoc 2009;4:1582–90. https://doi.org/10.1038/nprot.2009.151.

[8] Morciano G, Sarti AC, Marchi S, et al. Use of luciferase probes to measure ATP in living cells and animals. Nat Protoc 2017;12:1542–62. https://doi.org/10.1038/nprot.2017.052.

[9] Ollion J, Cochennec J, Loll F, et al. TANGO: a generic tool for high-throughput 3D image analysis for studying nuclear organization. Bioinformatics 2013;29:1840–1. https://doi.org/10.1093/bioinformatics/btt276.

[10] **Tang Z**, **Li C**, Kang B, et al. GEPIA: a web server for cancer and normal gene expression profiling and interactive analyses. Nucleic Acids Res 2017;45:W98–102.

[11] Hayashi A, Ruppo S, Heilbrun EE, et al. GENI: A web server to identify gene set enrichments in tumor samples. Computational and Structural Biotechnology Journal 2023;21:5531–7. https://doi.org/10.1016/j.csbj.2023.10.053.

[12] Gao J, Aksoy BA, Dogrusoz U, et al. Integrative analysis of complex cancer genomics and clinical profiles using the cBioPortal. SciSignal 2013;6:l1.

[13] Watson E, Olin-Sandoval V, Hoy MJ, et al. Metabolic network rewiring of propionate flux compensates vitamin B12 deficiency in C. elegans. eLife n.d.;5:e17670. https://doi.org/10.7554/eLife.17670.
